# Is It Possible to Obtain a Product of the Desired Configuration from a Single Knoevenagel Condensation? Isomerization vs. Stereodefined Synthesis

**DOI:** 10.3390/ijms241411339

**Published:** 2023-07-12

**Authors:** Daria Novikova, Tatyana Grigoreva, Vladislav Gurzhiy, Vyacheslav Tribulovich

**Affiliations:** 1 Laboratory of Molecular Pharmacology, St. Petersburg State Institute of Technology (Technical University), St. Petersburg 190013, Russia; 2 Crystallography Department, Institute of Earth Sciences, St. Petersburg State University, St. Petersburg 199034, Russia; vladislav.gurzhiy@spbu.ru

**Keywords:** benzylidene oxindole, *E*-*Z* isomerism, photoinduced isomerization, photostationary state, isomerization kinetics

## Abstract

The biological activity of compounds directly depends on the three-dimensional arrangement of affinity fragments since a high degree of pharmacophore compliance with the binding site is required. 3-Benzylidene oxindoles are privileged structures due to their wide spectrum of biological activity, synthetic availability, and ease of modification. In particular, both kinase inhibitors and kinase activators can be found among 3-benzylidene oxindoles. In this work, we studied model compounds obtained via oxindole condensation with aldehydes and alkylphenones. These condensation products can exist in the form of *E*- and *Z*-isomers and also undergo isomerization in solutions. The factors causing isomeric transformation of these compounds were established. Comparative kinetic studies to obtain quantitative characteristics of UV-driven isomerization were first performed. The results obtained indicate dramatic differences in two subclasses, which should be considered when developing biologically active molecules.

## 1. Introduction

Oxindoles (2-indolinones) are privileged structures in medicinal chemistry due to their synthetic availability and the ease of introducing various substituents to the scaffold. Among these compounds, natural and synthetic derivatives of 3-alkenyloxindole are of particular interest as a promising platform for the development of targeted anticancer agents [1,2]. An interesting property of 3-alkenyloxindole derivatives is that they can act as both AMPK activators [3] and AMPK inhibitors [4]; such universal activity was also found for 3-alkenyloxindoles, which can act as MDM2 and P-gp inhibitors [5,6]. However, an essential point in the synthesis of 3-alkenyloxindoles is the stereochemistry of the final product.

The interest in the configuration of (*E*)- and (*Z*)-1,3-dihydro-2-oxindole-3-ylidenes arose when R.A. Abramovich and D.A. Hey first suggested the existence of two configurational isomers of benzylidene-1,3-dihydroindol-2-one [7]. While the principle of spatial correspondence of ligand pharmacophores to the active site underlies rational drug design [8,9], the double bond in position three of the oxindole core determines the *E*/*Z* isomerism of these compounds leading to differences in biological activity due to the geometry of individual isomers [10].

**Figure 1:**
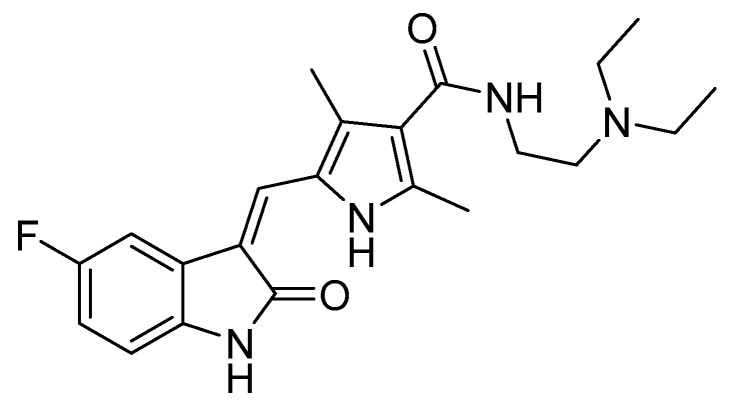
Structure of sunitinib (*N*-[2-(Diethylamino)ethyl]-5-[(Z)-(5-fluoro-1,2-dihydro-2-oxo-3*H*-indol-3-ylidene)methyl]-2,4-dimethyl-1*H*-pyrrole-3-carboxamide).

In recent years, there has been a steady interest in isomerization processes of active 3-alkenyloxindoles associated with the need to understand further transformation and the interpretation of data from in vitro and in vivo studies. Sunitinib (Figure 1), a multitarget tyrosine kinase inhibitor [11], has become the most studied 3-alkenyloxindole derivative, for which the process of photoinduced isomerization was studied in detail [12,13] and a method for quantifying the drug concentration in plasma considering isomerization was developed for clinical use [14].

Despite the huge therapeutic potential of both AMPK activators [15] and AMPK inhibitors [16] in clinical trials, direct AMPK modulators are not currently available for patients. We believe that 3-benzylidene oxindoles are a promising platform for the development of selective AMPK modulators possessing activatory or inhibitory effects depending on the structure. In this work, we first performed isomerization studies of various 3-alkenyloxindoles in order to demonstrate the possibility of obtaining a product of the required configuration from a common Knoevenagel condensation. We also evaluated the influence of solvent used, pH of the media, and light, which are factors that are an integral part of the work-up in organic synthesis that can provoke changes in the initial isomeric state.

## 2. Results and Discussion

3-Benzylidene oxindoles are most often obtained via the Knoevenagel reaction of various alkylphenones or benzaldehydes and oxindoles obtained via the Wolff–Kishner reaction from the corresponding isatins [17]. The reaction of oxindoles with benzaldehydes proceeds easily and in high yields, while alkylphenones are less active in the Knoevenagel reaction. But even in this case, condensation products are formed in good yields when the reaction is carried out under classical conditions. Alcohols (ethanol and methanol) are most often used as solvents, while classical amines (pyridine, piperidine, β-alanine, and ammonia) are used as bases [18]. However, a lot of modifications have been proposed since the pioneering studies by Knoevenagel in 1898, including those suggesting green protocols [19,20], solvent-free conditions [21,22], and quantitative conversion [23]. Such Knoevenagel condensation products could not only be final active molecules or intermediates [24] but also represent a monomer in conjugated polymers for solar cell devices [25] or tunable molecular motors [26].

Alternative methods are based on the construction of the carbon skeleton by ring formation. Therefore, substituted 3-benzylidene oxindoles can be obtained via palladium-catalyzed intramolecular alkenylation of substituted *N*-cinnamoylanilines [27] or through the ring formation reaction of 2-(alkynyl)aryl isocyanates with organoboron reagents [28]. These methods allow for obtaining isomerically pure products in the ideal case, although the possibility to control the stereoselectivity of the reaction by varying temperature was reported [29]. In addition, 3-benzylidene oxindoles can be obtained via the McMurry reaction, but this method is more often used to obtain benzophenone derivatives [30].

As for 3-benzylidene oxindole derivatives, there are attempts to develop stereoselective Knoevenagel protocols. It was found that CeO_2_ can be an efficient catalyst for selective synthesis of (*E*)-3-alkenyl-oxindoles from oxindole and aldehydes, suggesting the heterogeneous catalytic C3-selective alkenylation reaction of oxindole for both aromatic and aliphatic aldehydes [31]. The use of a biocatalyst such as porcine pancreas lipase (PPL) in the Knoevenagel condensation of oxindole and various aromatic aldehydes allows one to perform the reaction with high selectivity towards the *E* configuration [32]. Also, iron-catalyzed aerobic oxidative condensation of oxindoles with benzylamines was reported to result in selective formation of (*E*)-3-alkylideneindolin-2-ones [33], although this is not a Knoevenagel condensation. Contrariwise, the Ti(O*i*Pr)_4_/pyridine system, which was first proposed by Robichaud and Liu [34] to provide Knoevenagel olefin products, was further used for selective synthesis of (*Z*)-3-alkylideneoxindoles from unsymmetrical ketones, but not aldehydes [35]. However, the stereoselectivity reported in all the listed works in not high enough as required in drug design, which slightly reduces the value of these innovative developments. Perhaps, the really stereoselective synthesis is not possible for these objects due to the reasons discussed below.

**Scheme 1:**
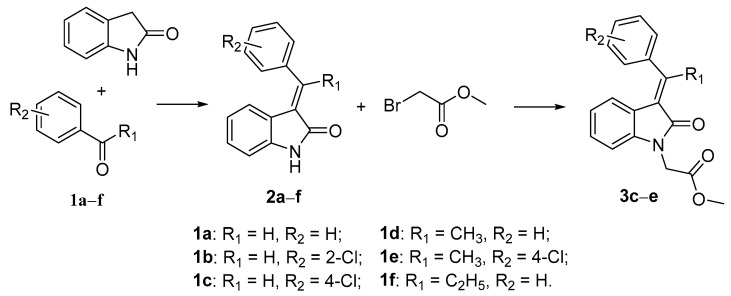
Synthesis of model 3-alkylidene oxindoles.

To study isomerization of 3-benzylidene oxindole derivatives, we obtained a series of model compounds via a convenient procedure for Knoevenagel condensation used previously: boiling of a mixture of oxindole, carbonyl compound, and pyrrolidine in toluene with azeotropic distillation of the resulting water using a Dean–Stark apparatus [17]. Further modification at the nitrogen atom was carried out via alkylation with bromoacetic ester (Scheme 1). The main disadvantage of such a classical procedure is the possible formation of a mixture of two geometric isomers.

Despite the fact that in some cases of the above Knoevenagel procedure the isomeric ratio is close to 2:1, we found that *E*-isomers are predominantly formed both when using aldehydes and ketones. Before the study, we needed to isolate individual isomers; for this we used the obtained mixtures. Due to significant differences in the geometry of the structures, the isomers resolve well on TLC and HPLC (see Supplementary Materials, Table S1). An interesting feature of the studied compounds is the inversion of retention by silica gel when passing from derivatives based on aldehydes to alkylphenone condensation products. While *Z*-isomers of aldehyde derivatives show higher values of the retardation factor than *E*-isomers, *E*-isomers of alkylphenone derivatives are faster travelling than *Z*-isomers. A similar but reversed situation is observed in HPLC analysis.

**Figure 2:**
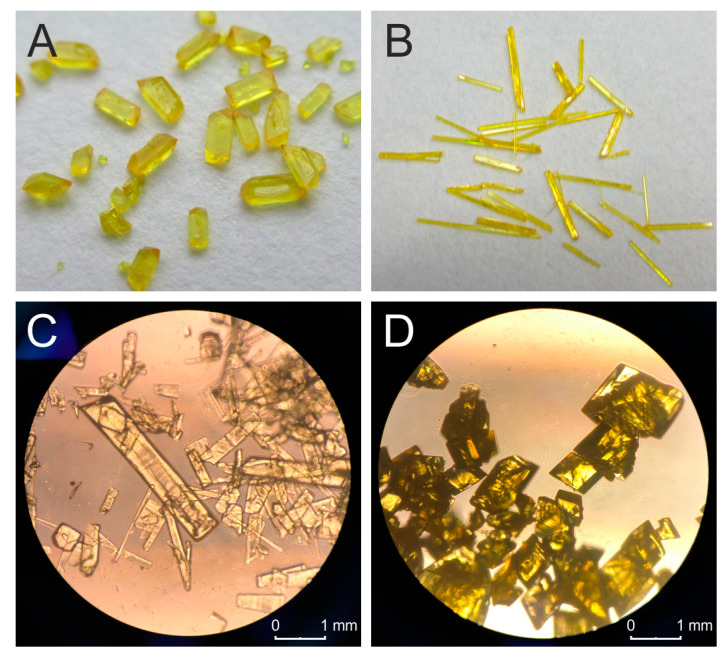
Different crystal structures of isomer pairs: pictures of *E*-**2a** crystals (**A**) and *Z*-**2a** needles (**B**); micrographs of *E*-**3e** plates (**C**) and *Z*-**3e** crystals (**D**).

Before the study, we also carefully investigated physicochemical characteristics of the obtained *E*- and *Z*-isomers of 3-benzylidene oxindoles. The isomers showed significant differences in melting points and solubility, explained by differences in the geometries. Differences in the crystal structure are the most significant (Figure 2), in some cases allowing separation of isomers via the Pasteur method.

**Figure 3:**
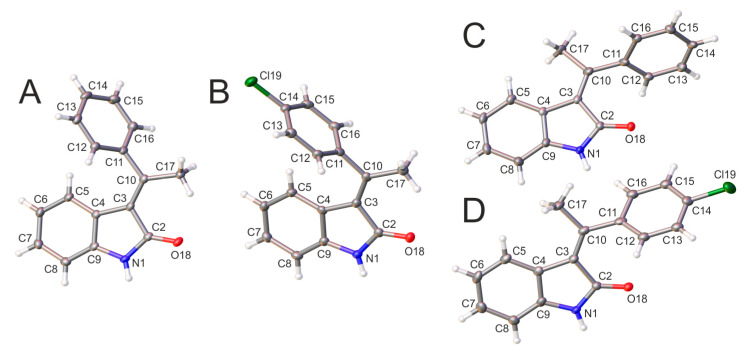
Representation of *E*-**2d** (**A**), *E*-**2e** (**B**), *Z*-**2d** (**C**), and *Z*-**2e** (**D**) structures via X-ray analysis. Disordered fragments in the structures of *Z*-isomers are omitted. Legend: carbon, nitrogen, oxygen and chlorine atoms are given in grey, light-blue, red and green, respectively.

**Figure 4:**
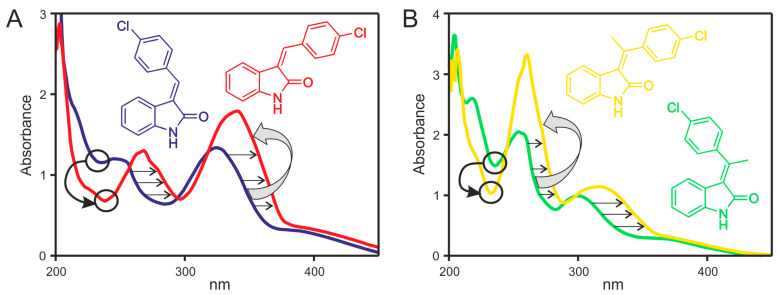
Distinctive features in the absorption spectra of *E*/*Z* isomers as exemplified by isomer pairs **2c** (**A**) and **2e** (**B**). The color of the curve corresponds to the isomer in the same color.

Structural differences are also clearly manifested in the NMR spectra (Figures S1 and S2; Table S2), and this allows one to use this method to establish the configuration of 3-benzylidene oxindoles without recruiting methods of X-ray structure analysis (Figure 3). Since UV spectroscopy is a convenient and more reliable method for the stereochemical determination of alkenes [36], we also studied the spectral characteristics of the isolated *E*- and *Z*-isomers (Figure S3) and revealed features in the absorption spectra that can be used for the rapid isomer identification of 3-benzylidene oxindole series (Figure 4, a detailed description is given in the Supplementary Materials).

**Scheme 2:**
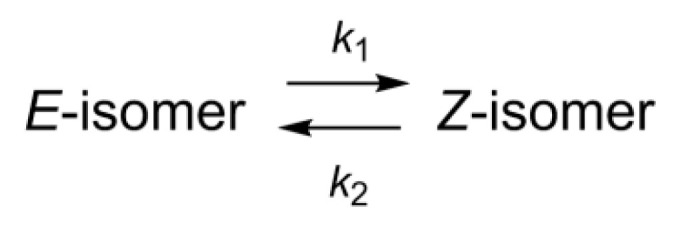
Isomerization reaction and the corresponding constants.

However, the most important feature of 3-benzylidene oxindoles is their tendency to isomerization. Numerous sources indicate that 3-benzylidene oxindoles are subjected to isomerization in solutions. Sun et al. observed an equilibrium between *Z*- and *E*-isomeric forms of their tyrosine kinase inhibitors based on 3-(substituted benzylidenyl)indolin-2-ones in polar solvents or in the presence of light [37]. Since the ability of *cis*-*trans* photoisomerization is inherent in many alkenes, we first studied the isomeric ratio of the studied compounds in solutions under UV irradiation at the photostationary state, when the rate of the direct and reverse processes is the same (Scheme 2). We used HPLC analysis to study the ratio of isomers by constructing calibration curves before each experiment. A simple comparison of peak areas can lead to an incorrect interpretation of the experimental data [38] since at 254 nm, the wavelength most often used for analysis, 3-benzylidene oxindole isomers have very different absorbing capacities (for example, the molar absorptivity coefficient for *E*-**2c** and *Z*-**2c** is 13,177 and 11,380 L/mol·cm, respectively).

According to the obtained data, the position of the photostationary state only slightly depends on the solvent (Table 1) since the tendency towards the predominance of *Z*-isomer for aldehyde derivatives and of *E*-isomer for alkylphenone derivatives remains unchanged. In general, the isomeric ratios range from 1:2 to 1:4. In the case of the 2-Cl-substituted derivative, we can observe an inversion of the isomer ratio in a number of solvents, which is apparently associated with steric factors. Probably, the presence of a bulky electronegative substituent in the second position makes the *Z*-configuration energetically less favorable. The equilibrium ratios that were established in the solutions a day after the experiments differed slightly from the photostationary one, but by no more than 5%.

Our further efforts were aimed at identifying the factors contributing to isomerization and assessing the rate of the processes. In order to establish whether it is possible to initiate isomerization of 3-benzylidene oxindoles and determine the intensity of the process, we carried out a series of experiments consisting of the dissolution of pure isomers (*E*- and *Z*-) in a number of the most commonly used solvents without access to light and found that only benzaldehyde derivatives underwent isomerization. Thus, in toluene, acetone, and dichloromethane, the formation of a small amount of the second isomer was observed; in alcohols (methanol and isopropanol), there was a more significant isomeric transformation; the largest amounts of the second isomer were formed in DMF solutions.

At the same time, we cannot say that it is the polarity of the solvent that initiates the isomerization process, while nitrogen substitution promotes the studied process in the absence of light (for example, in acetone the observed isomer ratio a month later was 1/99 for *Z*-**2c** and 10/90 for *Z*-**3c** when the *Z*-isomer was initially dissolved). Regarding solvents common for NMR, isomerization of aldehyde derivatives in chloroform was in the range of 1–5%, and in DMSO—2–10%. Since no significant isomerization was observed for alkylphenone derivatives in chloroform or in DMSO, it is of particular interest to further establish the causes of isomerization observed in NMR spectra during long-term two-dimensional experiments on minor *Z*-isomers (Figure S2). Apparently, the isomerization of benzylidene oxindoles is initiated not only by the solvent and light but also by the magnetic field used when recording NMR spectra. In this case, we noted that *Z*-isomers are especially sensitive to it.

**Figure 5:**
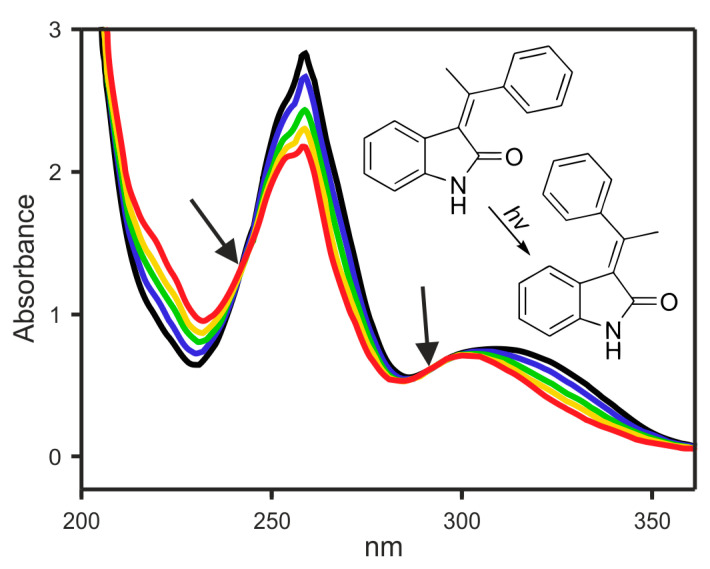
Spectrophotometric monitoring of *Z* → *E* isomerization for **2d** in methanol (arrows indicate isosbestic points). Black curve corresponds to the initial time point (pure *Z*-isomer); blue, green, and yellow curves correspond to intermediate time points (*Z*- and *E*-isomer mixtures); red curve corresponds to the time point when the equilibrium between the isomers is reached (photostationary state).

In the literature, it was suggested that the UV-driven isomerization process may occur with the formation of by-products and/or degradation products. We showed, via HPLC analysis, that under irradiation conditions used (7 W UV lamp) no by-products were noticeably formed during all experiments performed. The absence of side reactions during photoisomerization was additionally confirmed by the presence of isosbestic points on the absorption spectra during spectrophotometric monitoring of the process (Figure 5). However, destruction processes obviously occur when irradiated with high-power light sources.

**Figure 6:**
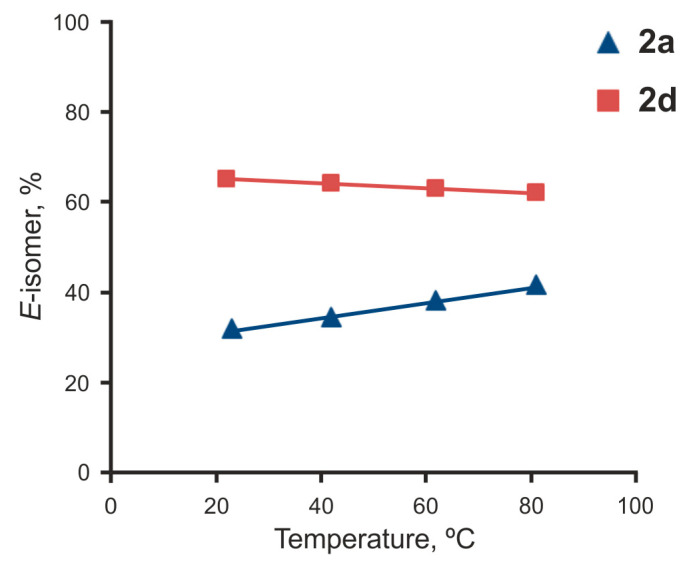
Dependence of the photostationary state on temperature as exemplified by **2a** and **2d** in acetonitrile.

The position of the photostationary state was also found to depend on temperature. For derivatives based on aldehydes, a linear increase in the content of *E*-isomer with increasing temperature is observed, which is consistent with the previously obtained data [38], while the *E*-isomer content of alkylphenone derivatives decreases (Figure 6). However, the most interesting result from the applied point of view is the possibility of complete conversion of *E*-isomer into *Z*-isomer or vice versa.

Analyzing all the obtained data that characterize the studied compounds, we did not find obvious solutions that allow us to carry out this kind of reaction. An interesting statement by Cheng et al. is that their (*E*)-3-alkylideneoxindoles were easily converted into (*Z*)-3-alkylideneoxindoles under UV irradiation [39]. Upon closer examination of the reaction conditions, it appears that the authors added three equivalents of iodine, probably leading to the formation of interactions between the carbonyl groups present in those molecules, which cannot be applied to the studied compounds.

It should be separately noted that oxindole, which forms the backbone of the studied molecules, can be ionized in the presence of bases. Our molecules have much fewer possibilities; for example, compounds of series **2** lack acidic protons at C_3_, and compounds of series **3**, in addition, have a substituent instead of a proton at the nitrogen atom. However, upon dissolution in alkaline methanol without light initiation, already 10 min after, the equilibrium with the *E*-isomer proportion of 90–95% is established for all individual isomers of the studied compounds. In aprotic solvents such as DMSO and DMF, and when potassium carbonate is used as the base, no such isomerization is observed. At the same time, no significant changes in the isomeric ratio were observed upon dissolution in protic solvents at acidic pH values.

In the literature, one can find many studies on isomerization kinetics of sunitinib and its derivatives, which is associated with the need to develop clear procedures for the quantitative analysis of medical substances of this class [12,14], while 3-benzylidene oxindoles have been little studied. Considering that both the solvent and the light source have a significant effect on the isomerization process, we first conducted a study of the comparative kinetics of model 3-alkylideneoxindoles in methanol under UV irradiation using the method of HPLC to quantify the process in order to determine how the structure of the compounds affects the isomerization rate.

**Figure 7:**
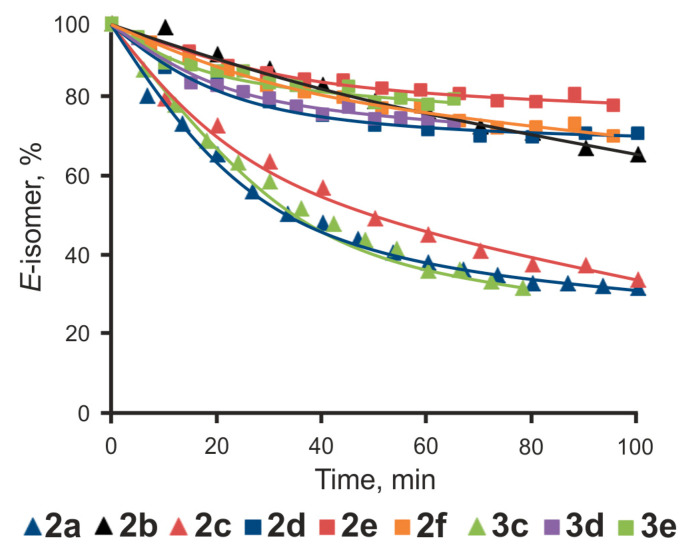
Kinetic curves of *E* → *Z* isomerization of 3-benzylidene oxindoles in methanol.

First of all, we evaluate *E*-isomer to *Z*-isomer transition, for which a solution of pure *E*-isomer was irradiated with a low-power UV source. With such an experiment, the studied compounds reach an isomer ratio close to that observed at the photostationary state, or corresponding to the photostationary state within 120 min. The obtained kinetic dependences (Figure 7) were processed considering the equilibrium constant *K*, calculated by the formula using the data obtained earlier:

*K* = *k*_1_/*k*_2_ =*Z*_eq_/*E*_eq_, (1)

where *k*_1_ is the rate constant of *E* → *Z* isomerization; *k*_2_ is the rate constant of *Z*→*E* isomerization; and *E*_eq_ and *Z*_eq_ are the equilibrium concentrations of *E*- and *Z*-isomers, respectively. Since for all studied compounds 0.01 < *K* < 100, the desired process cannot be reduced to a simple first-order reaction [40]. In the case of the considered equilibrium systems, the *E* → *Z* transition process is described by the following equation:

*ln*(*EK* − *Z*) = *ln*(*E*_0_*K* − *Z*_0_) − (*k*_1_ + *k*_2_)*t*. (2)

The obtained dependences were used to estimate the rate constants of direct (*E* → *Z*) and reverse (*Z* → *E*) isomerization processes (Table 2).

The analysis of experimental data showed that the isomerization rate of *E*-isomers for aldehyde derivatives is significantly higher than that for alkylphenone derivatives. In this case, when passing to *N*-substituted derivatives, the isomerization rate increases significantly. The introduction of an electron-withdrawing substituent (chlorine atom) into position four of the benzylidene fragment slows down all isomerization processes for both aldehyde and alkylphenone derivatives. At the same time, the data for compound **2b** fall significantly out of the entire picture; this, as well as the equilibrium constant close to 1, can be explained by steric hindrance caused by the chlorine atom at position two.

**Figure 8:**
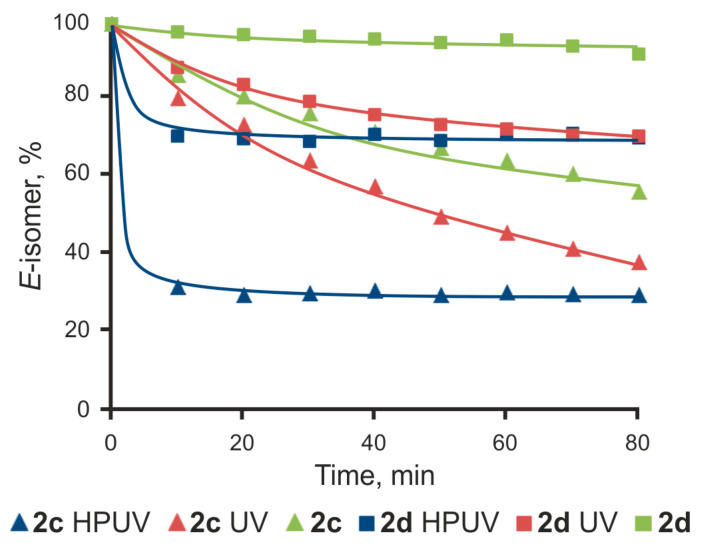
Kinetic curves of *E* → *Z* isomerization in methanol under different light sources used.

In addition, we evaluated how the power and wavelength of the light source affect the isomerization process of the studied compounds. In the case of a high-pressure UV lamp, an equilibrium mixture was readily formed; after 10 min of the experiment, the isomer ratio was close to the equilibrium. However, when irradiated with a table lamp, significant changes in the isomeric composition were also observed. At the same time, daylight irradiation had a more significant effect on the isomerization processes on the aldehyde derivative (more than 30% *E*-**2c** was isomerized in 80 min of the experiment at the photostationary ratio of 31/69), while the alkylphenone derivative turned out to be less susceptible to the action of daylight (in 80 min of the experiment about 8% *E*-**2d** was isomerized at the photostationary ratio of 71/29) (Figure 8).

## 3. Materials and Methods

### 3.1. Chemistry

All starting compounds and reagents used are commercially available. 2-Oxindole was obtained according to the previously described procedure [41]. Reactions were monitored via TLC on Silica gel 60 F254 plates (Merk, Boston, MA, USA) using n-hexane/ethyl acetate. Purification of products and isolation of individual isomers was carried out using an Isolera Four flash chromatograph on SNAP KP-Sil 100 g cartridges (Biotage, Uppsala, Sweden) with n-hexane/ethyl acetate eluent.

**General method for obtaining unsubstituted benzylidene oxindoles (2a–f):** 2-Oxindole (15 mmol) was suspended in 50 mL of toluene, the corresponding aldehyde or ketone (18 mmol, 1.2 equiv.) and 2.5 mL of pyrrolidine (30 mmol, 2 equiv.) were added and refluxed with a Dean–Stark trap for 0.25–3 h depending on the activity of the carbonyl component. The progress of the reaction was monitored via TLC. The reaction mixture was cooled and the solvent was evaporated under reduced pressure. The residue was triturated in a small amount of ethyl acetate and left overnight. The precipitate formed was filtered off and dried. The crude product (predominantly *E-*isomer) was purified via recrystallization from ethyl acetate/n-hexane mixtures.

**General method for obtaining *N*-substituted benzylidene oxindoles (3c–e):** Benzylidene oxindole **2c**–**e** (10 mmol) was dissolved in 50 mL of THF and cooled on ice; then, 0.5 g of sodium hydride (60%, 12.5 mmol, 1.25 equiv.) was added under stirring. The mixture was kept for 30 min at rt. Then, methyl bromoacetate (14 mmol, 1.4 equiv.) dissolved in 10 mL THF was added dropwise. The reaction mixture was stirred for 3 h. Next, the solvent was evaporated under reduced pressure and 50 mL of water was added to the residue. The resulting precipitate was filtered off and dried. The crude product was purified via flash chromatography.

Characterization of the studied compounds is provided in the Supplementary Materials.

### 3.2. NMR Analysis

^1^H and ^13^C NMR spectra were recorded on a Bruker Avance III 400 (400 MHz) device in CDCl_3_. To confirm the spatial configuration of the compounds (*E-* or *Z*-isomer), crystallographic data or ^1^H-^1^H NOESY spectra were considered. Pictures of the spectra are presented in the Supplementary Materials.

### 3.3. Spectrophotometric Analysis

Spectrophotometric study was conducted on a UV-1800 device (Shimadzu, Kyoto, Japan). For this purpose, an initial solution with a concentration of 0.4 mg/mL in methanol was prepared. Then, the following concentrations were used to build calibration curves: *C* = 0.0333; 0.0222; 0.01665; 0.0111; and 0.008325 mg/mL. The obtained curves allowed us to confirm the compliance with the Bouguer–Lambert–Beer law and to determine the molar attenuation coefficient (ε). The original spectra are given in the Supplementary Materials.

### 3.4. HPLC Analysis

HPLC analysis was used to evaluate the isomer ratio. For this purpose, calibration curves were built based on a series of serial dilutions (at least three) for one isomer within the isomer pair. Each concentration was analyzed three times. The concentration of the second isomer formed during isomerization process was determined by the formula:

*C*(isomer 2) = *C*_0_(isomer 1) − *C*(isomer 1). (3)

Calibration curves were built immediately before the given experiment for the given compound. HPLC analysis was performed on a LC-20 Prominence (Shimadzu) using a Nucleodur PolarTec column (Macherey-Nagel, Düren, Germany) with a length of 150 mm, an internal diameter of 3.0 mm, a particle size of 3 µm, in acetonitrile—0.1% trifluoroacetic acid (from 55/45 to 70/30), with a flow rate of 0.4 mL/min, and an oven temperature of 40 °C.

### 3.5. LC-MS Analysis

LC-MS analysis was performed on a LCMS-2020 device (Shimadzu) with a single quadrupole detector under positive mode, electrospray ionization (ESI), using a Nucleodur PolarTec column (Macherey-Nagel), with a length of 150 mm, an internal diameter of 3.0 mm, a particle size of 3 µm, in the system of acetonitrile–0.1% trifluoroacetic acid (55/45), with a flow rate of 0.15 mL/min, and an oven temperature of 40 °C. The original spectra of isomeric pairs (arbitrary *E*/*Z* ratio) are given in the Supplementary Materials.

### 3.6. Isomerization Studies

To study the isomerization process of benzylidene oxindoles in solutions, a 15 mL quartz reactor equipped with a jacket and an immersion thermometer was used. To maintain a constant temperature during irradiation, tap water was used to provide a temperature of 15 ± 2 °C in the irradiated solution. When conducting temperature experiments, a Viscotherm VT2 recirculator chiller (Anton-Paar, Houston, TX, USA) was used. The light source was placed at a distance of 15 cm from the reactor. The following light sources were used: Puritec HNS S 7 W lamp (Osram, Wilmington, MA, USA), installed into a common table luminescent illuminator, with a dominant wavelength of 254 nm, a candlepower of 7800 cd, and a UVC of 1.8 W; a mercury–quartz table irradiator OKN-11 (SEMAZ, Sverdlovsk, USSR) with a DRT220 lamp, 850 W at 220 V, with a dominant wavelength of 254 nm; and a Dulux S 7 W lamp (Osram), installed into common table luminescent illuminator, with a luminous flux of 400 lm and T = 3000 K. Calculation of the kinetic parameters is given in the Supplementary Materials.

### 3.7. X-ray Analysis

X-ray data were obtained on a Rigaku Oxford Diffraction SuperNova Atlas diffractometer (Agilent, Santa Clara, CA, USA) at a temperature of 100 K using monochromated microfocused Cu*Kα* radiation. The crystal structures of *E-***2d**, *Z-***2d**, *E-***2e,** and *Z-***2e** were deposited at the Cambridge Crystallographic Data Centre (CCDC 2,171,213–2,171,216). Details are given in the Supplementary Materials.

## 4. Conclusions

In this work, characteristics of aldehyde and alkylphenone derivatives of 3-benzylidene oxindoles, which are potential scaffolds for developing both AMPK activators and inhibitors, were studied in detail. It was shown that these structures can undergo not only UV-driven isomerization but also light-, solvent-, and pH-driven isomeric transformations.

The obtained data clearly indicate the advances of using isomerization for obtaining products of a desired configuration from the classical Knoevenagel condensation. It was shown that equilibrium between aldehyde derivatives of 3-benzylidene oxindoles can be shifted towards the required isomer via isomerization under UV irradiation or in basic medium. Thus, not only classical *Z*-configured kinase inhibitors [42,43] can be obtained based on these 3-benzylidene oxindoles but also recently reported inhibitors in the *E* form [44,45]. Sophisticated stereoselective methods are partly justified only for obtaining Z-isomers of alkylphenone derivatives since the high rate of isomerization in solutions was shown for them. However, no such active molecules have been reported yet.

Our comprehensive isomerization study is the first example of combining knowledge regarding 3-benzylidene oxindole isomerization for the application in synthetic chemistry. Despite the difficulties, which a researcher may meet with when developing drugs based on 3-benzylidene oxindoles, these structures are still privileged molecules due to their wide spectrum of biological activity, synthetic availability, and ease of modification.

## Figures and Tables

**Table 1:** Isomer ratios (*E*/*Z*) observed at the photostationary state in various solvents.

Compound	ACN	DMF	Acetone	AcOEt	MeOH	iPrOH	CH_2_Cl_2_	CHCl_3_	Toluene
**2a**	30/70	22/78	31/69	28/72	32/68	33/67	29/71	27/73	35/65
**2b**	55/45	40/60	45/55	50/50	48/52	51/49	43/57	42/58	49/51
**2c**	29/71	21/79	31/69	30/70	31/69	34/64	28/72	25/75	32/68
**2d**	65/35	79/21	74/26	70/30	71/29	69/31	73/27	75/25	68/32
**2e**	72/28	76/24	75/25	72/28	76/24	70/30	73/27	73/27	70/30
**2f**	70/30	73/27	69/31	70/30	69/31	68/32	79/21	77/23	68/32
**3c**	29/71	21/79	29/71	27/73	30/70	30/70	28/72	26/74	32/68
**3d**	69/31	81/19	74/26	72/28	75/25	75/25	77/23	79/21	70/30
**3e**	73/27	79/21	73/27	74/26	77/23	72/28	75/25	76/24	72/28

**Table 2:** Isomerization kinetics of 3-benzylidene oxindoles under UV irradiation.

Compound	*K*	*k*_1_ × 10^3^ (s^−1^)	*k*_2_ × 10^3^ (s^−1^)
**2a**	2.125	0.445	0.210
**2b**	1.083	0.082	0.075
**2c**	2.226	0.300	0.135
**2d**	0.408	0.206	0.506
**2e**	0.316	0.082	0.260
**2f**	0.449	0.126	0.280
**3c**	2.333	0.407	0.175
**3d**	0.333	0.213	0.640
**3e**	0.299	0.129	0.432

## Data Availability

Not applicable.

## Supplementary Materials

The following supporting information can be downloaded at: https://www.mdpi.com/article/10.3390/ijms241411339/s1, References [46,47,48,49,50,51] are cited in the supplementary materials.
