# Supplementary material for: Is It Possible to Obtain a Product of the Desired Configuration from a Single Knoevenagel Condensation? Isomerization vs. Stereodefined Synthesis
Source: Int J Mol Sci. 2023 Jul 12;24(14):11339. doi: 10.3390/ijms241411339 (PMC10379276; doi:10.3390/ijms241411339)
Supplement: Supplementary file 1 [file ijms-24-11339-s001.zip › ijms-2470542-supplementary.pdf]

Supplementary Materials

Is it possible to obtain a product of the desired configuration from a single Knoevenagel condensation? Isomerization vs. stereodefined synthesis

Daria Novikova[a], Tatyana Grigoreva[a], Vladislav Gurzhiy[b], and Vyacheslav Tribulovich[a]

[a]Laboratory of Molecular Pharmacology, St. Petersburg State Institute of Technology (Technical University), St. Petersburg, Russia
[b]Crystallography Department, Institute of Earth Sciences, St. Petersburg State University, St. Petersburg, Russia

Corresponding authors: Daria Novikova, Vyacheslav Tribulovich
postal address: Laboratory of Molecular Pharmacology, St. Petersburg State Institute of Technology (Technical University), 26 Moskovskii pr., St. Petersburg, 190013 Russia
phone: +7 (812) 494-92-66
fax: +7 (812) 316-46-48
e-mail: dc.novikova@gmail.com (D.N.), tribulovich@gmail.com (V.T.)

Content

TLC and HPLC data	S3
NMR analysis	S4
Characterization of studied compounds	S7
^{1}H and ^{13}C NMR spectra	S12
UV-Vis analysis	S30
Spectrophotometric characteristics	S32
UV-Vis spectra	S34
LC-MS spectra of isomeric pairs	S44
Kinetics data	S62
X-ray data	S71
References	S76

TLC and HPLC data

Table S1. Retention characteristics of individual isomers of model compounds (values for fast travelling isomers are given in bold).

Compound	Isomer	R_f a)	Retention time b), min
**2a**	*E*	0.34	**14.197**
	Z	**0.54**	15.836
**2b**	*E*	0.24	**17.820**
	Z	**0.28**	21.885
**2c**	*E*	0.32	**22.724**
	Z	**0.62**	25.790
**2d**	*E*	**0.54**	17.090
	Z	0.38	**13.604**
**2e**	*E*	**0.26**	25.549
	Z	0.24	**19.116**
**2f**	*E*	**0.33**	22.447
	Z	0.14	**17.329**
**3c**	*E*	0.49	**22.481**
	Z	**0.69**	28.281
**3d**	*E*	**0.45**	18.650
	Z	0.28	**15.098**
**3e**	*E*	**0.37**	27.183
	Z	0.34	**21.579**

a) eluted with hexane:ethyl acetate 6:1,

b) eluted with ACN–0.1% TFU 55:45, 0.15 mL/min, Nucleodur PolarTec column.

NMR analysis

The unsubstituted oxindole motif of 3-benzylidene oxindoles has a very characteristic pattern in the ^{1}H NMR spectra. It is represented by a pair of doublets (H_4 and H_7) and a pair of triplets (H_5 and H_6). In most cases, H_4 and H_6 proton signals are located in a lower field compared with H_7 and H_5 signals (Figure S1). The correlation of signals can be easily obtained from ^{1}H-^{1}H NOESY spectra, where interactions of all neighboring hydrogen atoms are clearly visible, including the characteristically broadened hydrogen atom at the nitrogen atom of the oxindole core (Figure S2). However, the characteristic pattern of the arrangement of oxindole protons is disrupted in the spectra of *E*-isomers obtained based on alkylphenones: contrary to predictions, H_4 signal is significantly shifted to the high field region. Also, signals of ortho-protons within the benzylidene fragment are in a higher field than the rest group of aromatic protons for alkylphenone derivatives, while for benzaldehyde derivatives, on the contrary, ortho-proton signals are shifted to a lower field.

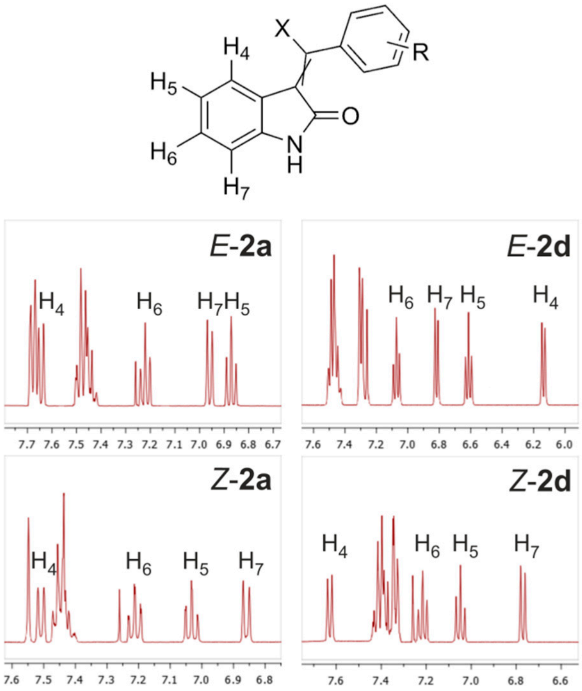

Figure S1. Patterns of oxindole proton signals in NMR spectra (as exemplified by isomeric pairs of **2a** and **2d**).

Analyzing the data of NMR spectra, it is possible to identify characteristic signals that allow an unambiguous interpretation of *E*- and *Z*-isomers of 3-benzylidene oxindoles. Thus, the signals of ortho-protons turn out to be the most characteristic for the derivatives based on benzaldehydes: in the case of *Z*-isomers, a significant shift to the lower field region is observed in comparison with *E*-isomers. The position of the proton at the double bond is also characteristic: in the case of *Z*-isomers, it is shifted to the high field region. The authors of the

studies [2, 3] suggest that the vinylic proton is more deshielded in *E*-isomers due to the carbonyl influence, whereas ortho-benzylidene protons are more deshielded in *Z*-isomers for the same reason.

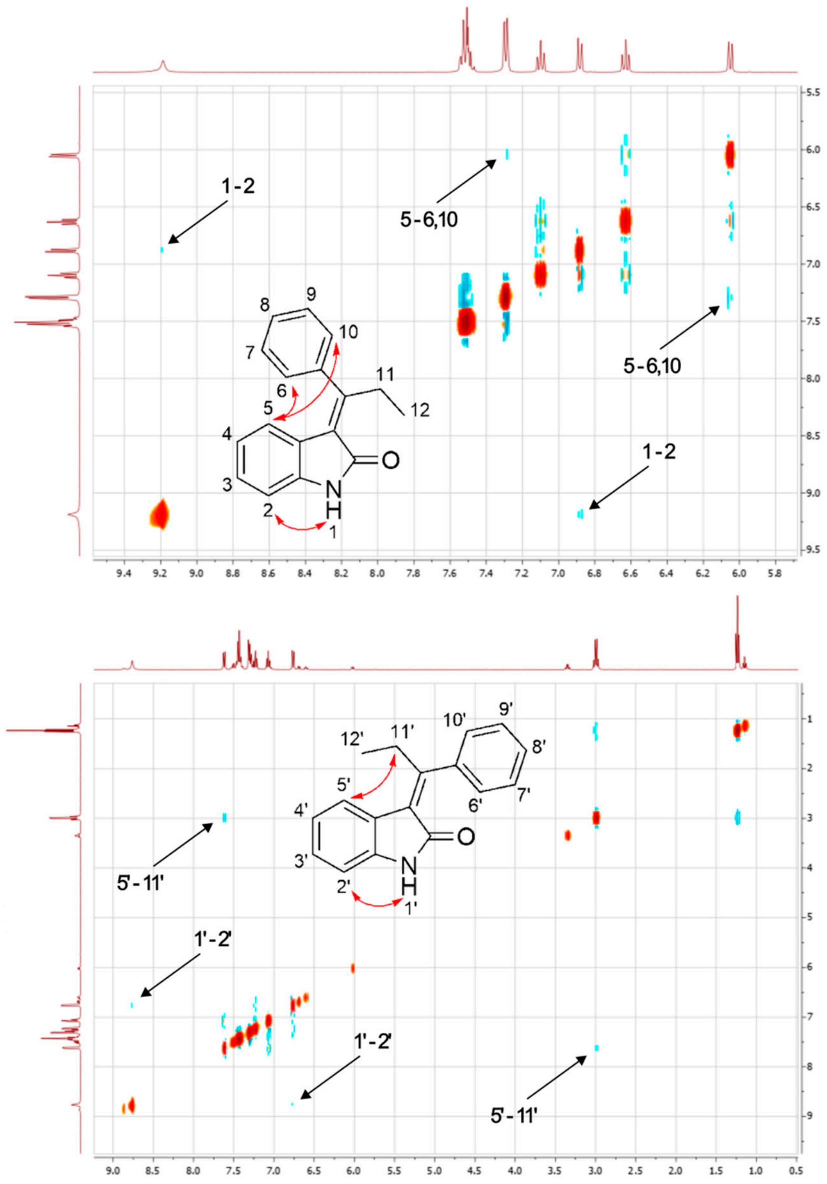

Figure S2. ^{1}H-^{1}H NOESY spectra of *E*- and *Z*-phenylpropylidene oxindole (**2f**). Arrows indicate specific signals that allow attributing the configuration.

For alkylphenone derivatives, differences are also observed in the position of vinylic proton signals (signal shifts to higher field in the case of *Z*-isomers), although not so significant. At the same time, we did not see significant differences in the position of ortho-proton signals between *E*- and *Z*-isomers. In turn, the most characteristic for the considered group of compounds are H_4 proton signals: in all the analyzed cases, Δδ was more than 1 ppm, which visually looks like a jump of the doublet from the extreme right side of the aromatic group to the extreme left side during the transition from *E*- to *Z*-isomer.

It should be noted that the proton signal at the nitrogen atom can be significantly shifted in the presence of impurities and also depending on the sample concentration, which is confirmed by a large spread in the values of this parameter even for the same compound in the literature. Thus, despite the fact that, under equal conditions, there will be a significant difference in the position of the signal of this proton in the spectra of *E*- and *Z*-isomers, it can hardly be used to interpret the geometric structure. The values of the chemical shifts of proton signals of the studied compounds, which can be used for assignment to *E*- or *Z*-isomer, are given in Table S2.

Table S2. Chemical shifts of characteristic protons of model compounds in NMR spectra.

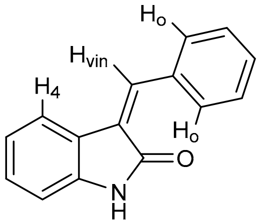

Compound	Isomer	H_4	H_{vin}	H_o
**2a**	*E*	7.64 (d)	7.86 (H, s)	7.68 (m)
	Z	7.51 (d)	7.55 (H, s)	8.27 (m)
**2b**	*E*	7.33 (d)	7.89 (H, s)	7.74 (dd)
	Z	7.57 (d)	7.85 (H, s)	8.37 (m)
**2c**	*E*	7.58 (d)	7.76 (H, s)	7.61 (d)
	Z	7.51 (d)	7.48 (H, s)	8.23 (d)
**2d**	*E*	6.14 (d)	2.81 (CH_3, s)	7.30 (m)
	Z	7.63 (d)	2.63 (CH_3, s)	7.34 (m)
**2e**	*E*	6.23 (d)	2.77 (CH_3, s)	7.26 (d)
	Z	7.62 (d)	2.62 (CH_3, s)	7.27 (d)
**2f**	*E*	6.02 (d)	3.31 (CH_2,q)	7.25 (m)
	Z	7.60 (d)	2.97 (CH_2,q)	7.28 (m)
**3c**	*E*	7.60 (d, overlapping)	7.81 (H, s)	7.60 (d)
	Z	7.55 (d)	7.51 (H, s)	8.23 (d)
**3d**	*E*	6.19 (d)	2.81 (CH_3, s)	7.30 (m)
	Z	7.70 (d)	2.67 (CH_3, s)	7.34 (m)
**3e**	*E*	6.27 (d)	2.77 (CH_3, s)	7.26 (d)
	Z	7.68 (d)	2.64 (CH_3, s)	7.27 (d)

Characterization of studied compounds

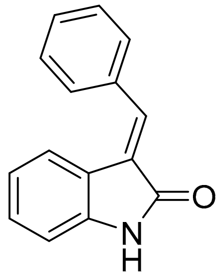

3-benzylideneindolin-2-one (**2a**)

E-isomer: yellow crystals, m.p. 171–172°C; ^{1}H NMR (400 MHz, $CDCl_3$) δ 9.36 (br s, 1H), 7.86 (s, 1H), 7.70–7.66 (m, 2H), 7.64 (d, *J* = 7.7 Hz, 1H), 7.51–7.43 (m, 3H), 7.22 (td, *J* = 7.7, 1.1 Hz, 1H), 6.96 (d, *J* = 7.8 Hz, 1H), 6.87 (td, *J* = 7.7, 1.0 Hz, 1H); ^{13}C NMR (101 MHz, $CDCl_3$) δ 170.81, 141.90, 137.74, 134.95, 130.05, 129.82, 129.48, 128.79, 127.82, 123.13, 121.97, 121.79, 110.56; MS (ESI) m/z: 222.2 $[M + H]^+$.

Z-isomer: deep yellow crystals, m.p. 178–179°C; ^{1}H NMR (400 MHz, $CDCl_3$) δ 8.85 (br s, 1H), 8.29–8.24 (m, 2H), 7.55 (s, 1H), 7.51 (d, *J* = 7.6 Hz, 1H), 7.48–7.40 (m, 3H), 7.21 (t, *J* = 7.6 Hz, 1H), 7.03 (t, *J* = 7.6 Hz, 1H), 6.86 (d, *J* = 7.7 Hz, 1H); ^{13}C NMR (101 MHz, $CDCl_3$) δ 168.29, 139.98, 137.74, 133.88, 132.08, 130.64, 129.04, 128.40, 126.56, 125.35, 121.88, 119.38, 109.87; MS (ESI) m/z: 222.2 $[M + H]^+$.

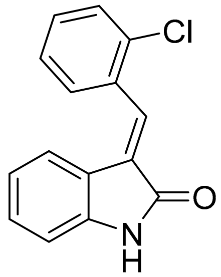

3-(2-chlorobenzylidene)indolin-2-one (**2b**)

E-isomer: yellow crystals, m.p. 172–173°C; ^{1}H NMR (400 MHz, $CDCl_3$) δ 9.38 (br s, 1H), 7.89 (s, 1H), 7.74 (dd, *J* = 7.3, 1.8 Hz, 1H), 7.52 (dd, *J* = 7.8, 1.4 Hz, 1H), 7.39 (td, *J* = 7.5, 1.8 Hz, 1H), 7.35 (td, *J* = 7.2, 1.4 Hz, 1H), 7.33 (d, *J* = 7.7 Hz, 1H), 7.22 (td, *J* = 7.7, 0.7 Hz, 1H), 6.96 (d, *J* = 7.8 Hz, 1H), 6.83 (td, *J* = 7.6, 0.6 Hz, 1H); ^{13}C NMR (101 MHz, $CDCl_3$) δ 170.27, 142.14, 134.60, 134.00, 133.64, 130.91, 130.44, 130.32, 130.16, 129.35, 126.76, 123.30, 122.02, 121.45, 110.68; MS (ESI) m/z: 256.1 $[M + H]^+$.

Z-isomer: deep yellow crystals, m.p. 179–180°C; ^{1}H NMR (400 MHz, $CDCl_3$) δ 8.39–8.35 (m, 1H), 7.85 (s, 1H), 7.57 (d, *J* = 7.6 Hz, 1H), 7.47–7.42 (m, 1H), 7.36–7.30 (m, 2H), 7.25 (t, *J* = 7.7 Hz, 1H), 7.06 (t, *J* = 7.6 Hz, 1H), 6.85 (d, *J* = 7.8 Hz, 1H); ^{13}C NMR (101 MHz,

$CDCl_3$) δ 167.73, 140.15, 135.06, 133.22, 132.59, 131.75, 131.34, 129.73, 129.44, 127.87, 126.36, 124.71, 122.23, 120.25, 109.91; MS (ESI) m/z: 256.1 $[M + H]^+$.

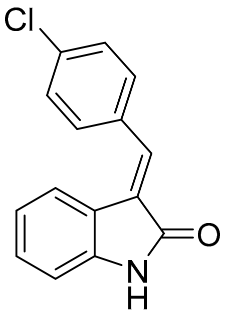

3-(4-chlorobenzylidene)indolin-2-one (**2c**)

E-isomer: yellow crystals, m.p. 188–190°C; ^1H NMR (400 MHz, $CDCl_3$) δ 8.95 (br s, 1H), 7.76 (s, 1H), 7.61 (d, *J* = 8.4 Hz, 2H), 7.58 (d, *J* = 7.6 Hz, 1H), 7.45 (d, *J* = 8.5 Hz, 2H), 7.23 (td, *J* = 7.7, 1.0 Hz, 1H), 6.93 (d, *J* = 7.7 Hz, 1H), 6.88 (td, *J* = 7.7, 0.9 Hz, 1H); ^{13}C NMR (101 MHz, $CDCl_3$) δ 170.35, 141.89, 136.12, 135.73, 133.37, 130.80, 130.35, 129.15, 128.19, 123.13, 122.12, 121.54, 110.60; MS (ESI) m/z: 256.2 $[M + H]^+$.

Z-isomer: orange crystals, m.p. 205–207°C; ^1H NMR (400 MHz, $CDCl_3$) δ 8.23 (d, *J* = 8.5 Hz, 2H), 7.72 (s, 1H), 7.51 (d, *J* = 7.5 Hz, 1H), 7.48 (s, 1H), 7.41 (d, *J* = 8.6 Hz, 2H), 7.24 (td, *J* = 7.7, 1.1 Hz, 1H), 7.05 (td, *J* = 7.6, 0.9 Hz, 1H), 6.84 (d, *J* = 7.7 Hz, 1H); ^{13}C NMR (101 MHz, $CDCl_3$) δ 167.72, 139.72, 136.65, 136.23, 133.43, 132.33, 129.35, 128.72, 126.73, 125.21, 122.14, 119.58, 109.75; MS (ESI) m/z: 256.2 $[M + H]^+$.

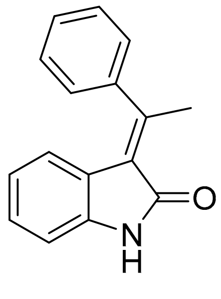

3-(1-phenylethylidene)indolin-2-one (**2d**)

E-isomer: yellow crystals, m.p. 192–194°C; ^1H NMR (400 MHz, $CDCl_3$) δ 8.43 (br s, 1H), 7.52–7.42 (m, 3H), 7.32–7.28 (m, 2H), 7.07 (t, *J* = 7.7 Hz, 1H), 6.82 (d, *J* = 7.7 Hz, 1H), 6.61 (t, *J* = 7.7 Hz, 1H), 6.14 (d, *J* = 7.8 Hz, 1H), 2.81 (s, 3H); ^{13}C NMR (101 MHz, $CDCl_3$) δ 170.11, 155.70, 143.05, 139.66, 129.32, 128.53, 128.25, 126.57, 123.83, 123.56, 123.29, 121.44, 109.39, 22.99; MS (ESI) m/z: 236.2 $[M + H]^+$.

Z-isomer: yellow crystals, m.p. 180–181°C; ^1H NMR (400 MHz, $CDCl_3$) δ 7.98 (br s, 1H), 7.63 (d, *J* = 7.7 Hz, 1H), 7.44–7.36 (m, 3H), 7.36–7.31 (m, 2H), 7.22 (t, *J* = 7.7 Hz, 1H), 7.05 (t, *J* = 7.7 Hz, 1H), 6.77 (d, *J* = 7.7 Hz, 1H), 2.63 (s, 3H); ^{13}C NMR (101 MHz, $CDCl_3$) δ

167.79, 153.70, 142.40, 140.68, 128.58, 128.29, 128.18, 127.68, 124.48, 124.38, 123.98, 121.81, 109.61, 25.78; MS (ESI) m/z: 236.2 $[M + H]^+$.

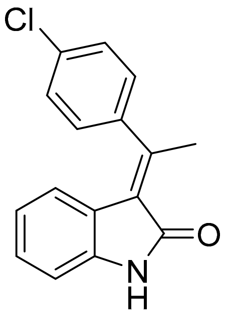

3-(1-(4-chlorophenyl)ethylidene)indolin-2-one (**2e**)

E-isomer: yellow crystals, m.p. 232–233°C; ^1H NMR (400 MHz, $CDCl_3$) δ 8.29 (br s, 1H), 7.47 (d, *J* = 8.4 Hz, 2H), 7.26 (d, *J* = 8.4 Hz, 2H), 7.10 (t, *J* = 7.7 Hz, 1H), 6.82 (d, *J* = 7.8 Hz, 1H), 6.67 (t, *J* = 7.7 Hz, 1H), 6.23 (d, *J* = 7.8 Hz, 1H), 2.77 (s, 3H); ^{13}C NMR (101 MHz, $CDCl_3$) δ 169.78, 154.17, 141.29, 139.63, 134.61, 129.67, 128.60, 128.28, 124.14, 123.24, 123.21, 121.68, 109.60, 22.91; MS (ESI) m/z: 270.2 $[M + H]^+$.

Z-isomer: yellow crystals, m.p. 199–200°C; ^1H NMR (400 MHz, $CDCl_3$) δ 8.11 (br s, 1H), 7.62 (d, *J* = 7.7 Hz, 1H), 7.37 (d, *J* = 8.6 Hz, 2H), 7.27 (d, *J* = 8.6 Hz, 2H), 7.25 (td, *J* = 7.7, 0.9 Hz, 1H), 7.07 (td, *J* = 7.7, 0.9 Hz, 1H), 6.83 (d, *J* = 7.7 Hz, 1H), 2.62 (s, 3H); ^{13}C NMR (101 MHz, $CDCl_3$) δ 167.75, 152.64, 140.53, 140.52, 134.37, 129.27, 128.92, 128.48, 124.49, 124.29, 124.19, 122.17, 109.90, 25.69; MS (ESI) m/z: 270.2 $[M + H]^+$.

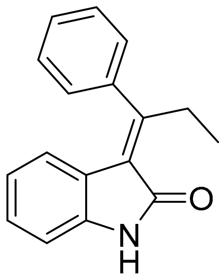

3-(1-Phenylpropylidene)indolin-2-one (**2f**)

E-isomer: light yellow crystals, m.p. 148–149°C; ^1H NMR (400 MHz, $CDCl_3$) δ 8.42 (br s, 1H), 7.52–7.43 (m, 3H), 7.27–7.23 (m, 2H), 7.07 (td, *J* = 7.7, 1.2 Hz, 1H), 6.82 (d, *J* = 7.8 Hz, 1H), 6.61 (td, *J* = 7.7, 1.1 Hz, 1H), 6.02 (d, *J* = 7.8 Hz, 1H), 3.31 (q, *J* = 7.5 Hz, 2H), 1.12 (t, *J* = 7.5 Hz, 3H); ^{13}C NMR (101 MHz, $CDCl_3$) δ 169.68, 162.34, 141.31, 139.56, 129.25, 128.52, 128.29, 126.96, 123.62, 123.39, 123.17, 121.66, 109.51, 28.39, 12.16; MS (ESI) m/z: 250.2 $[M + H]^+$.

Z-isomer: light yellow crystals, m.p. 139–140°C; ^1H NMR (400 MHz, $CDCl_3$) δ 8.63 (br s, 1H), 7.60 (d, *J* = 7.7 Hz, 1H), 7.46–7.36 (m, 3H), 7.31–7.25 (m, 2H), 7.21 (t, *J* = 7.6 Hz, 1H),

7.05 (t, J = 7.6 Hz, 1H), 6.73 (d, J = 7.7 Hz, 1H), 2.97 (q, J = 7.5 Hz, 2H), 1.21 (t, J = 7.5 Hz, 3H); ^{13}C NMR (101 MHz, $CDCl_3$) δ 168.41, 160.06, 141.23, 140.92, 128.63, 128.16, 128.10, 127.70, 123.82, 123.50, 123.44, 121.91, 110.01, 31.28, 11.30; MS (ESI) m/z: 250.2 $[M + H]^+$.

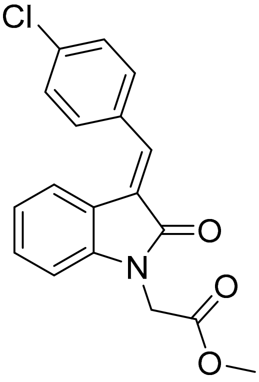

Methyl 2-(3-(4-chlorobenzylidene)-2-oxoindolin-1-yl)acetate (**3c**)

E-isomer: yellow crystals, m.p. 165–166°C; ^{1}H NMR (400 MHz, $CDCl_3$) δ 7.81 (s, 1H), 7.60 (d, J = 8.4 Hz, 2H), 7.60 (d, J = 7.7 Hz, 1H), 7.45 (d, J = 8.5 Hz, 2H), 7.26 (td, J = 7.7, 1.0 Hz, 1H), 6.92 (td, J = 7.7, 0.9 Hz, 1H), 6.73 (d, J = 7.7 Hz, 1H), 4.56 (s, 2H), 3.77 (s, 3H); ^{13}C NMR (101 MHz, $CDCl_3$) δ 168.37, 168.32, 143.09, 136.46, 135.73, 133.38, 130.78, 130.26, 129.17, 127.26, 123.09, 122.47, 121.06, 108.51, 52.78, 41.41; MS (ESI) m/z: 328.2 $[M + H]^+$.

Z-isomer: yellow crystals, m.p. 180–181°C; ^{1}H NMR (400 MHz, $CDCl_3$) δ 8.23 (d, J = 8.4 Hz, 2H), 7.55 (d, J = 7.5 Hz, 1H), 7.51 (s, 1H), 7.40 (d, J = 8.5 Hz, 2H), 7.28 (t, J = 7.6 Hz, 1H), 7.09 (t, J = 7.5 Hz, 1H), 6.72 (d, J = 7.8 Hz, 1H), 4.54 (s, 2H), 3.76 (s, 3H); ^{13}C NMR (101 MHz, $CDCl_3$) δ 168.46, 166.06, 141.16, 136.69, 136.39, 133.44, 132.29, 129.33, 128.67, 125.94, 124.26, 122.55, 119.47, 108.14, 52.75, 41.26; MS (ESI) m/z: 328.2 $[M + H]^+$.

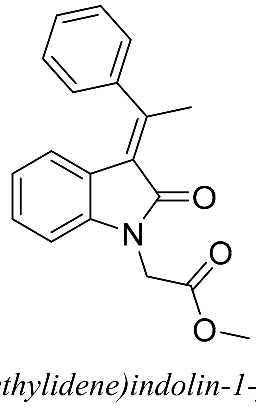

Methyl 2-(2-oxo-3-(1-phenylethylidene)indolin-1-yl)acetate (**3d**)

E-isomer: yellow crystals, m.p. 192–194°C; ^{1}H NMR (400 MHz, $CDCl_3$) δ 7.51–7.44 (m, 3H), 7.32–7.28 (m, 2H), 7.10 (td, J = 7.7, 1.1 Hz, 1H), 6.65 (dt, J = 7.8, 1.0 Hz, 1H), 6.65 (dd, J = 7.8, 1.1 Hz, 1H), 6.19 (dd, J = 8.0, 0.9 Hz, 1H), 4.56 (s, 2H), 3.77 (s, 3H), 2.81 (s, 3H); ^{13}C NMR (101 MHz, $CDCl_3$) δ 168.62, 167.95, 155.90, 142.85, 140.89, 129.24, 128.48, 128.17,

126.46, 123.11, 122.91, 122.70, 121.83, 107.50, 52.59, 40.94, 23.10; MS (ESI) m/z: 308.2 $[M + H]^+$.

Z-isomer: yellow crystals, m.p. 126–127°C; ^{1}H NMR (400 MHz, $CDCl_3$) δ 7.70 (d, *J* = 7.7 Hz, 1H), 7.45–7.37 (m, 3H), 7.36–7.32 (m, 2H), 7.28 (t, *J* = 7.8 Hz, 1H), 7.11 (t, *J* = 7.7 Hz, 1H), 6.72 (d, *J* = 7.8 Hz, 1H), 4.44 (s, 2H), 3.71 (s, 3H), 2.67 (s, 3H); ^{13}C NMR (101 MHz, $CDCl_3$) δ 168.59, 166.12, 154.26, 142.40, 141.98, 128.59, 128.28, 128.19, 127.45, 124.31, 123.66, 123.19, 122.32, 107.94, 52.56, 40.88, 25.73; MS (ESI) m/z: 308.2 $[M + H]^+$.

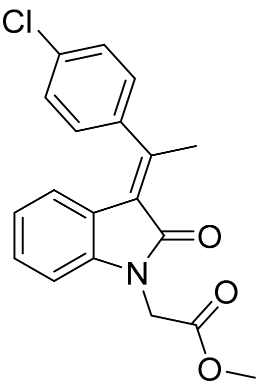

Methyl 2-(3-(1-(4-chlorophenyl)ethylidene)-2-oxoindolin-1-yl)acetate (**3e**)

E-isomer: light yellow crystals, m.p. 126–127°C; ^{1}H NMR (400 MHz, $CDCl_3$) δ 7.47 (d, *J* = 8.5 Hz, 2H), 7.26 (d, *J* = 8.5 Hz, 2H), 7.13 (td, *J* = 7.8, 0.8 Hz, 1H), 6.70 (td, *J* = 7.7, 0.9 Hz, 1H), 6.65 (d, *J* = 7.8 Hz, 1H), 6.27 (d, *J* = 7.7 Hz, 1H), 4.55 (s, 2H), 3.77 (s, 3H), 2.77 (s, 3H); ^{13}C NMR (101 MHz, $CDCl_3$) δ 168.62, 167.87, 154.15, 141.25, 141.09, 134.58, 129.65, 128.57, 128.26, 123.41, 123.15, 122.48, 122.04, 107.73, 52.72, 41.04, 23.06; MS (ESI) m/z: 342.2 $[M + H]^+$.

Z-isomer: deep yellow crystals, m.p. 145–146°C; ^{1}H NMR (400 MHz, $CDCl_3$) δ 7.68 (d, *J* = 7.7 Hz, 1H), 7.38 (d, *J* = 8.4 Hz, 2H), 7.29 (t, *J* = 7.6 Hz, 1H), 7.27 (d, *J* = 8.4 Hz, 2H), 7.11 (t, *J* = 7.7 Hz, 1H), 6.72 (d, *J* = 7.8 Hz, 1H), 4.43 (s, 2H), 3.71 (s, 3H), 2.64 (s, 3H); 168.50, 166.10, 152.49, 142.04, 140.66, 134.31, 129.07, 128.87, 128.48, 124.42, 123.65, 123.46, 122.47, 108.05, 52.63, 40.92, 25.54; MS (ESI) m/z: 342.2 $[M + H]^+$.

^1H and ^{13}C NMR spectra

Compound *E*-**2a**

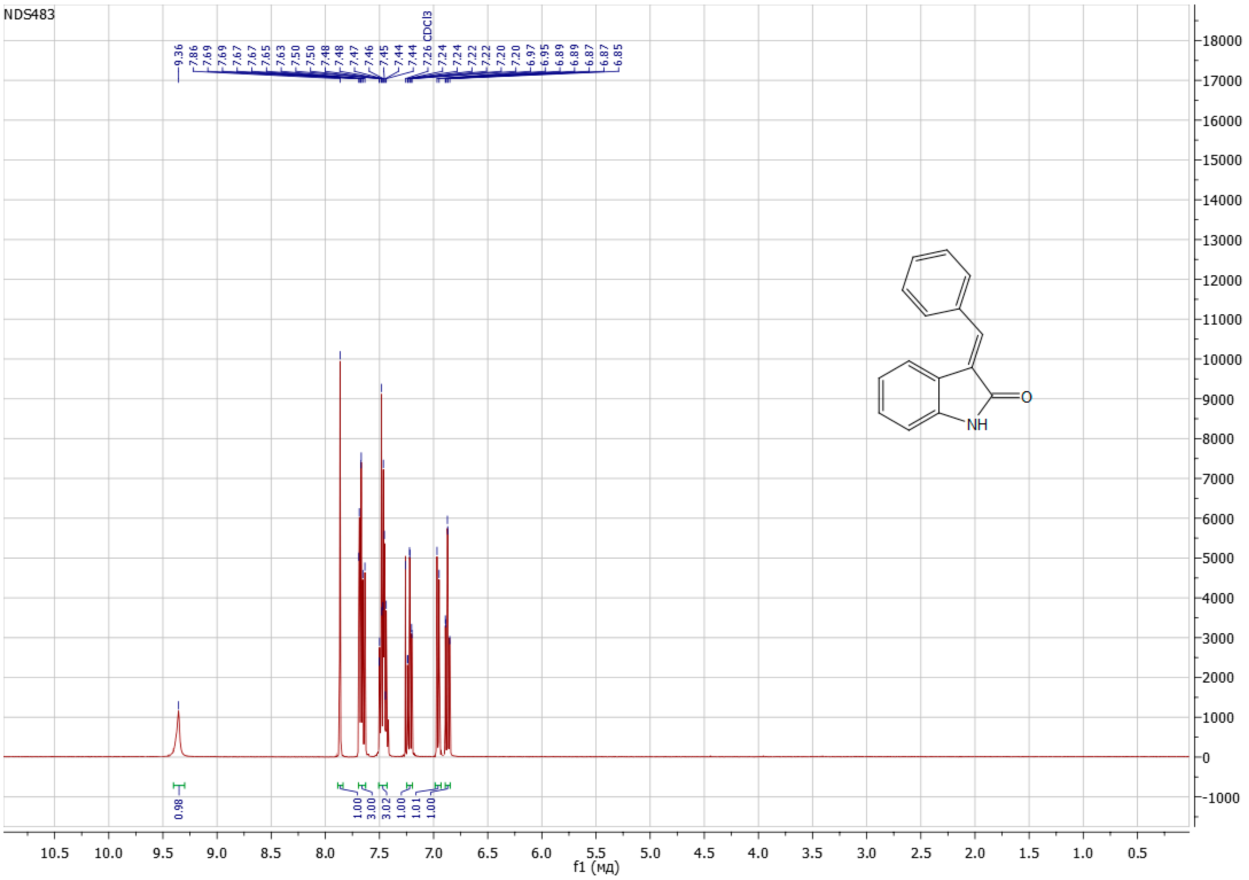

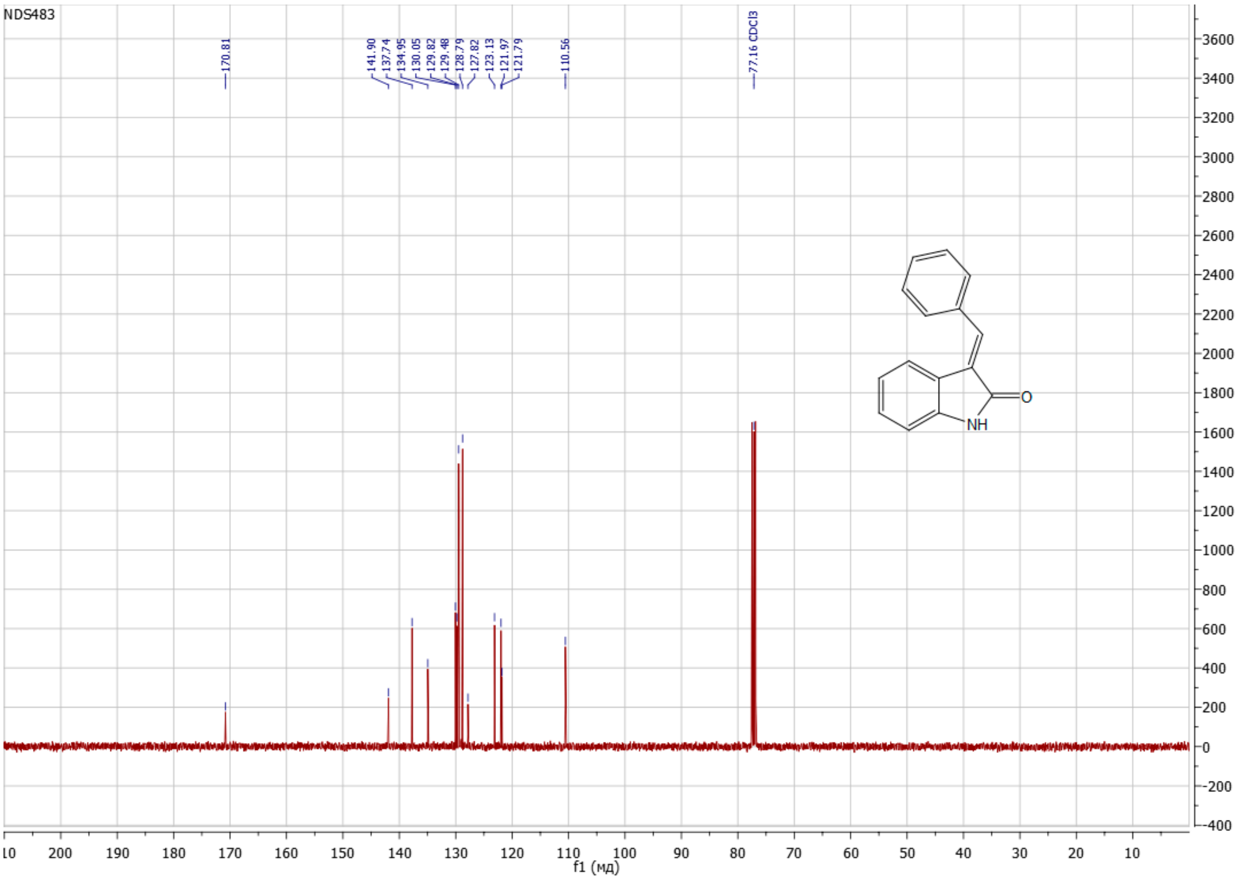

Compound *Z*-**2a**

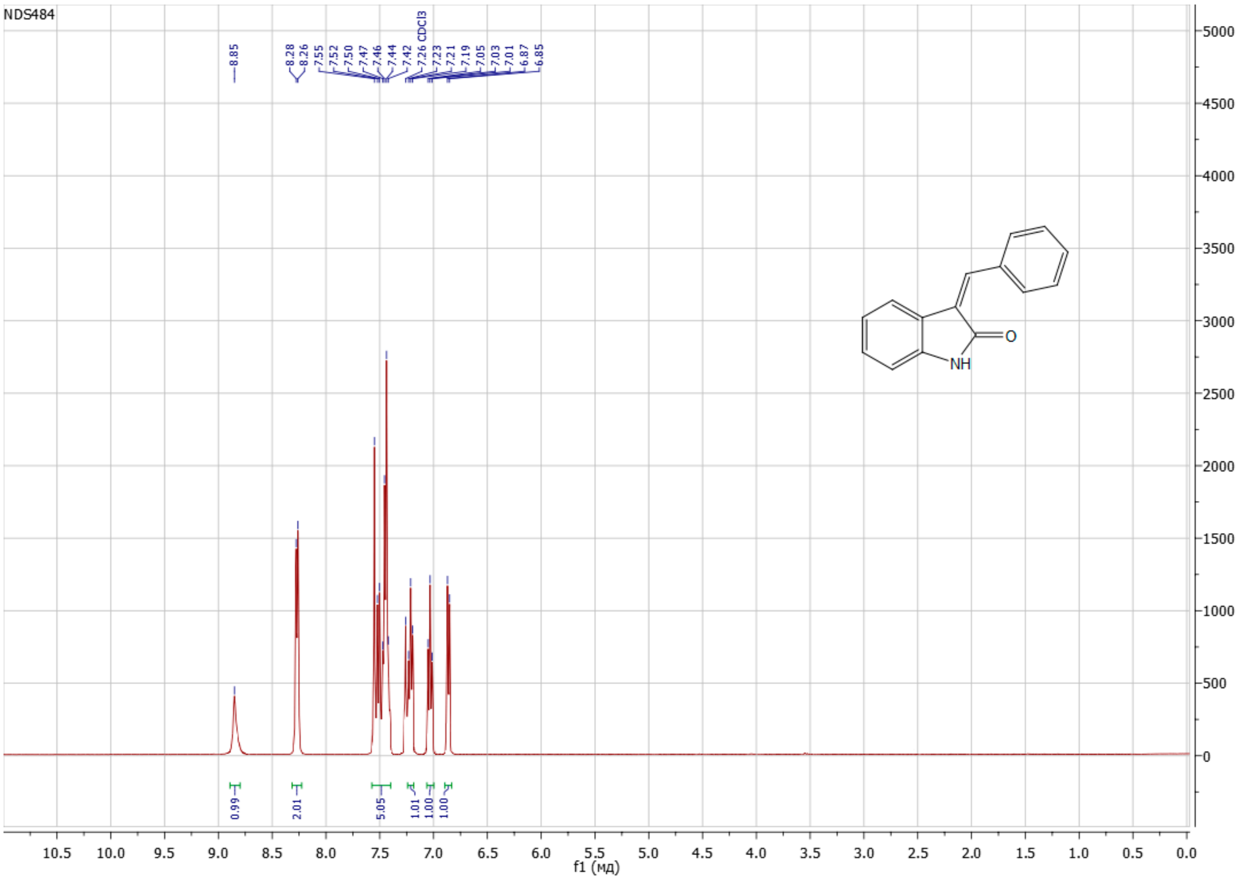

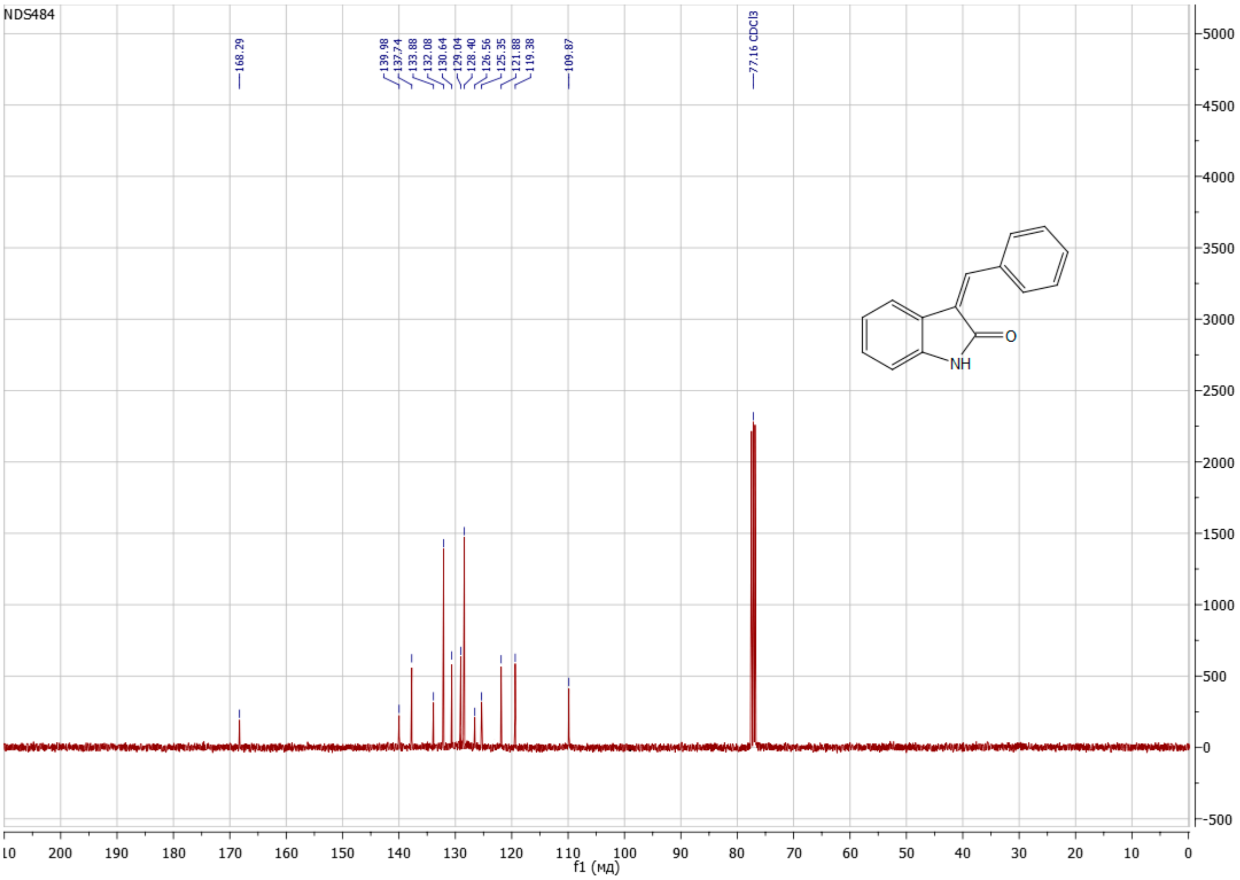

Compound *E*-**2b**

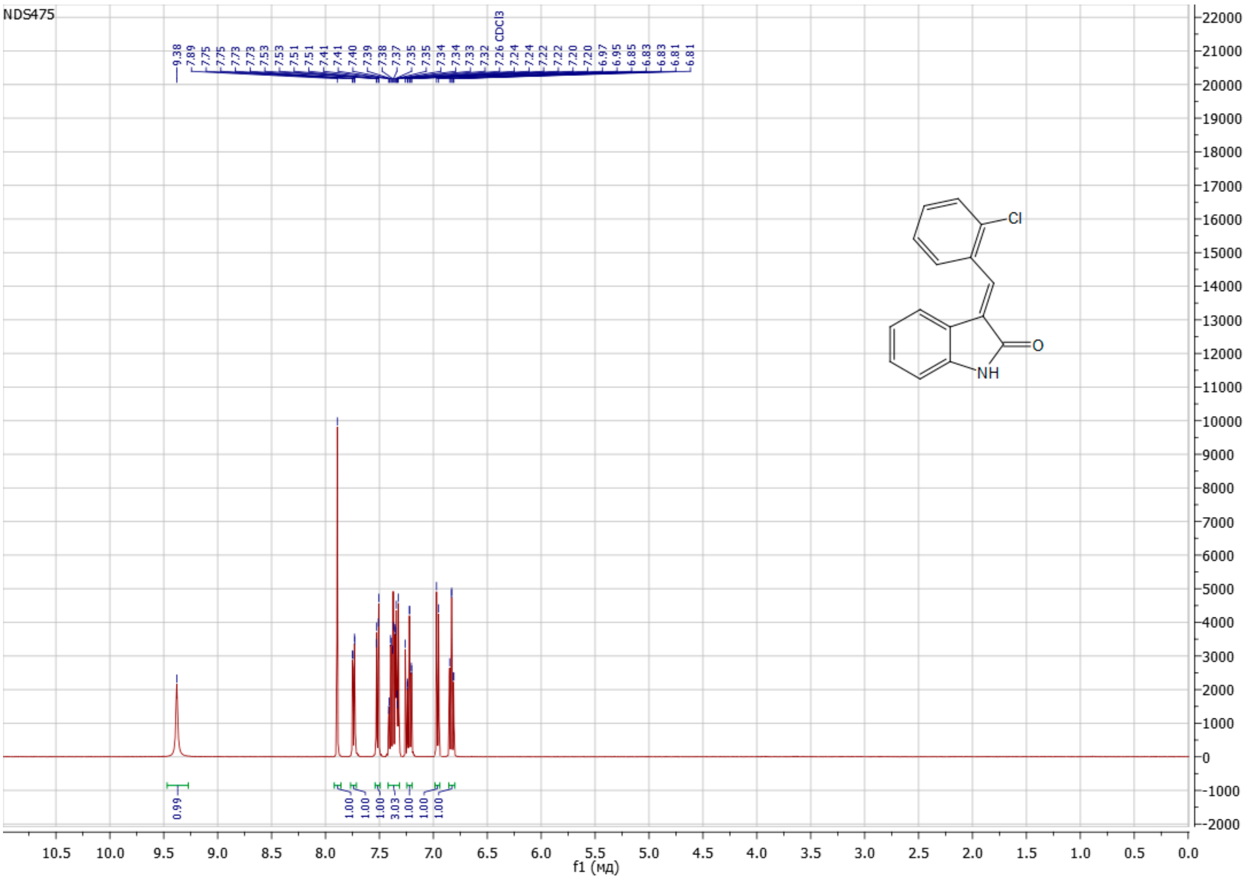

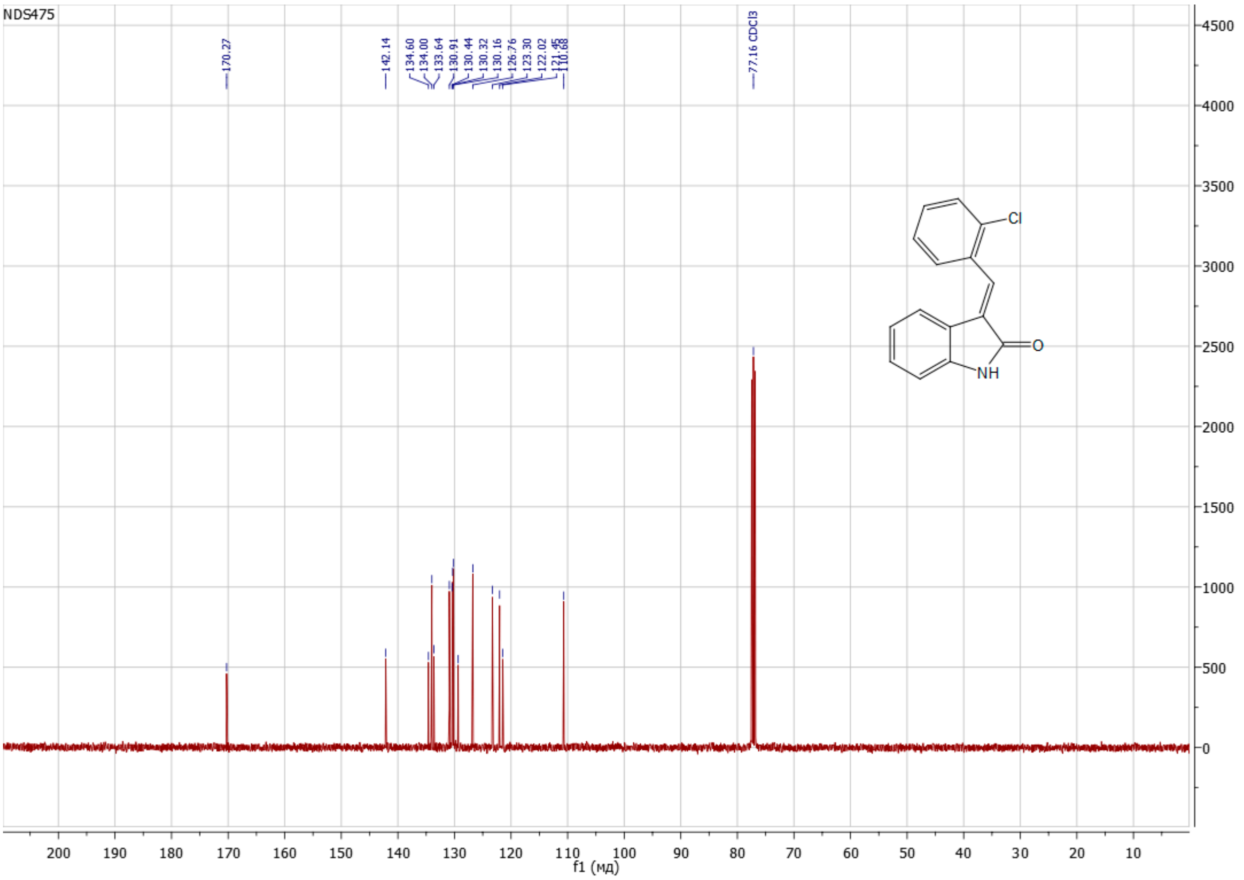

Compound *Z*-**2b**

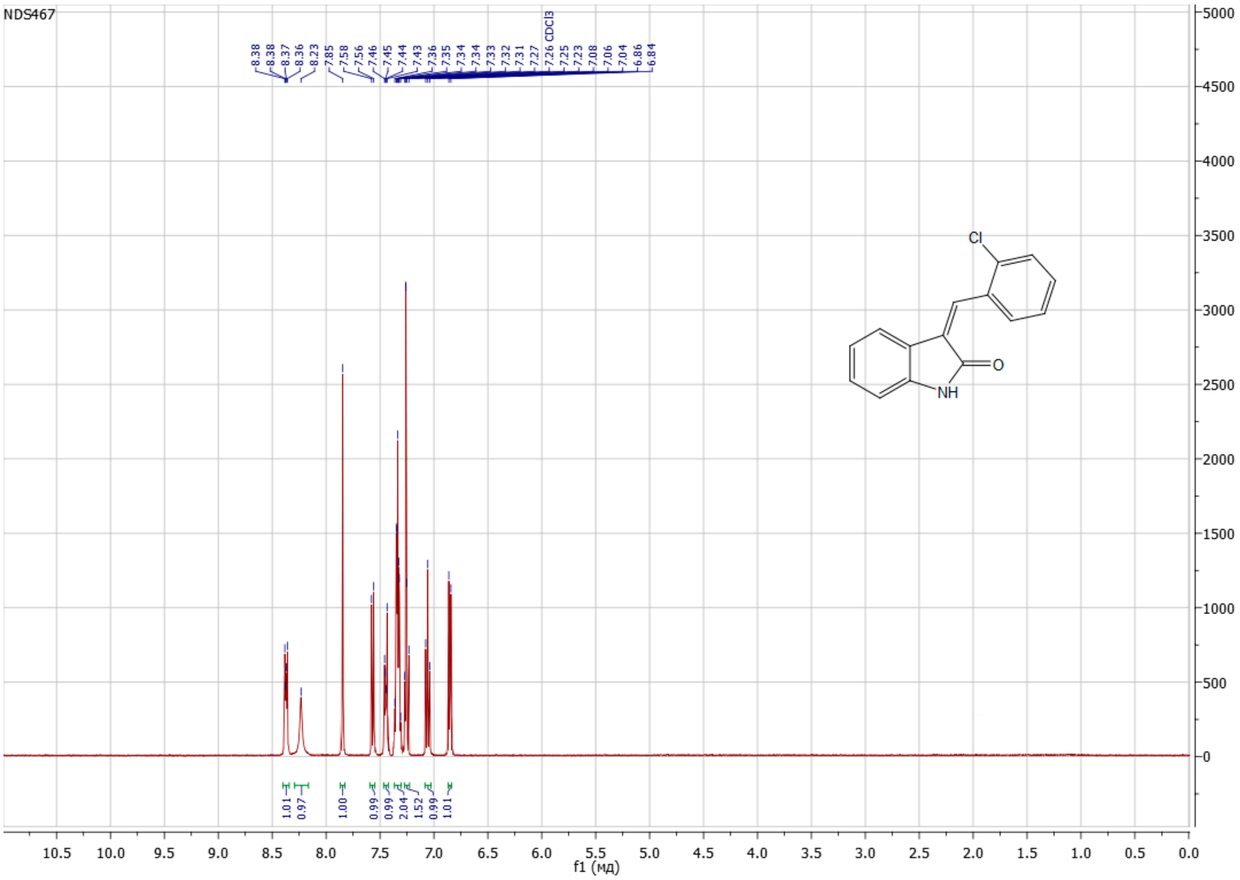

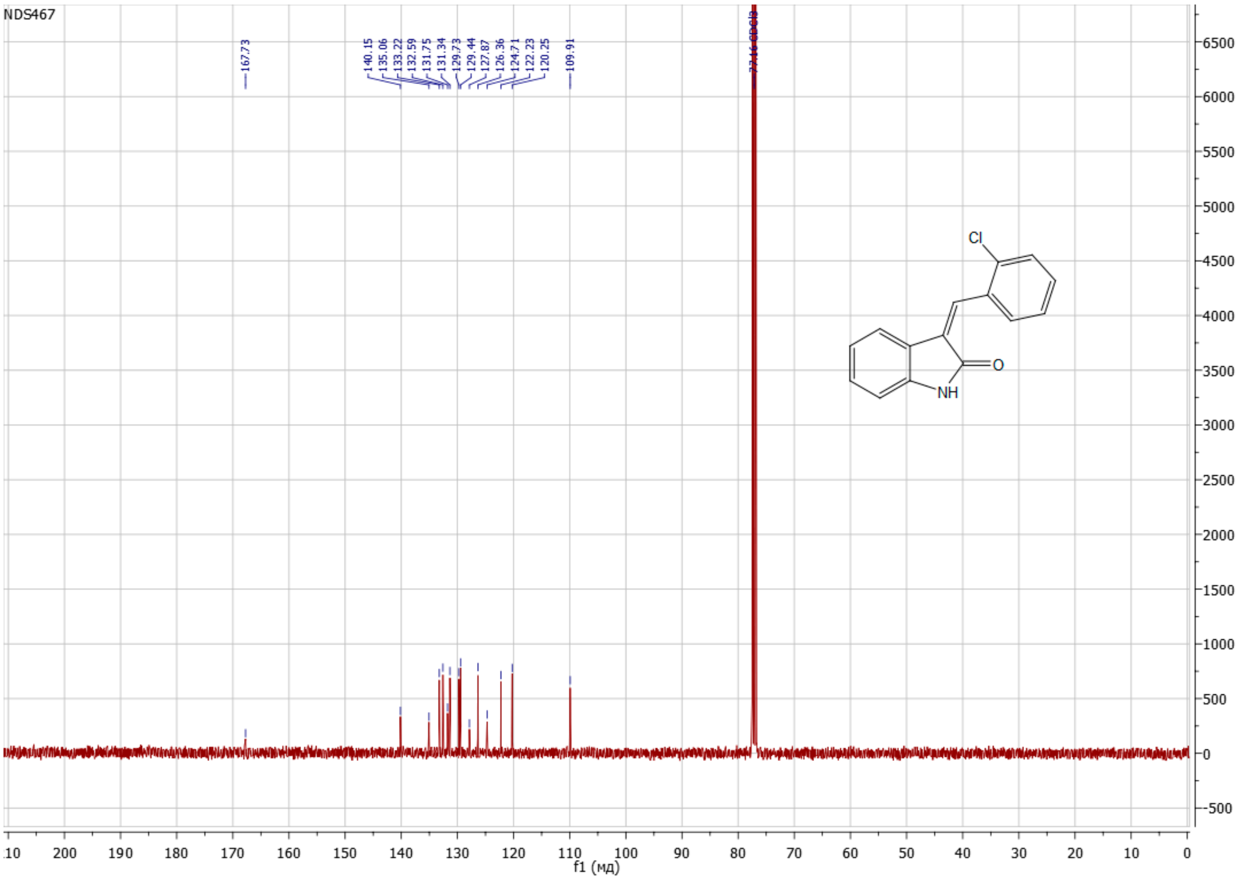

Compound *E*-**2c**

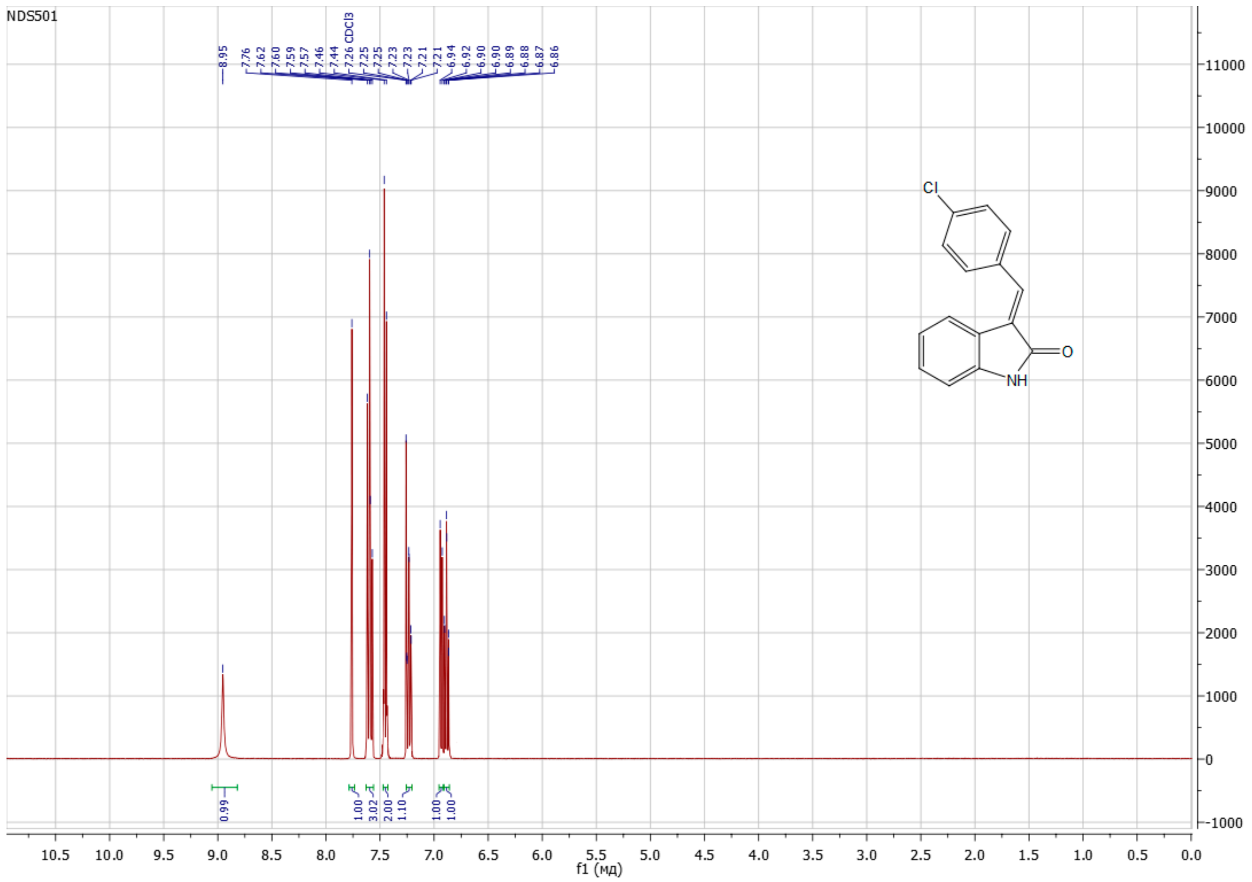

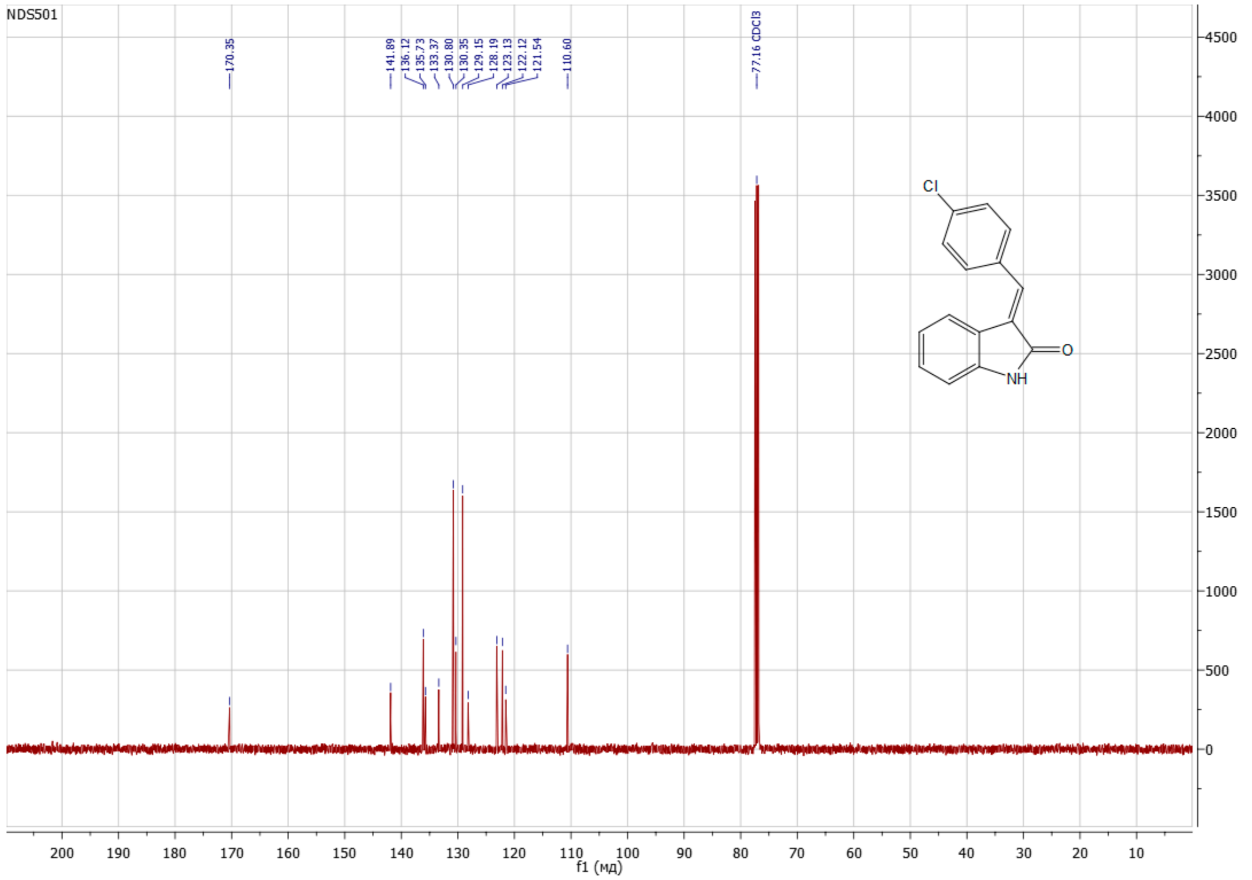

Compound *Z*-**2c**

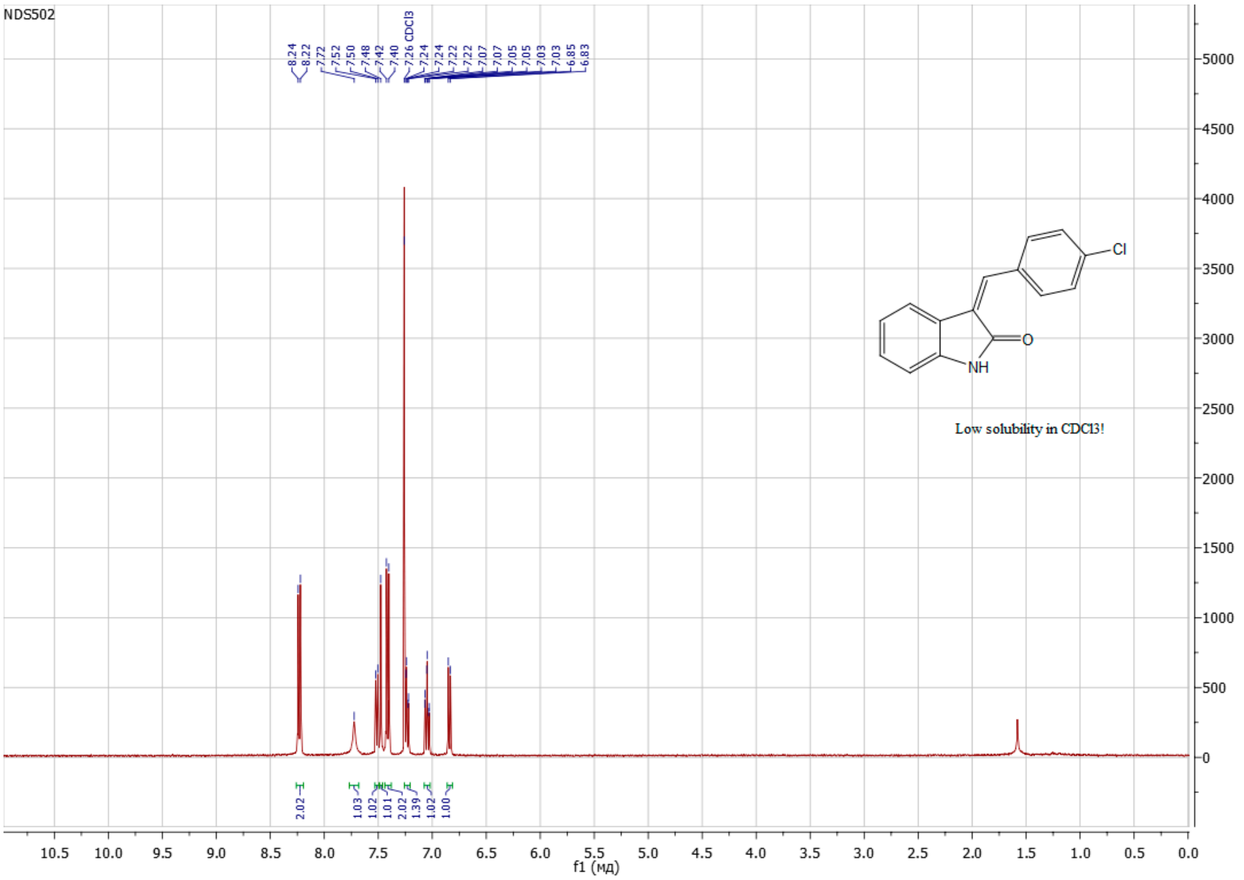

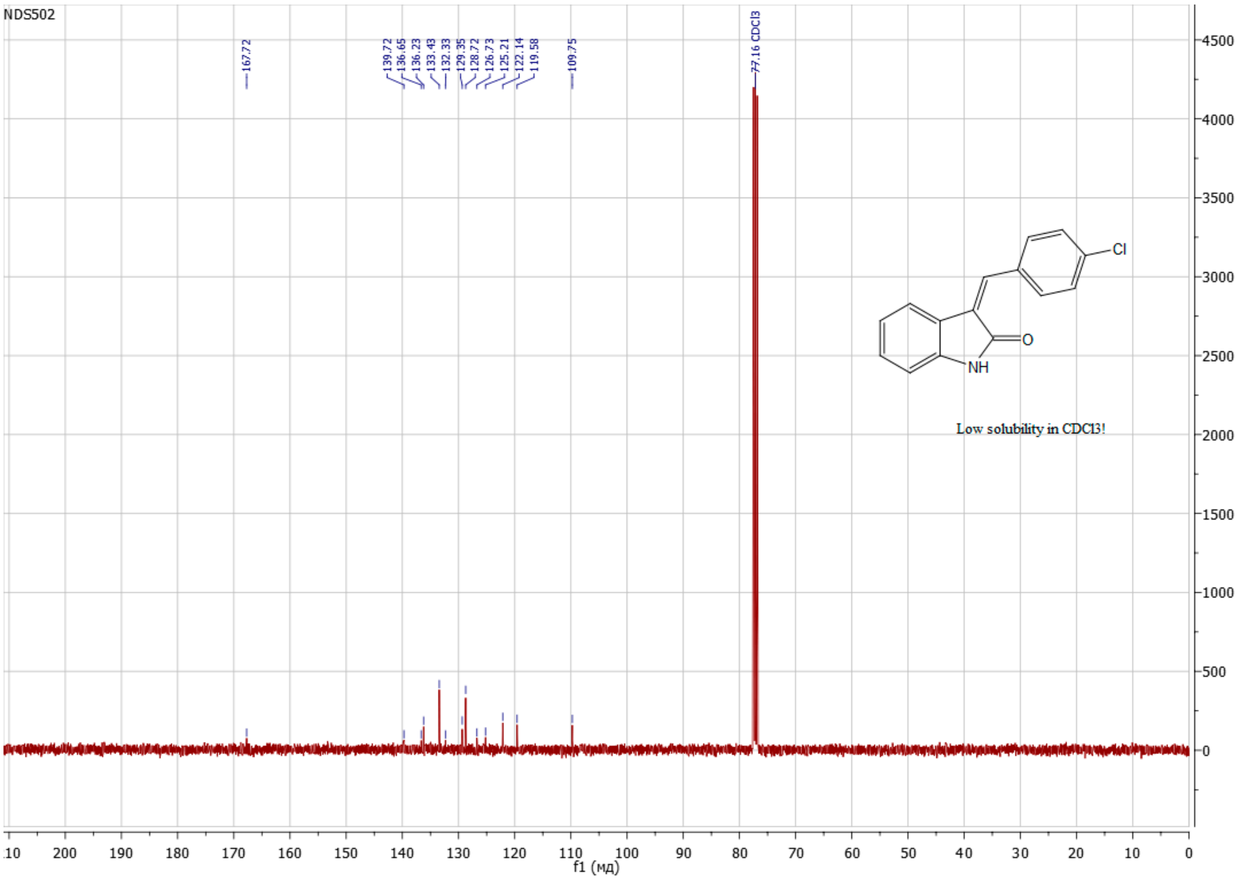

Compound *E*-**2d**

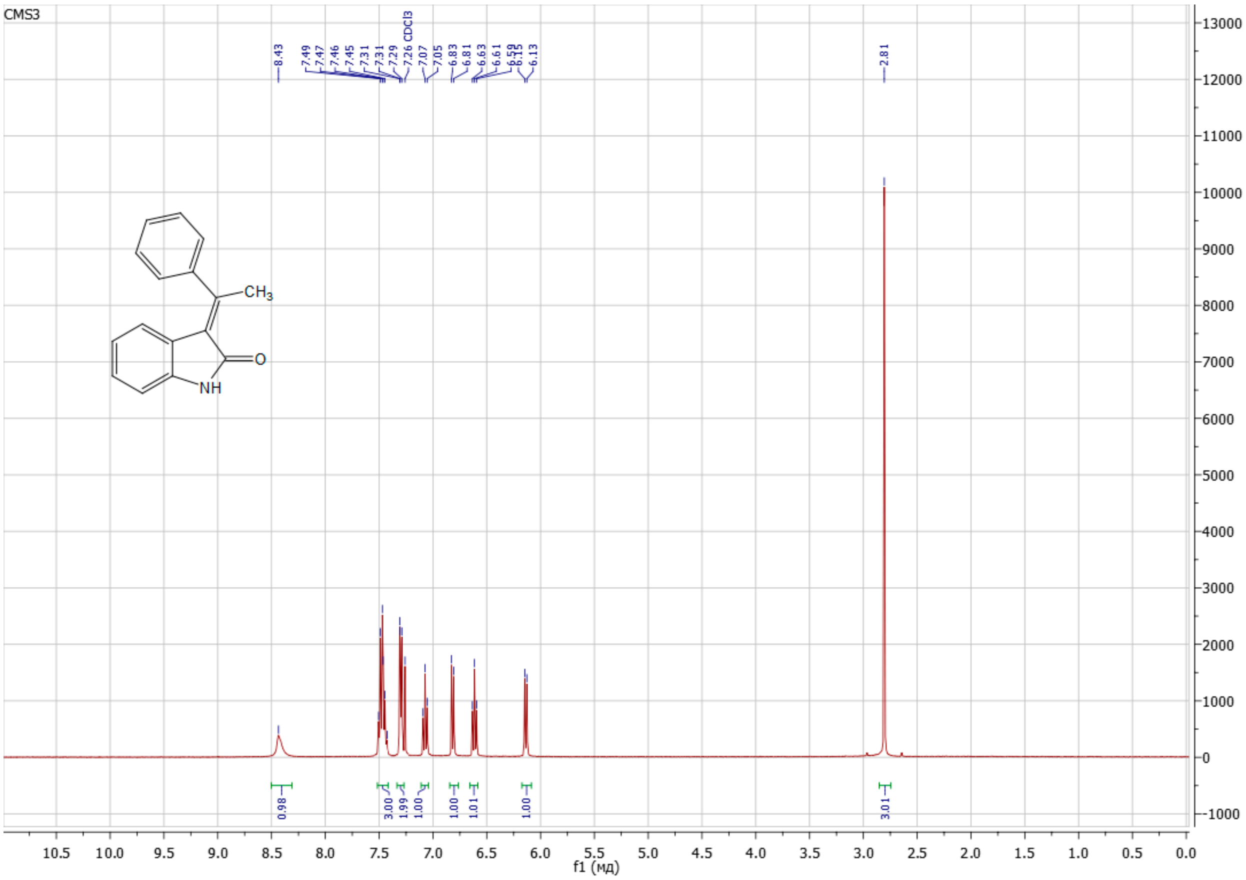

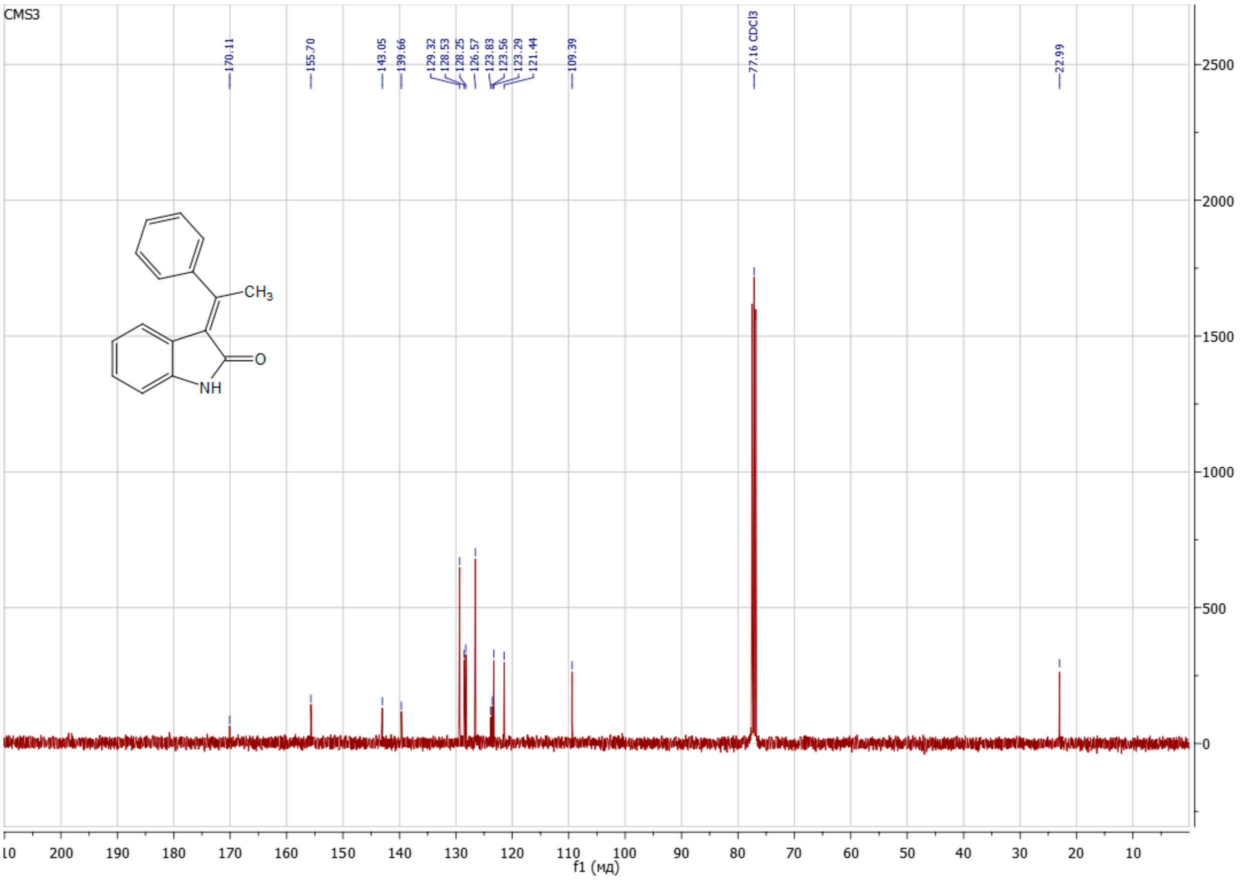

Compound *Z*-**2d**

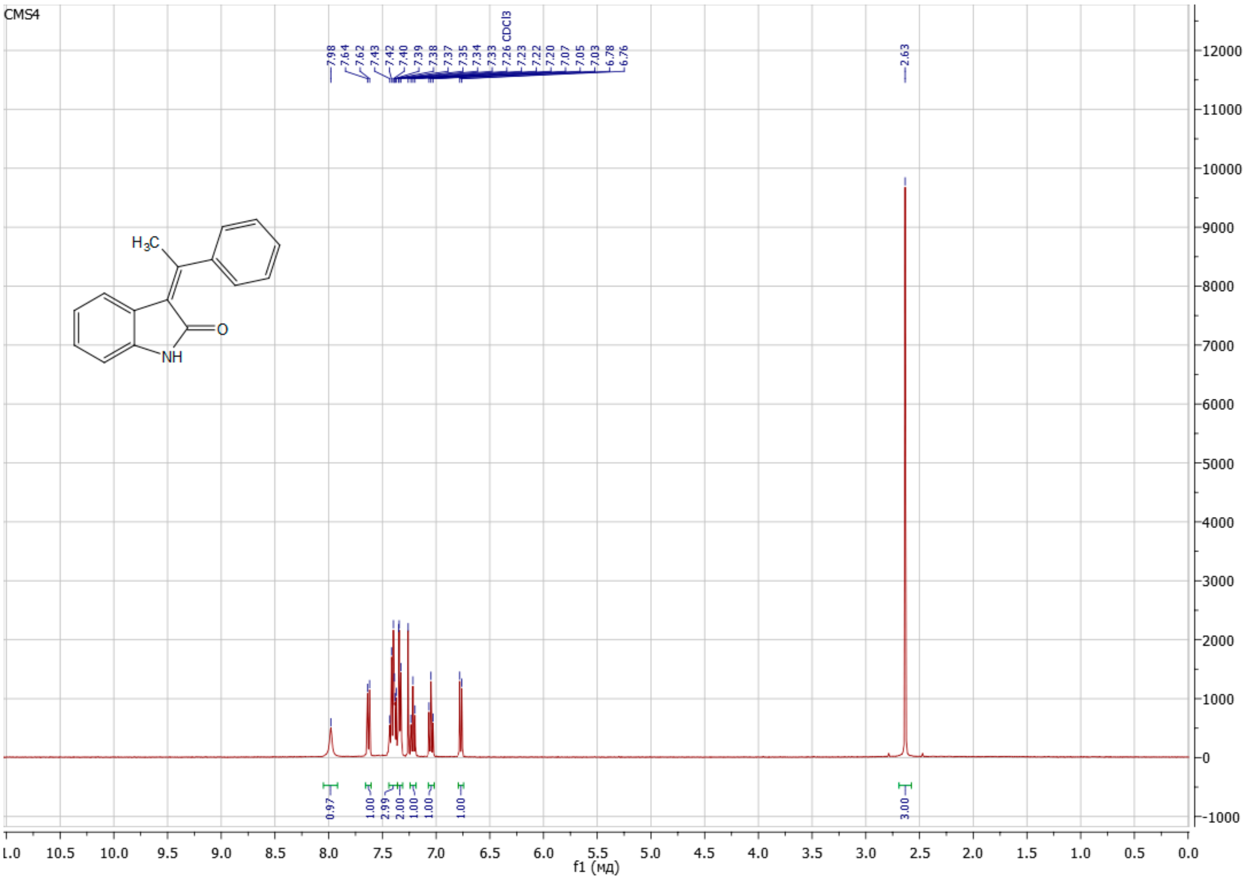

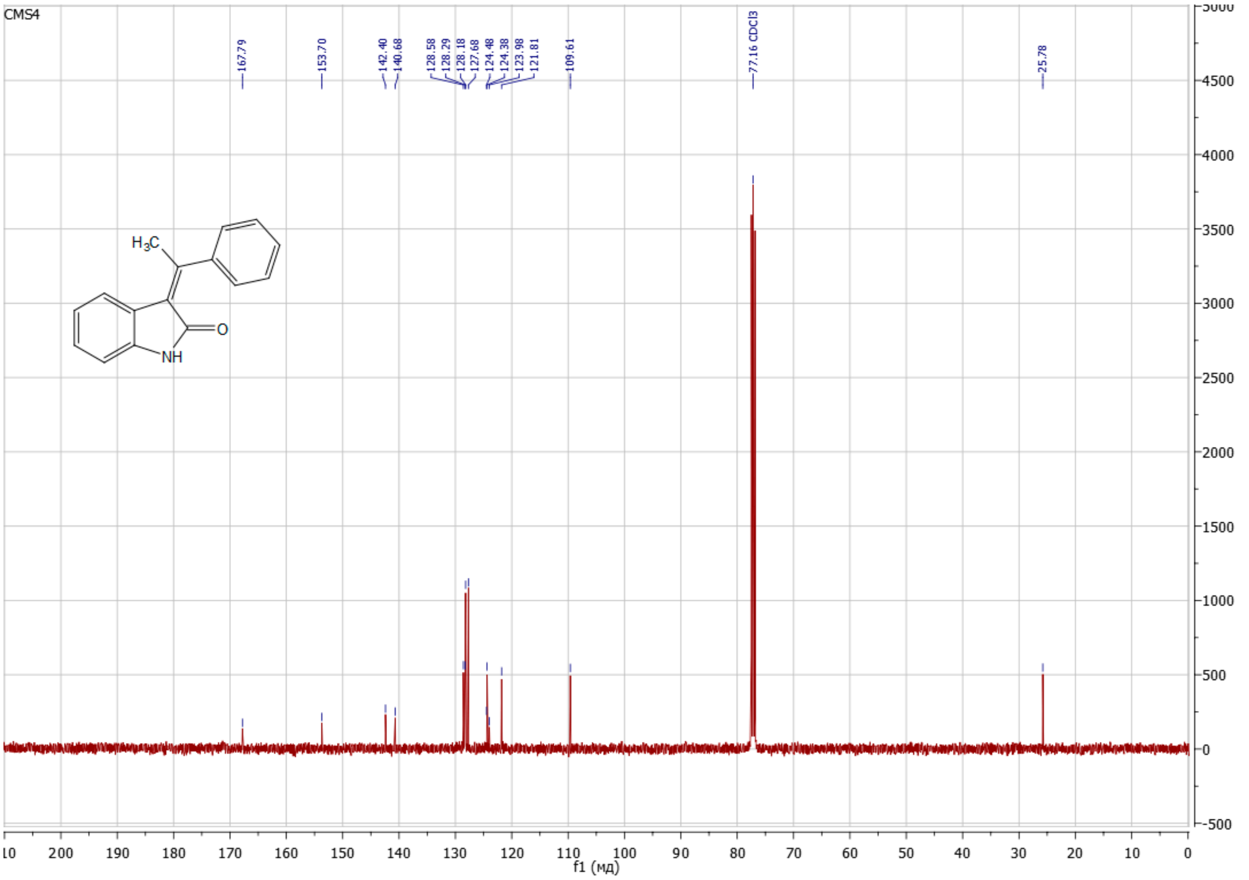

Compound *E*-**2e**

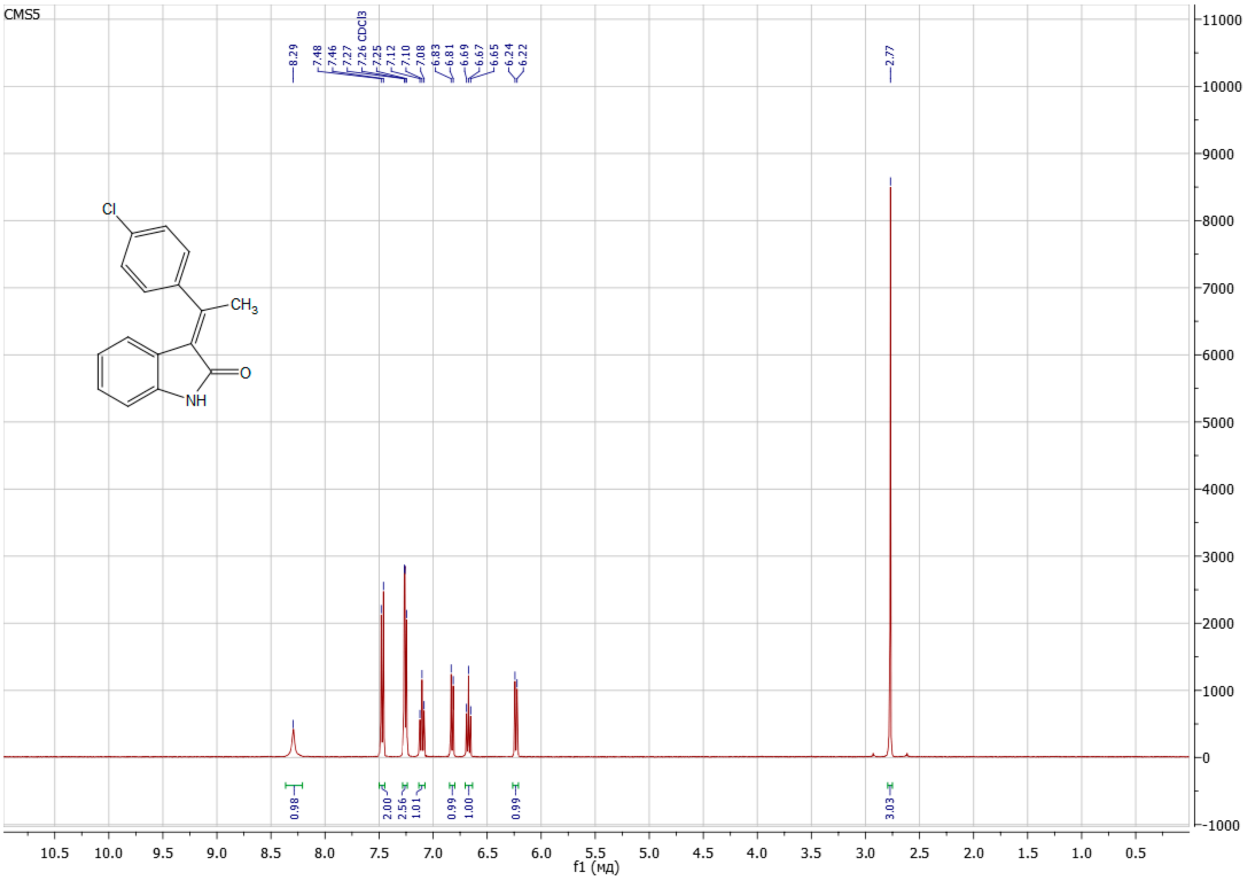

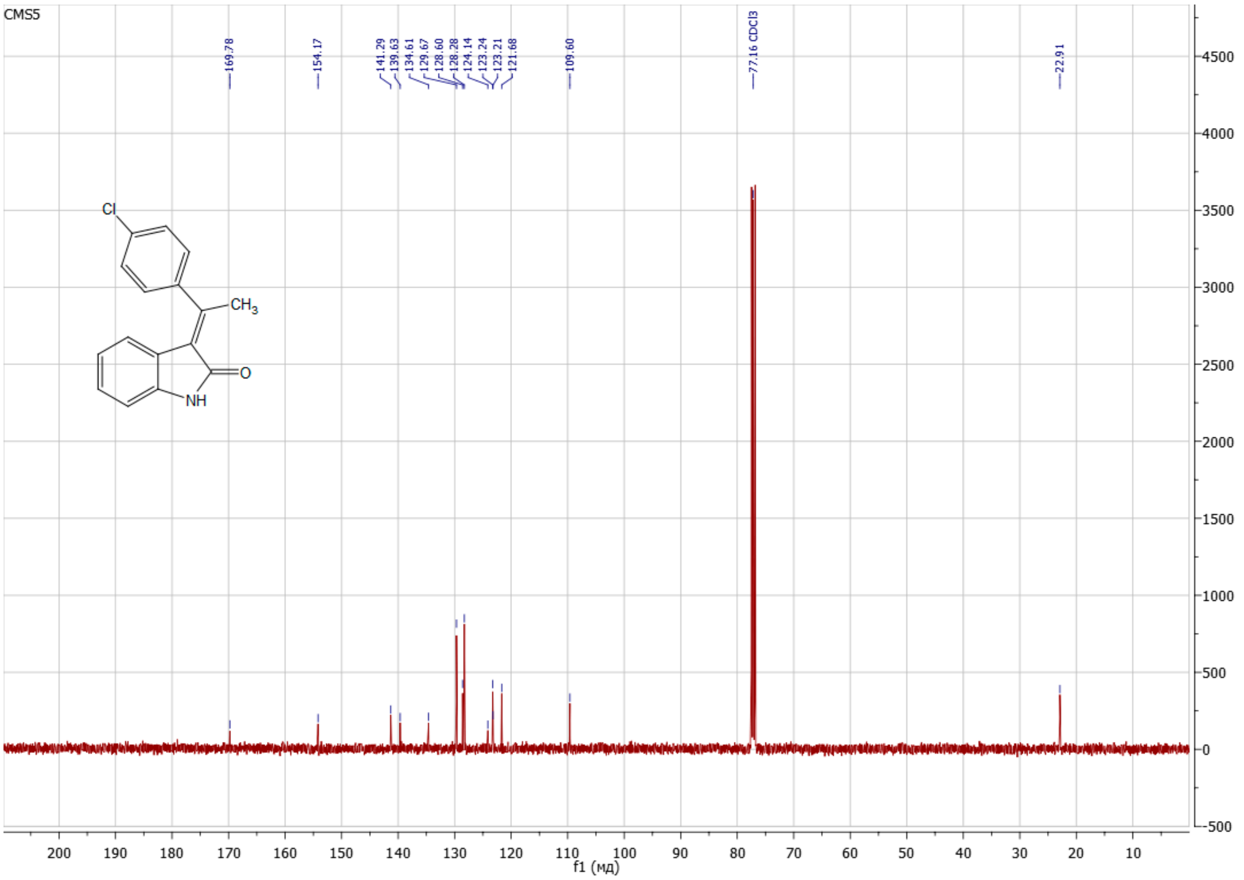

Compound *Z*-**2e**

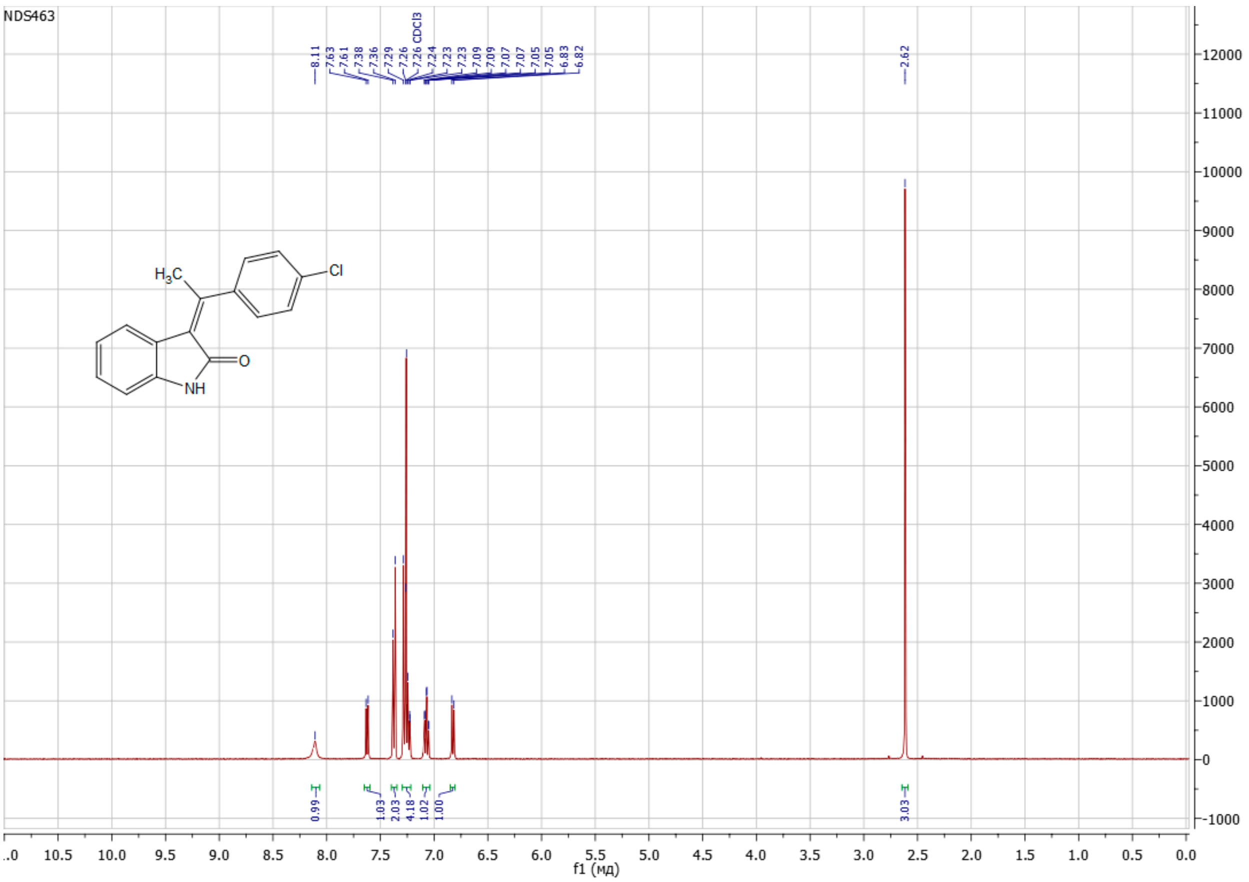

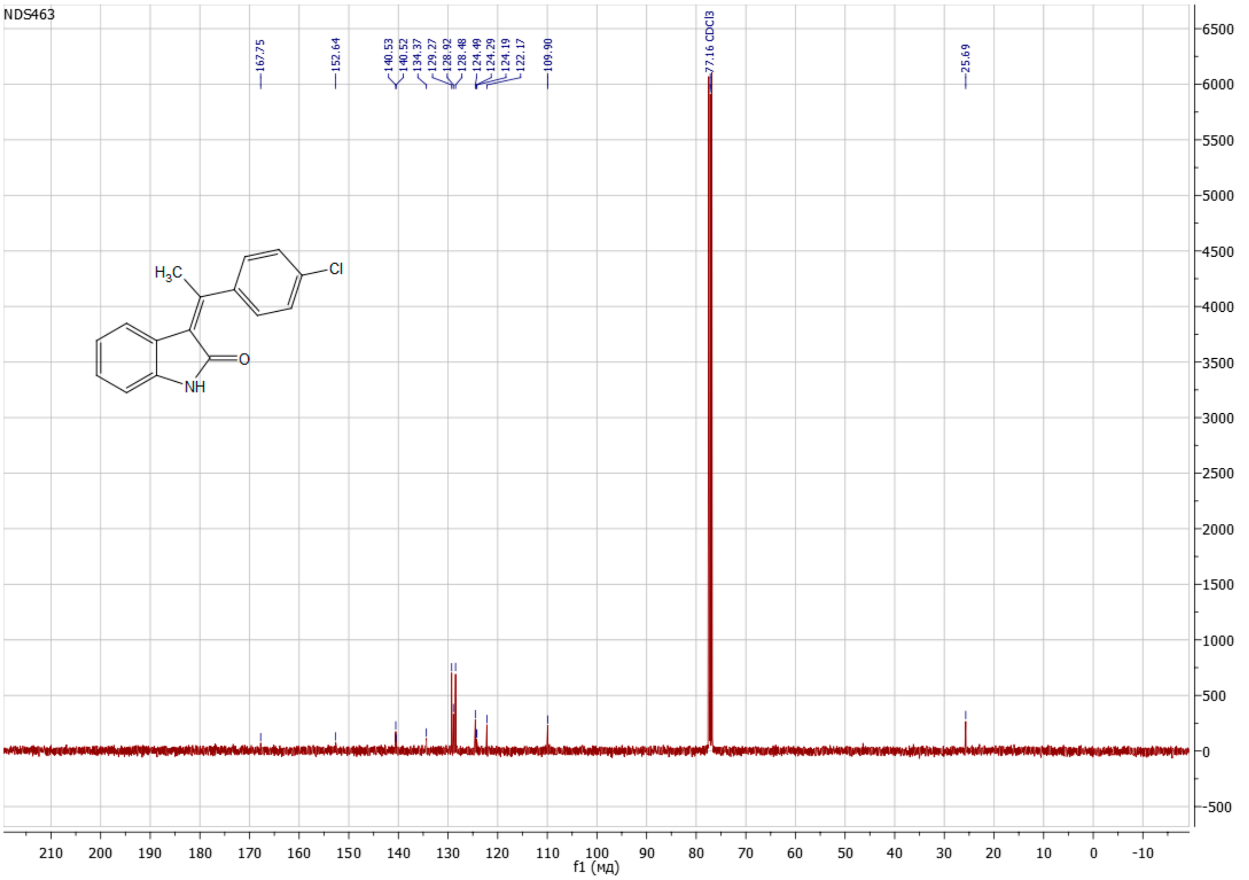

Compound *E*-**2f**

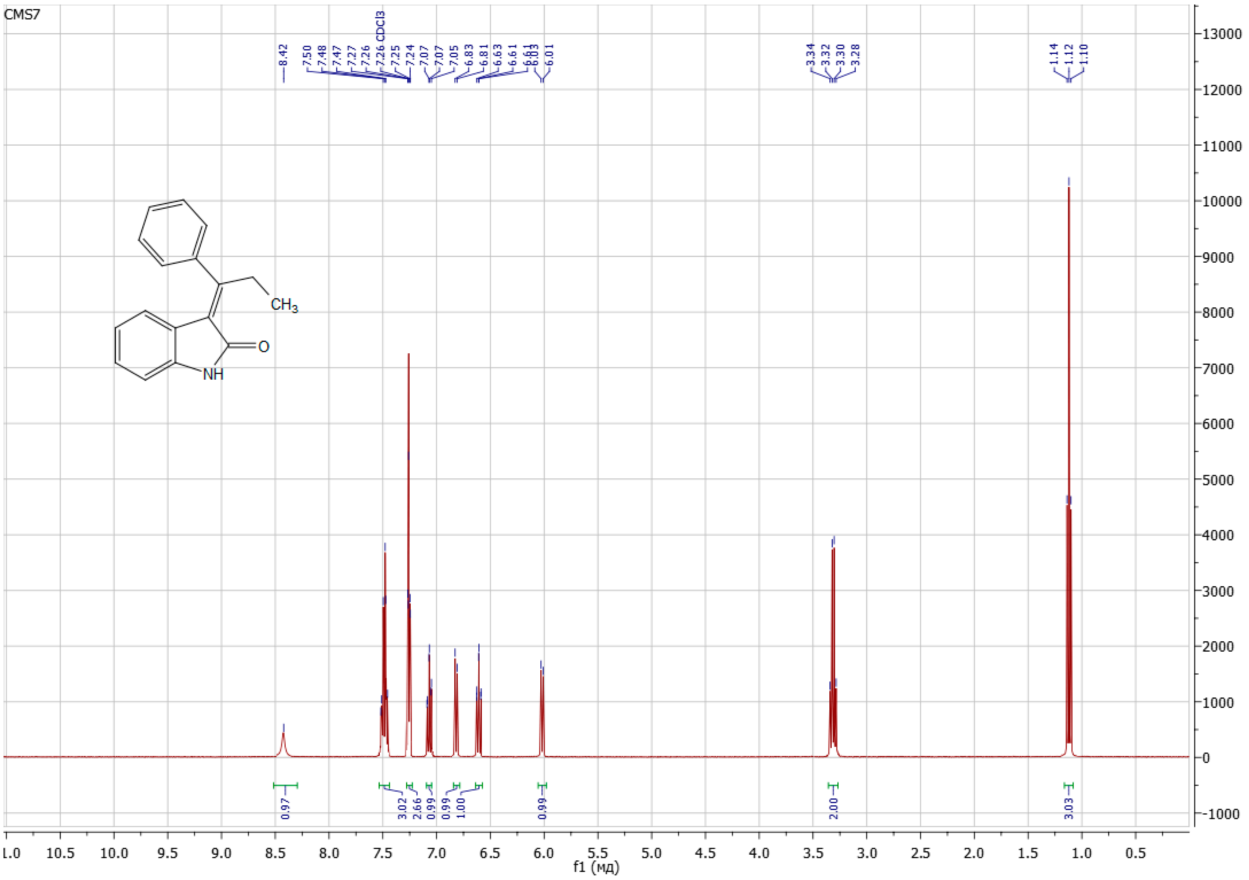

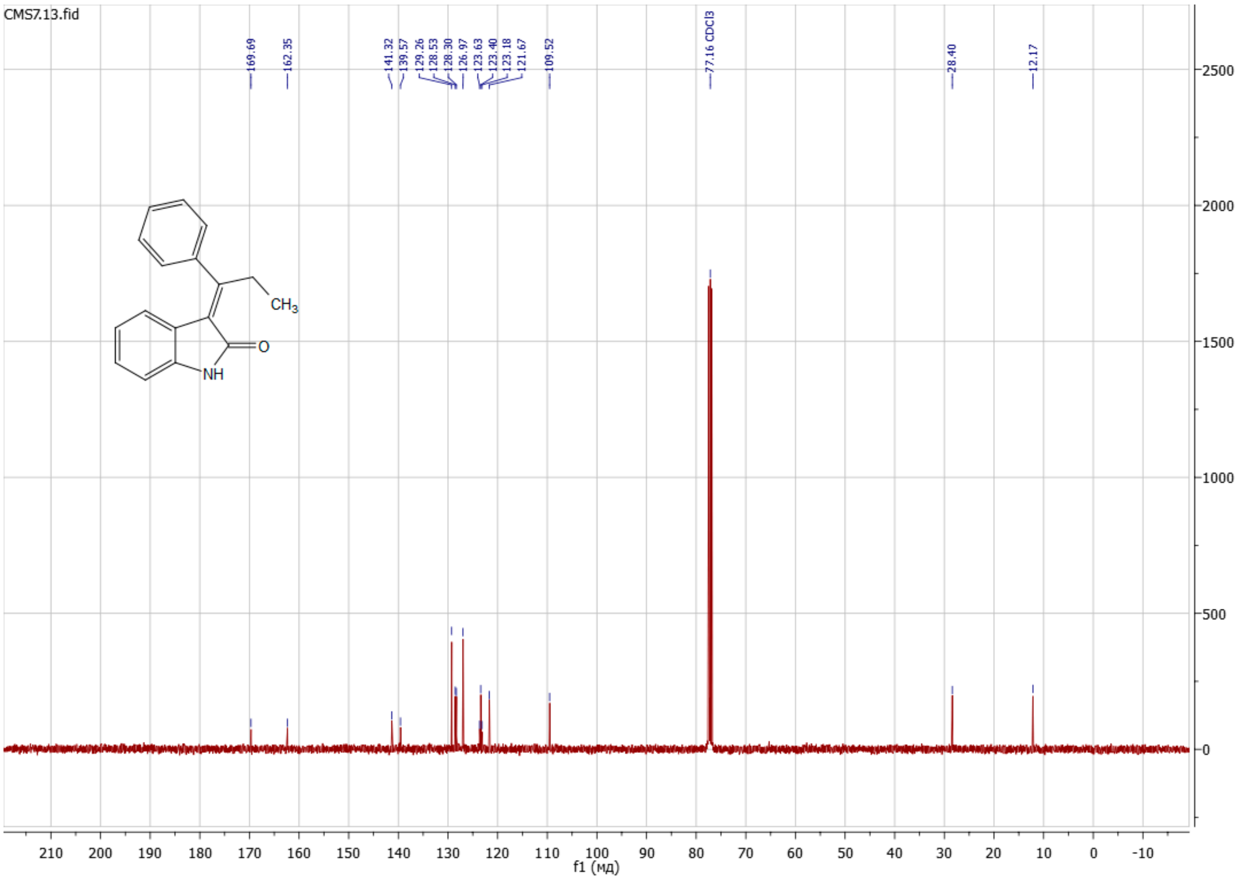

Compound *Z*-**2f**

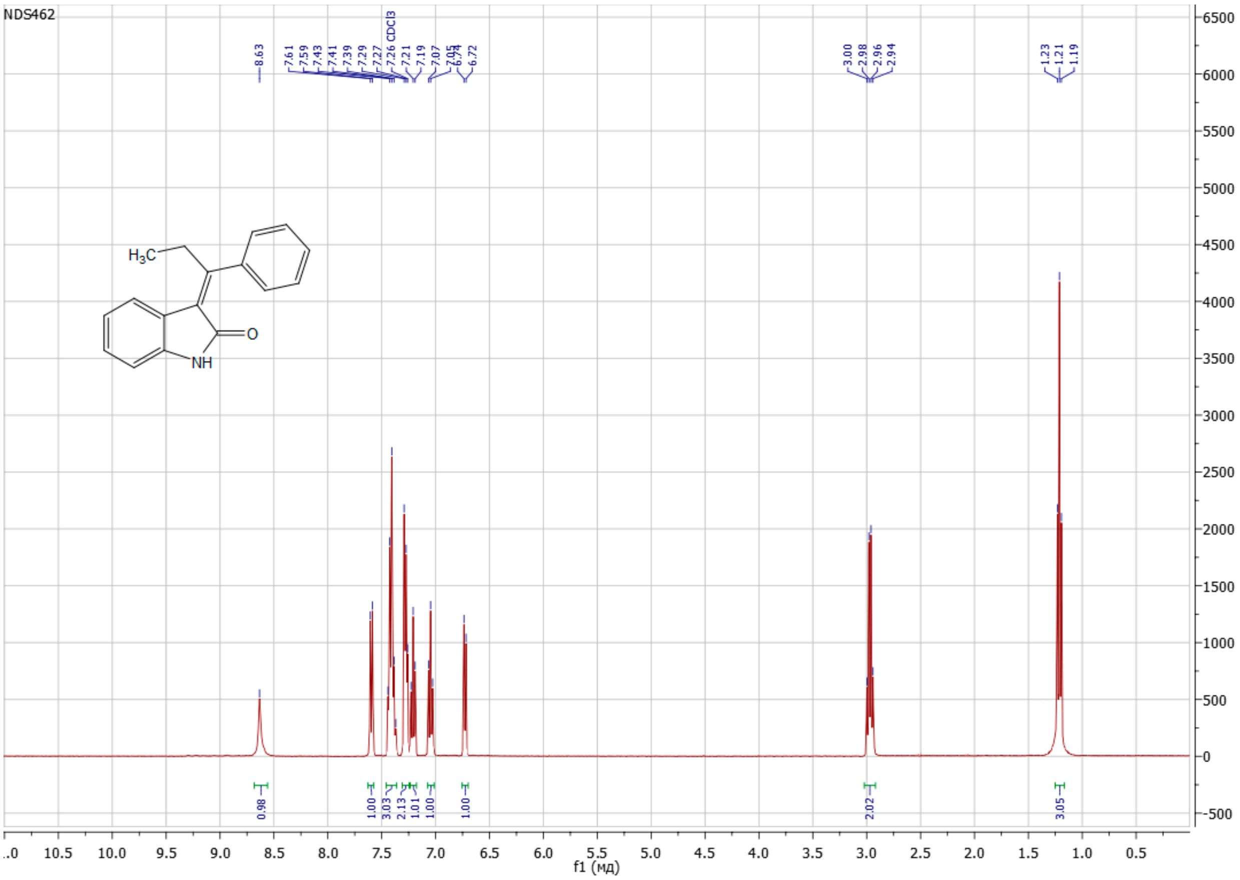

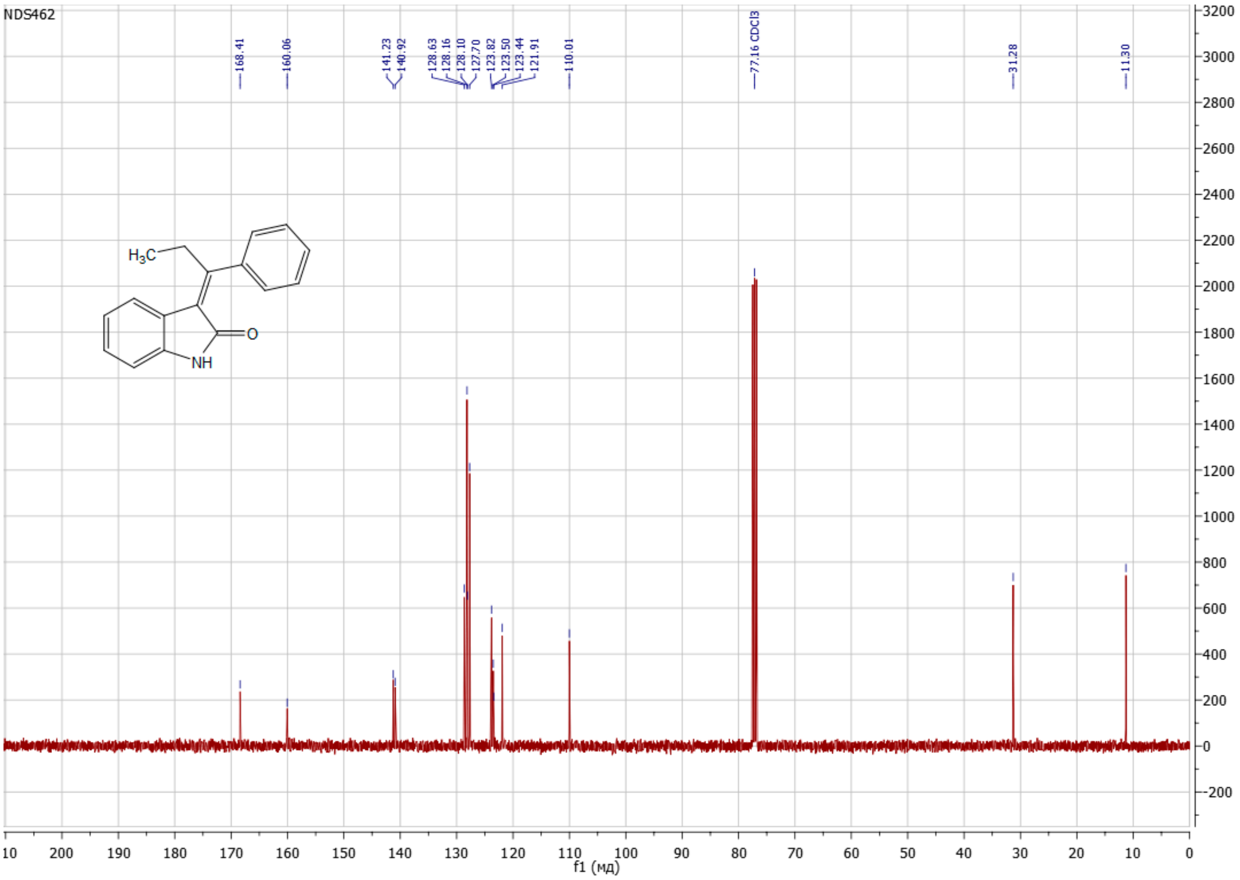

Compound *E*-**3c**

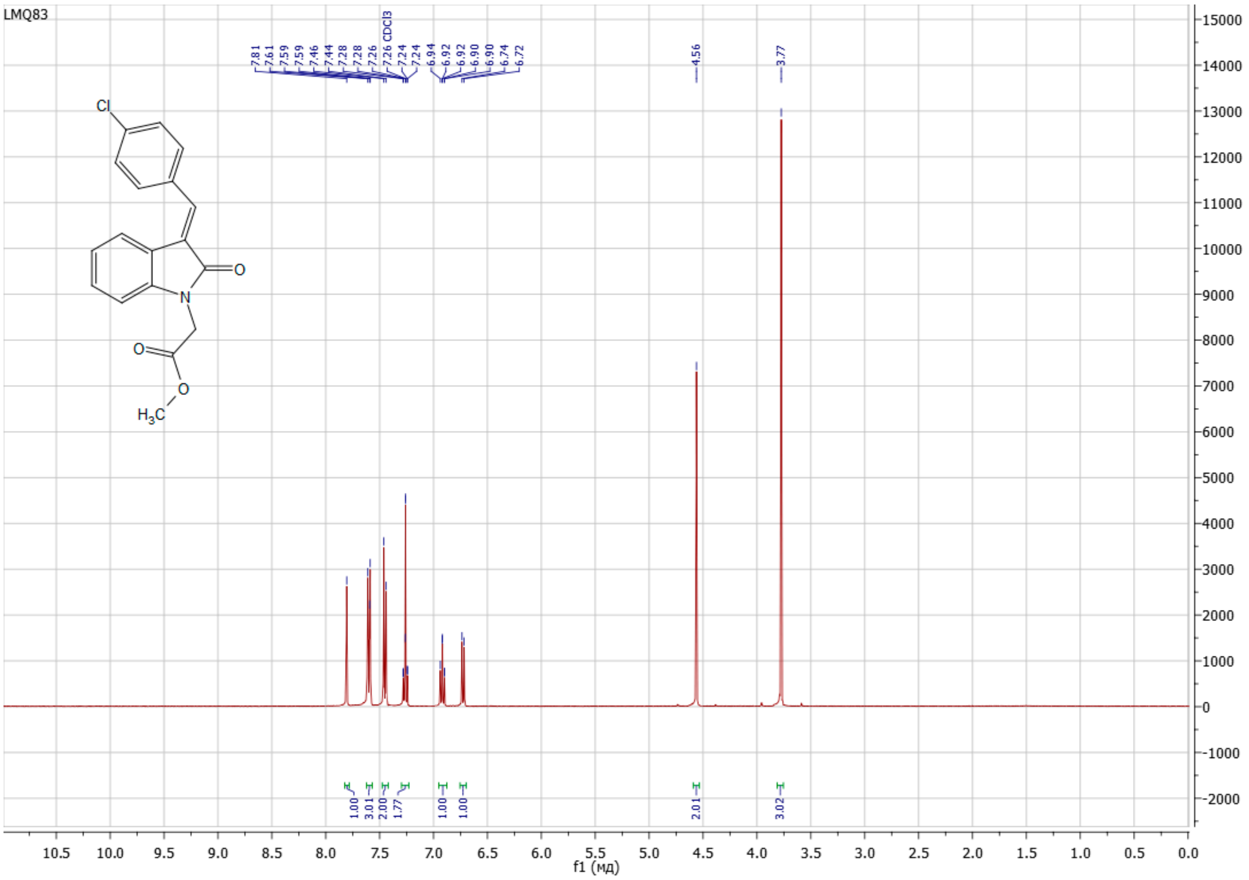

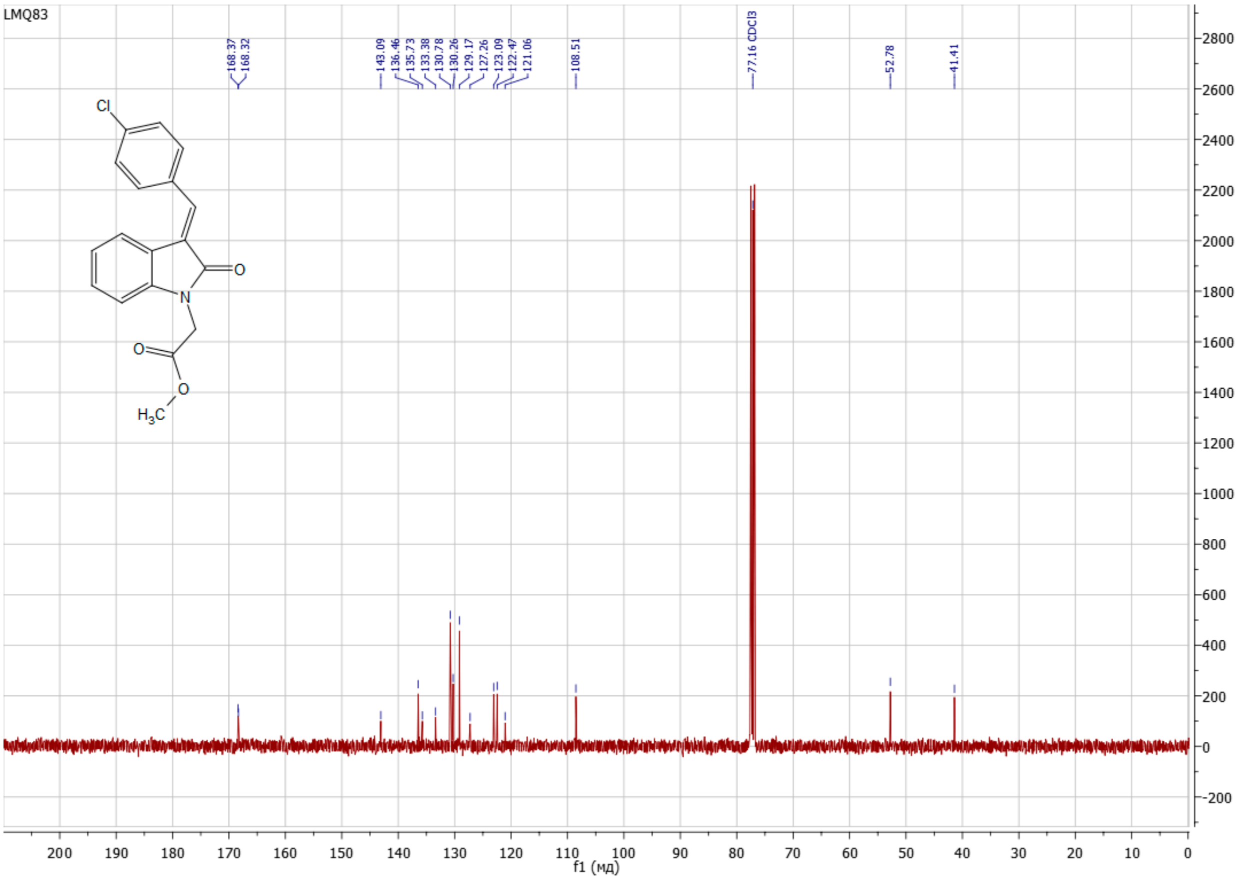

Compound *Z*-**3c**

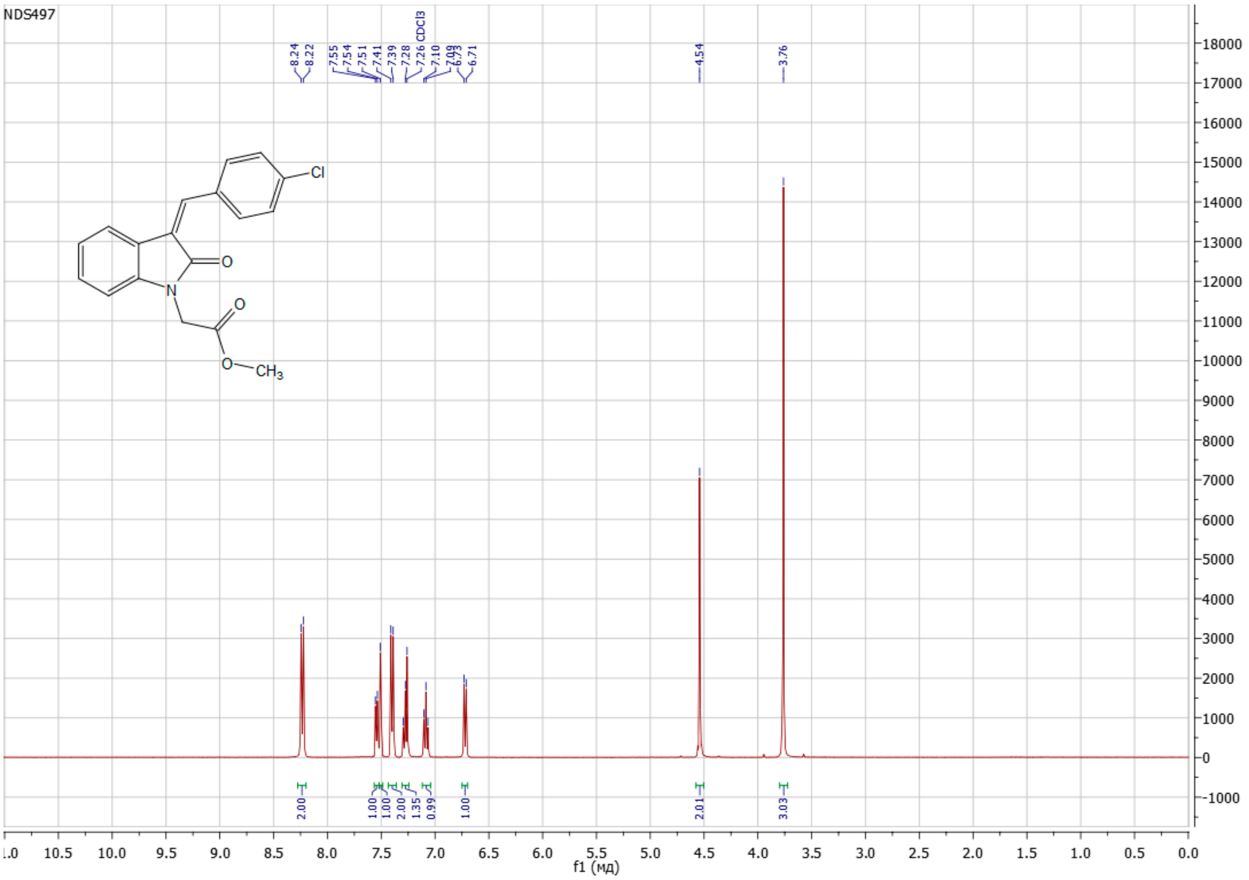

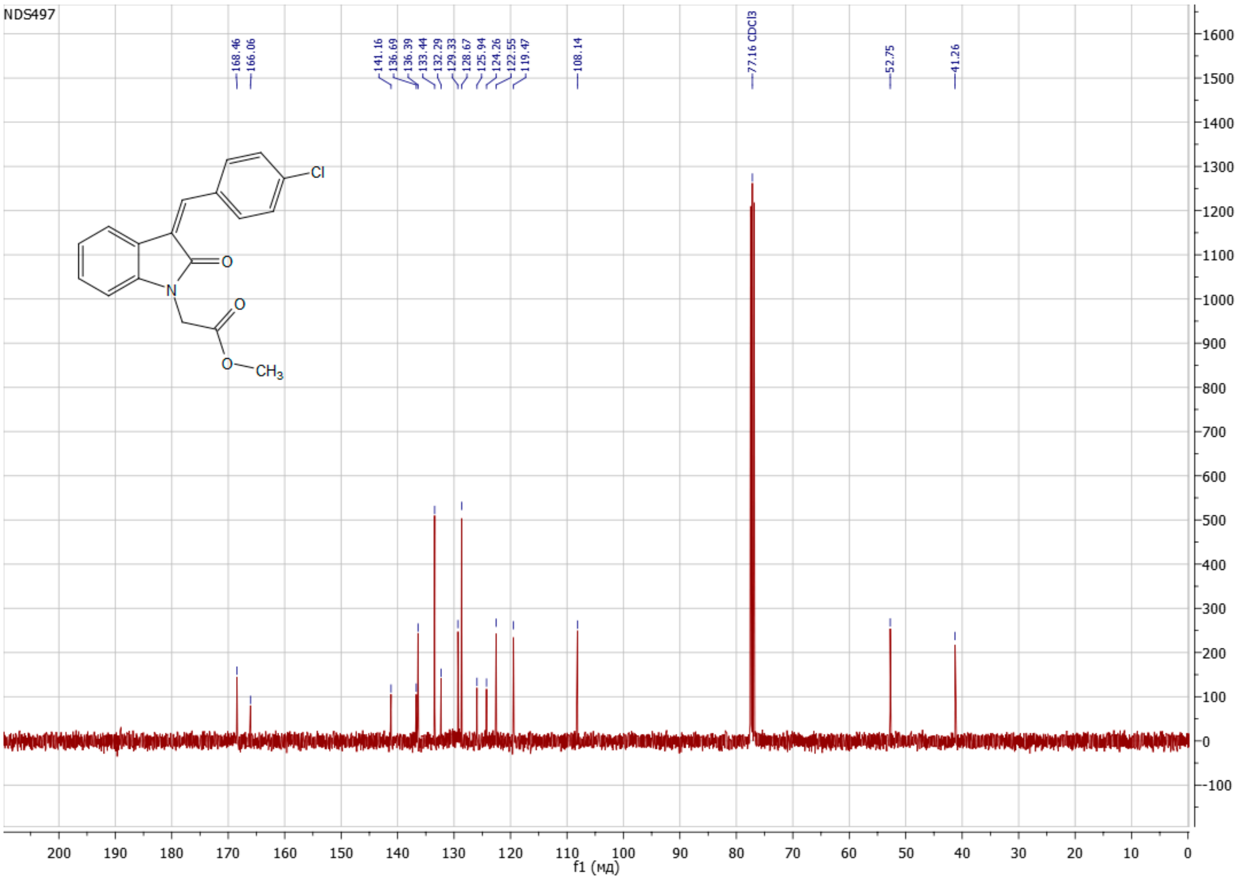

Compound *E*-**3d**

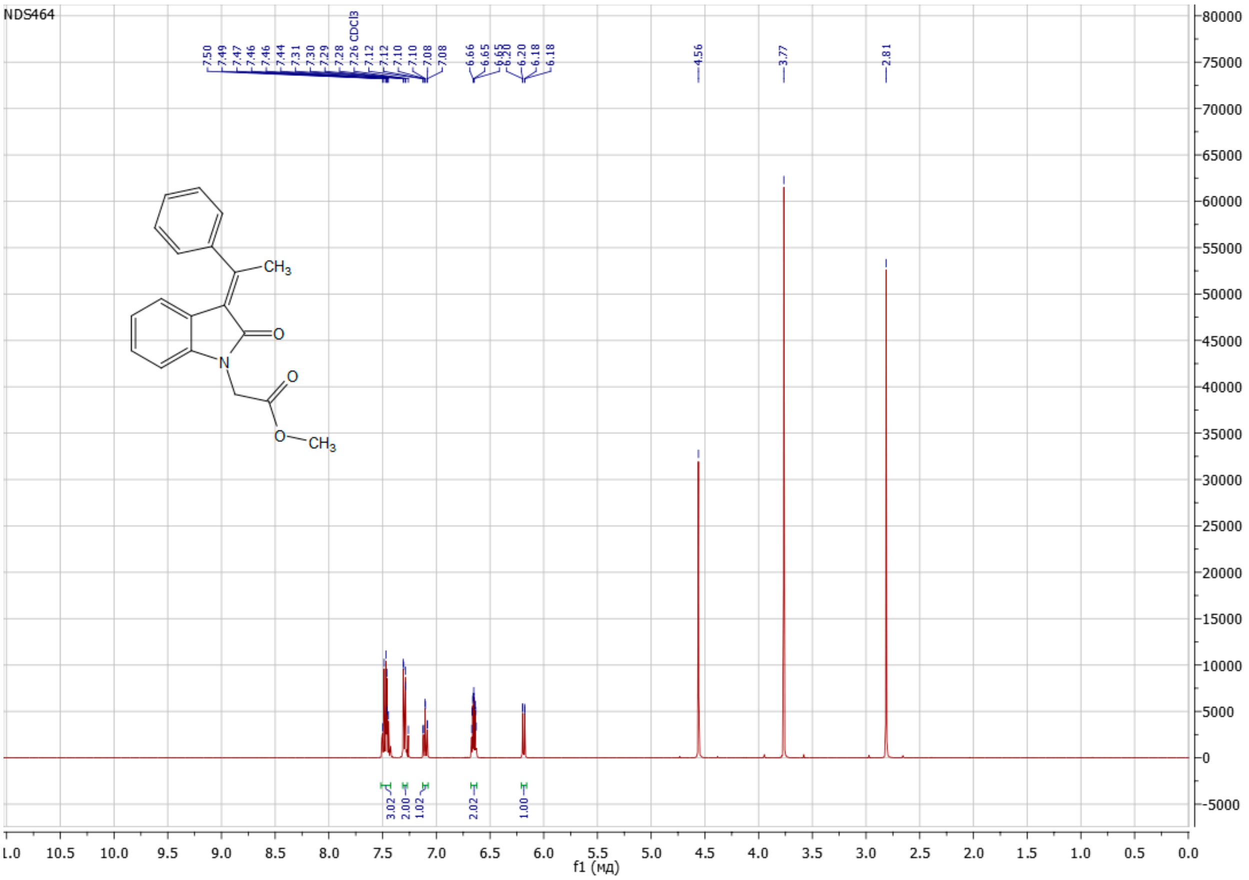

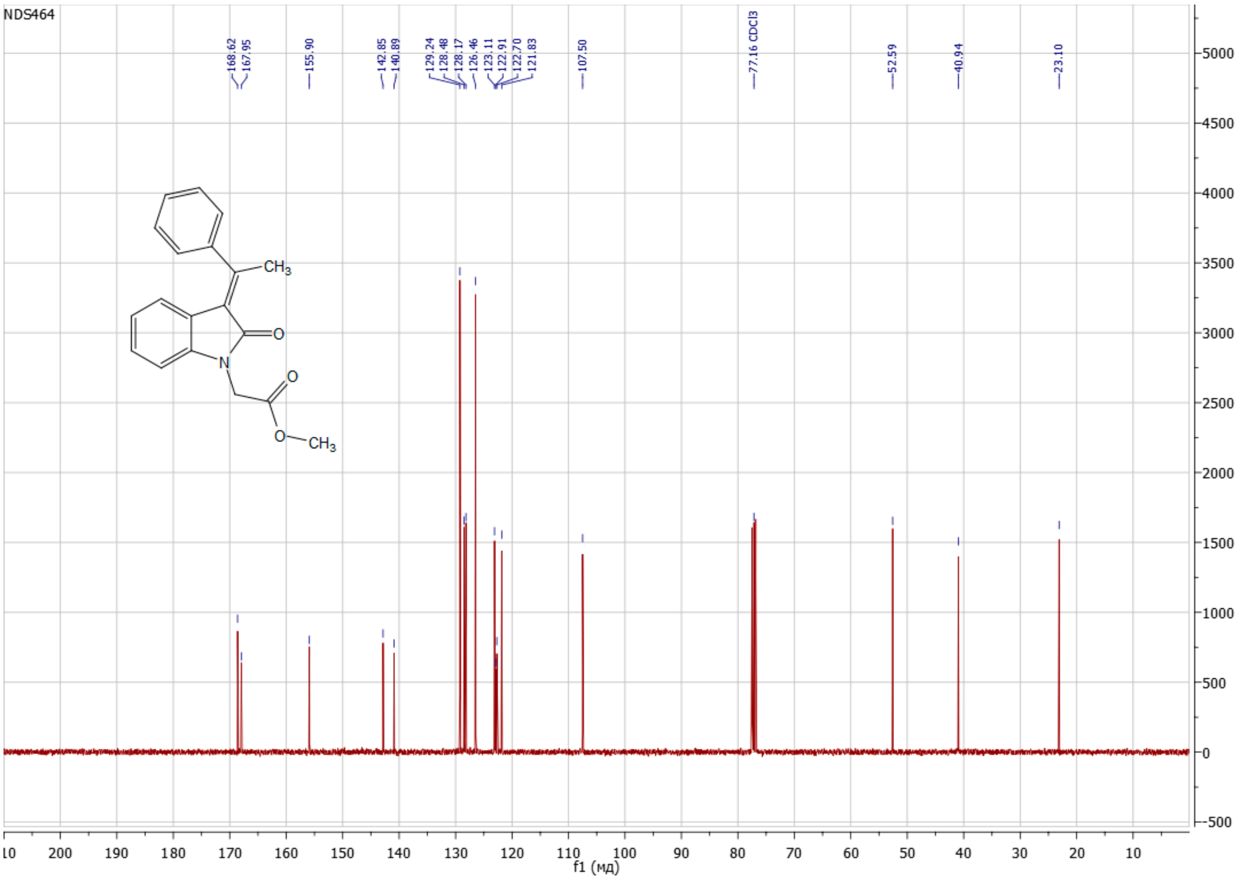

Compound *Z*-**3d**

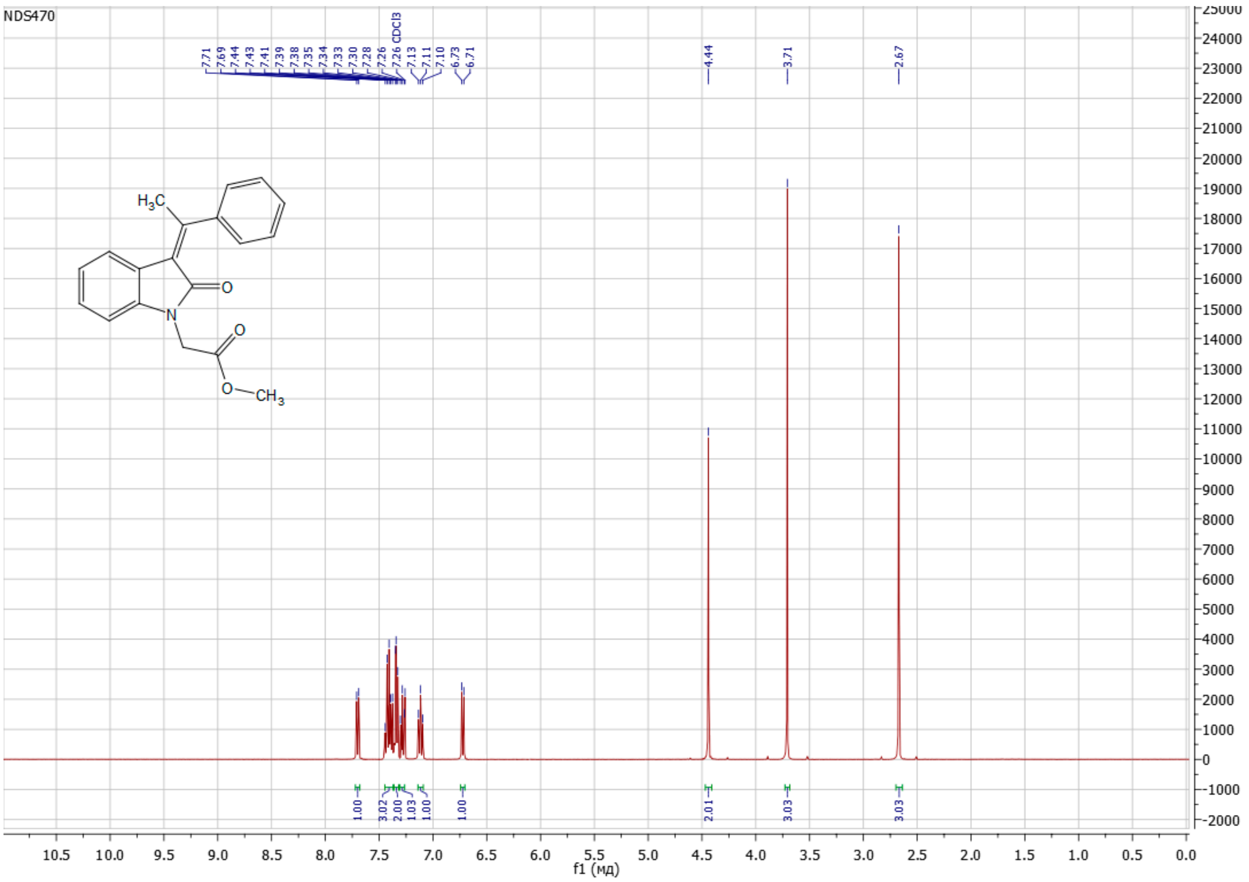

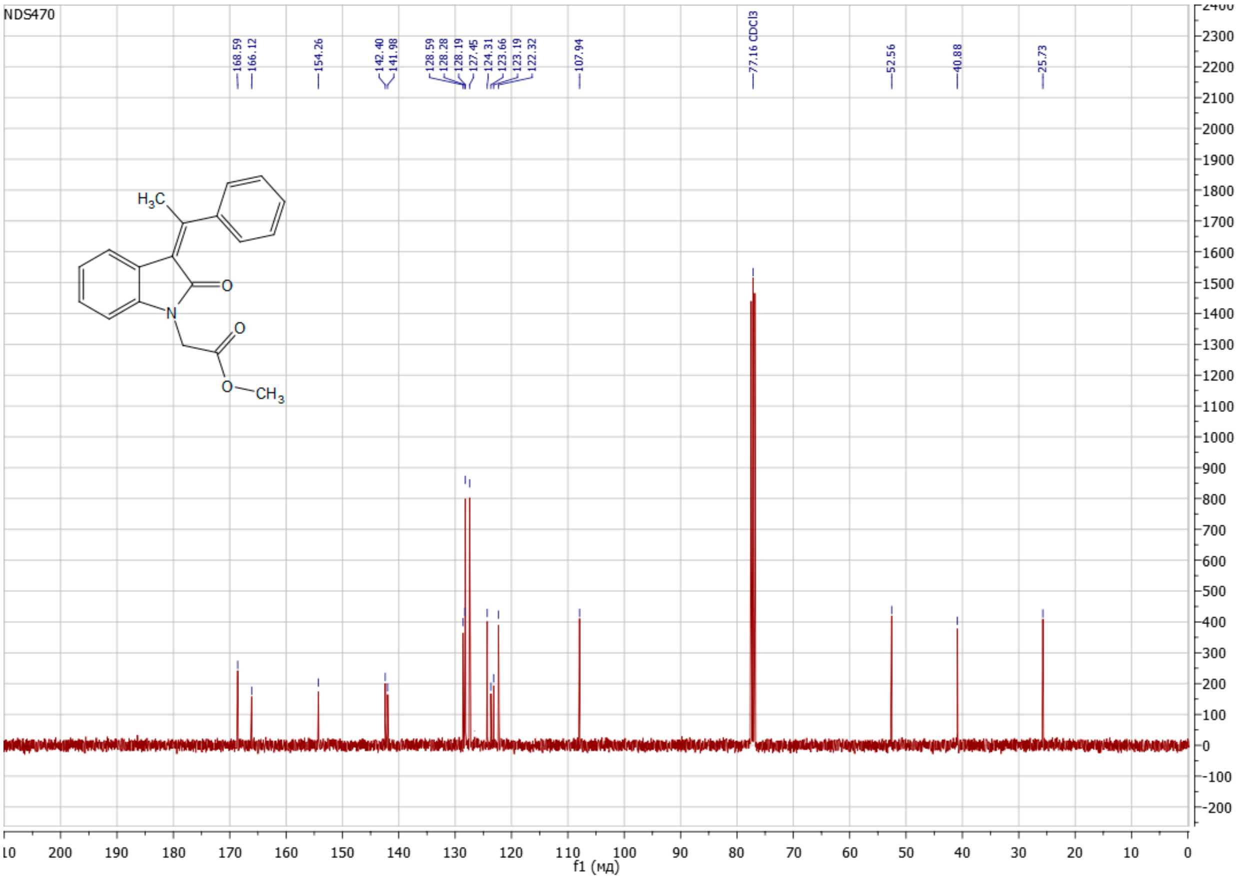

Compound *E*-**3e**

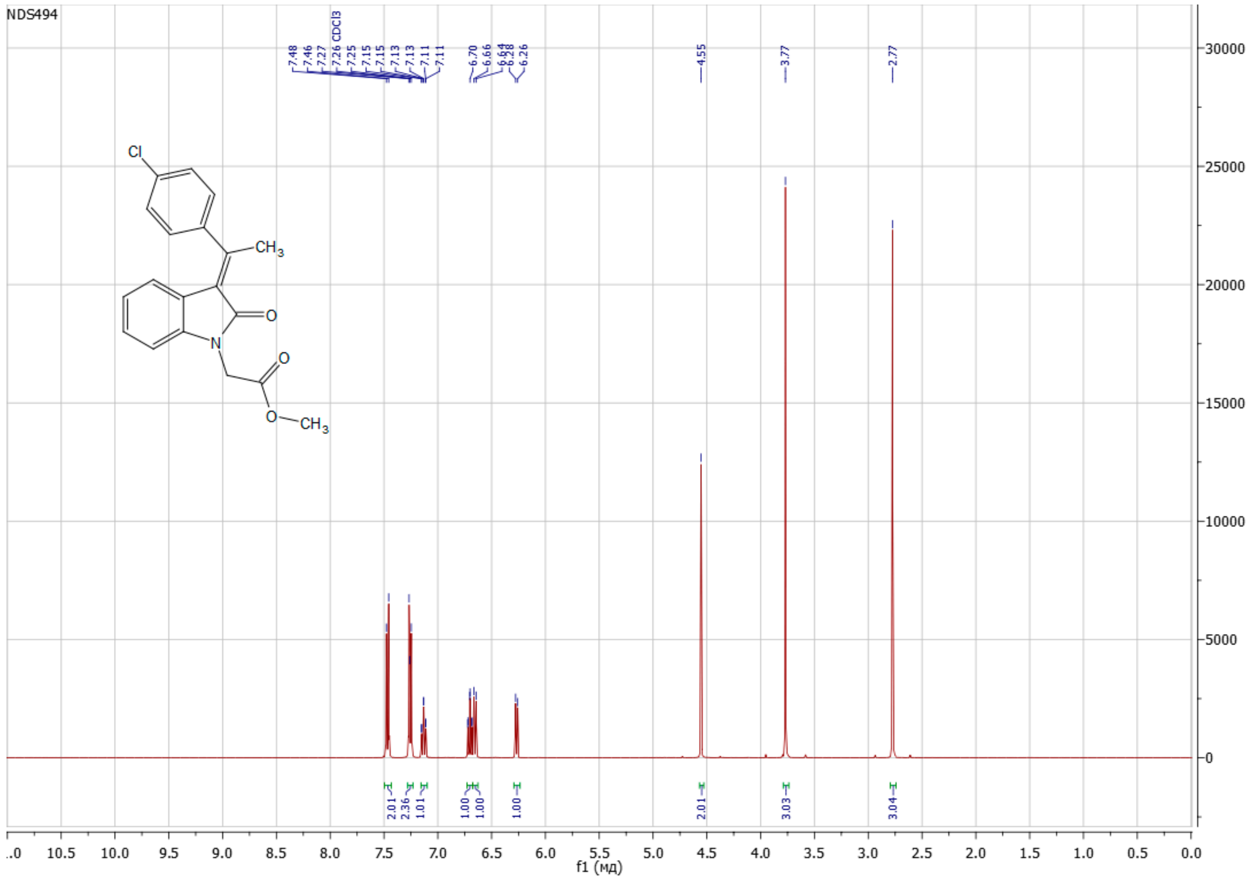

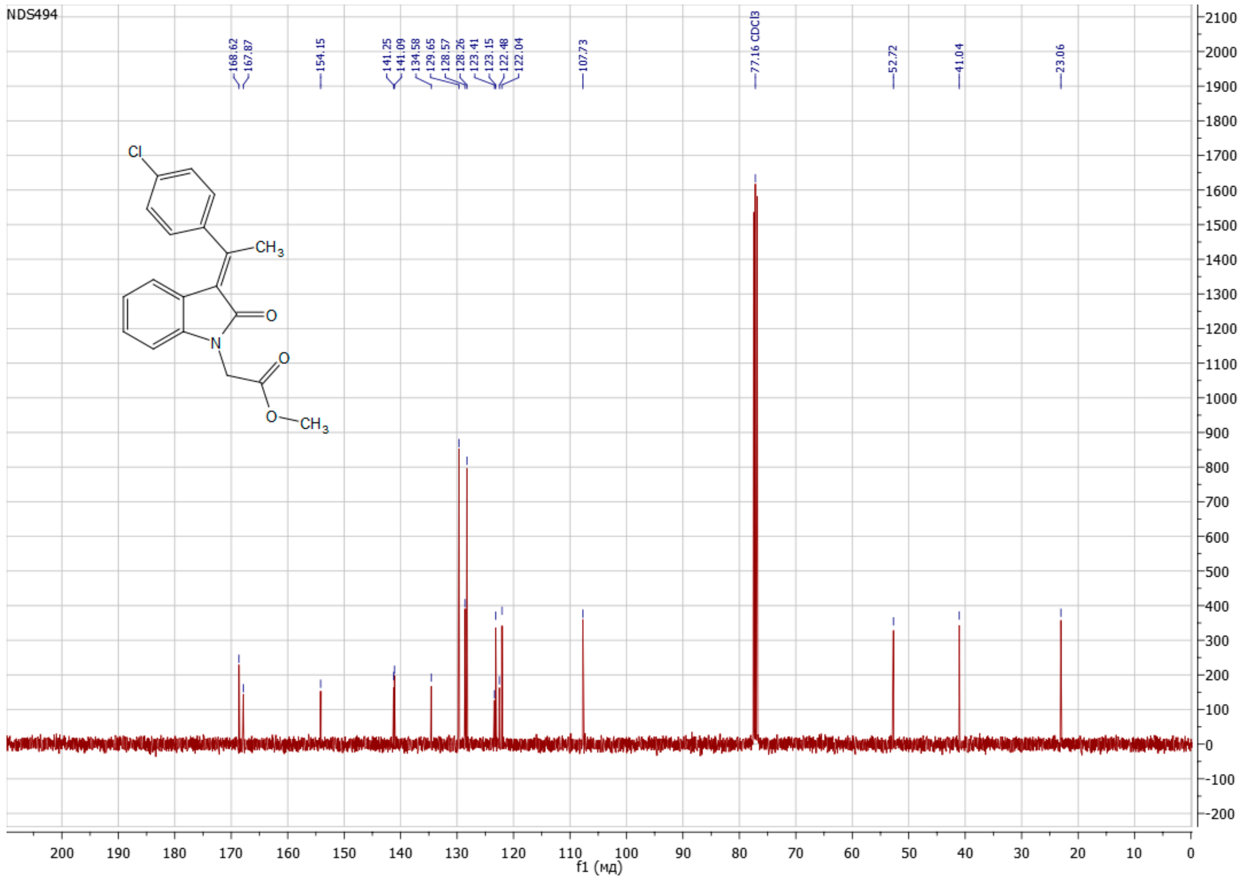

Compound *Z*-**3e**

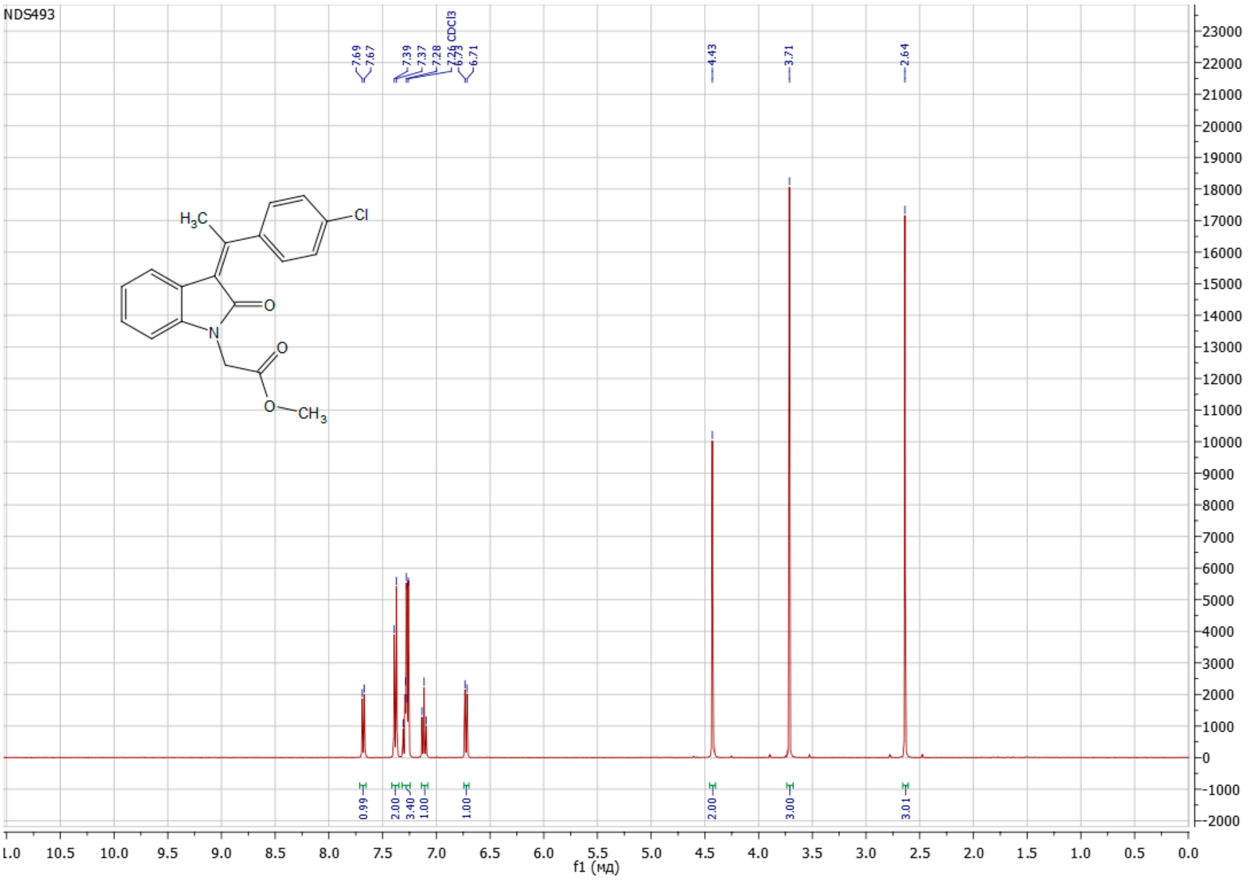

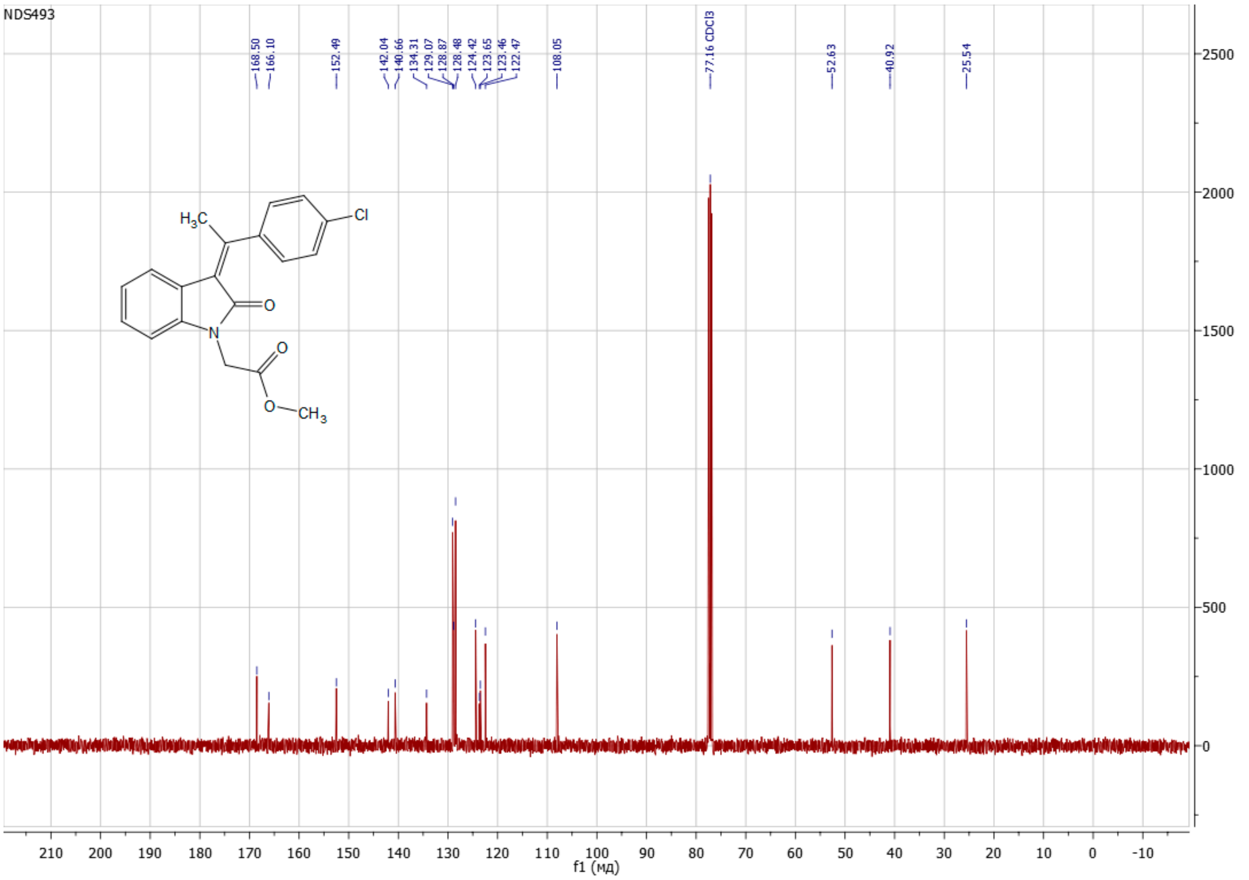

UV-Vis analysis

The spectral characteristics of isolated *E*- and *Z*-isomers differ significantly. For all the studied compounds, two absorption regions are typical. When comparing the spectra of the compounds with the spectrum of original oxindole, for which only one absorption region and, therefore, one maximum is observed, the appearance of an additional absorption region in the long-wavelength part of the spectrum indicates the appearance of a more extended conjugated system with an aromatic fragment (Figure S3).

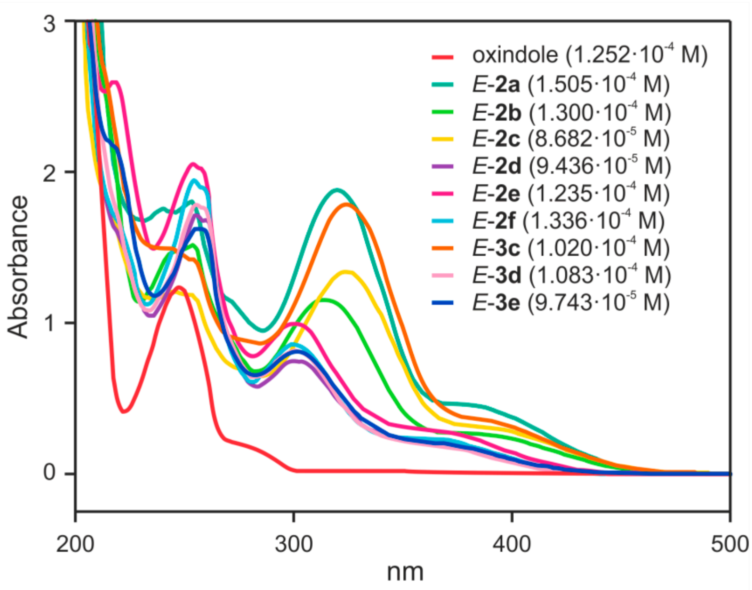

Figure S3. Absorption spectra of *E*-isomers compared with the absorption spectrum of oxindole in methanol.

In the course of the study, the influence of substituents on the spectral characteristics of the studied compounds was also evaluated. When passing from compound **2a** to **2c** and from 2d to **2e**, as well as when passing from unsubstituted derivatives to compounds with substitution at the nitrogen atom, no significant differences were observed in the absorption spectra of the isomers within the corresponding isomers. Thus, the substitution of halogen atom at the considered positions of the phenyl fragment does not have a significant effect on the conjugated system of the studied molecules, as well as substituents at position 1, since they are isolated from the conjugated system. A different picture is observed when the substituent is directly bound to the conjugated system. For example, when passing from **2a** to **2d**, a significant change in the spectral pattern is observed: the absorption intensity in the short-wavelength region increases significantly, while in the long-wavelength region, on the contrary, it decreases. In this case, the absorption maximum in the short-wavelength region undergoes a bathochromic shift, while a hypsochromic shift is observed for the absorption maximum in the long-wavelength region.

A detailed analysis of the obtained spectra revealed a number of characteristic differences within the isomeric pairs (see Figure 4 of the main text):

1) *Z*-isomer spectrum is shifted to the long-wavelength region (bathochromic shift);

2) absorption minimum of *Z*-isomers in the short-wavelength region is lower than that of *E*-isomers;

3) in most cases, the hyperchromic effect (more intense absorption at the same concentration) is observed for *Z*-isomers.

Such characteristic differences are probably associated with the configuration of the studied compounds in a solution. It can be assumed that the shift in the spectrum of *Z*-isomers to the red region and the increase in the intensity of the absorption peak in the short-wavelength region are associated with an increase in the conjugation effect in the system. Thus, it is known that *E*-isomers of 3-benzylidene oxindole derivatives can be stabilized due to T-shaped π-stacking that occurs between two aromatic systems. In this case, the mutual arrangement of phenyl rings tends to be perpendicular. In turn, *Z*-isomers can take up a more planar configuration due to free rotation of the phenyl fragment, which is also observed in crystals of aldehyde derivatives [2]. Although similar configuration was not revealed in the obtained structures of *Z*-**2d** and *Z*-**2e**, C(11)–C(16) cycles are disordered over two crystallographically unique positions (0.5/0.5 in *Z*-**2d** and 0.95/0.05 in *Z*-**2e**) realized by the rotation around C(10)–C(11) bond (see structures in the X-ray data section). The possibility of such disorder not observed for *E*-**2d** and *E*-**2e** indicates less strict orientation of the phenyl fragment, which is in agreement with the calculated density reduction. Thus, the configuration of the molecule has a significant effect on the conjugation effect.

Spectrophotometric characteristics

Compound	Concentration, mM	Isomer	λ_{max}, nm	Absorption, units	ε, L/mol·cm	λ_{min}, nm	Absorption, units	ε, L/mol·cm
**2a**	0.151	*E*	240	1.753	11565	232	1.676	11032
			253	1.802	11909	246	1.731	11430
			321	1.883	12470	286	0.950	6260
		Z	265	1.727	11476	245	1.347	8938
			336	1.725	11471	295	0.850	5644
**2b**	0.130	*E*	254	1.519	11959	229	1.138	8672
			314	1.171	9231	284	0.678	5193
		Z	259	1.878	14263	232	0.740	5554
			329	1.385	10536	291	0.733	5583
**2c**	0.087	*E*	245	1.202	13549	236	1.156	12913
			324	1.340	15239	285	0.646	7261
		Z	268	1.302	15063	238	0.684	7812
			341	1.797	21875	297	0.708	8180
**2d**	0.094	*E*	256	1.713	17664	235	1.045	10812
			301	0.749	7812	283	0.581	5994
		Z	259	2.531	26967	232	0.755	7504
			307	0.819	8429	288	0.659	6703
**2e**	0,123	*E*	218	2.596	21042	214	2.529	20507
			254	2.052	16661	236	1.492	12100
			300	0.994	8078	281	0.778	6320
		Z	259	3.484	28154	231	1.090	8771
			313	1.194	9646	288	0.928	7487
**2f**	0.134	*E*	255	1.954	15006	230	1.154	8426
			300	0.864	6642	267	0.585	4203
		Z	260	2.413	17859	230	0.717	5565
			303	0.749	5537	288	0.651	4730
**3c**	0.102	*E*	242	1.493	14684	238	1.490	14655
			325	1.786	17526	285	0.866	8456
		Z	269	1.529	15143	239	0.873	8686
			341	2.385	23642	297	0.895	8867
**3d**	0.108	*E*	255	1.784	16326	233	1.082	9884
			301	0.814	7459	283	0.640	5864

		Z	260	2.544	23554	231	0.758	6972
			313	0.897	8301	288	0.678	6270
**3e**	0.097	*E*	256	1.469	15596	237	1.057	11221
			302	0.736	7817	283	0.589	6236
		Z	260	1.966	21306	232	0.627	6728
			317	0.741	8063	289	0.541	5526

UV-Vis spectra

Compound *E*-**2a** ($C = 1.505\times10^{-4}$ M)

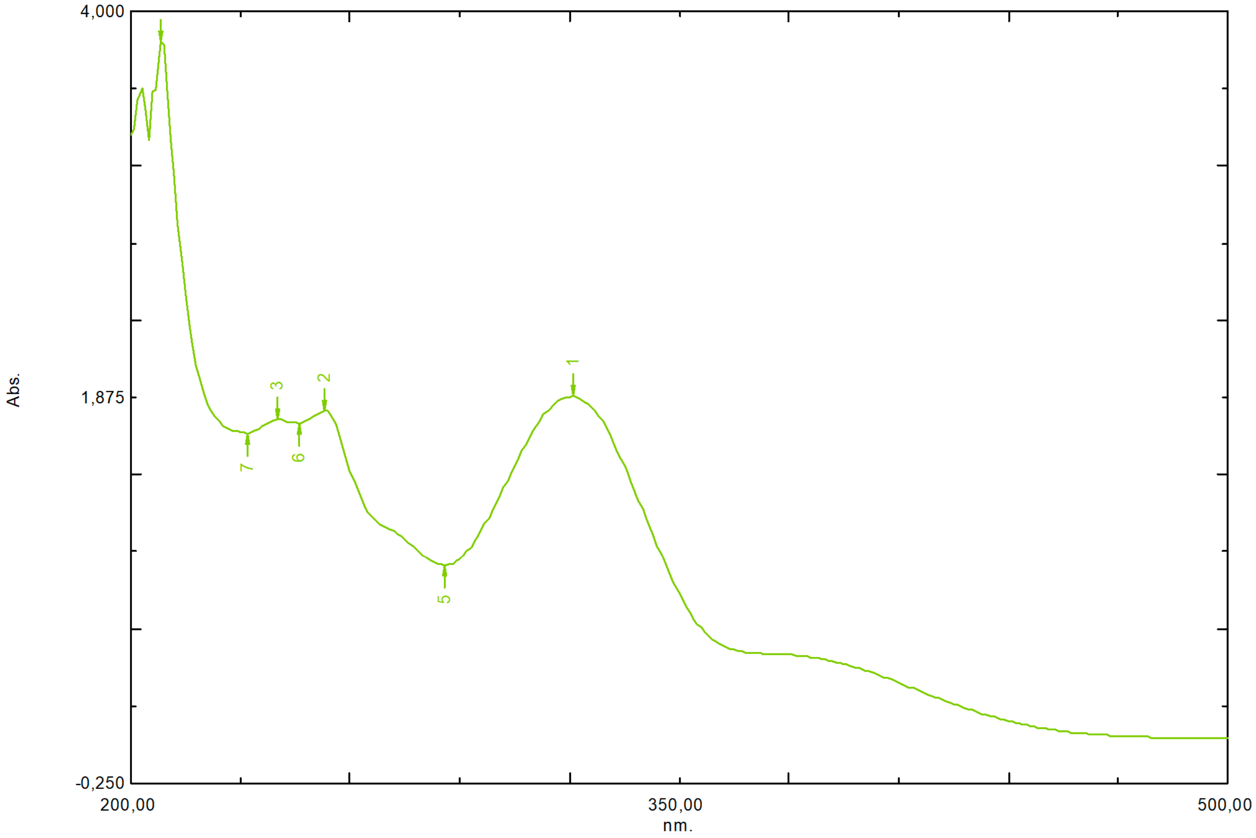

Compound *Z*-**2a** ($C = 1.505\times10^{-4}$ M)

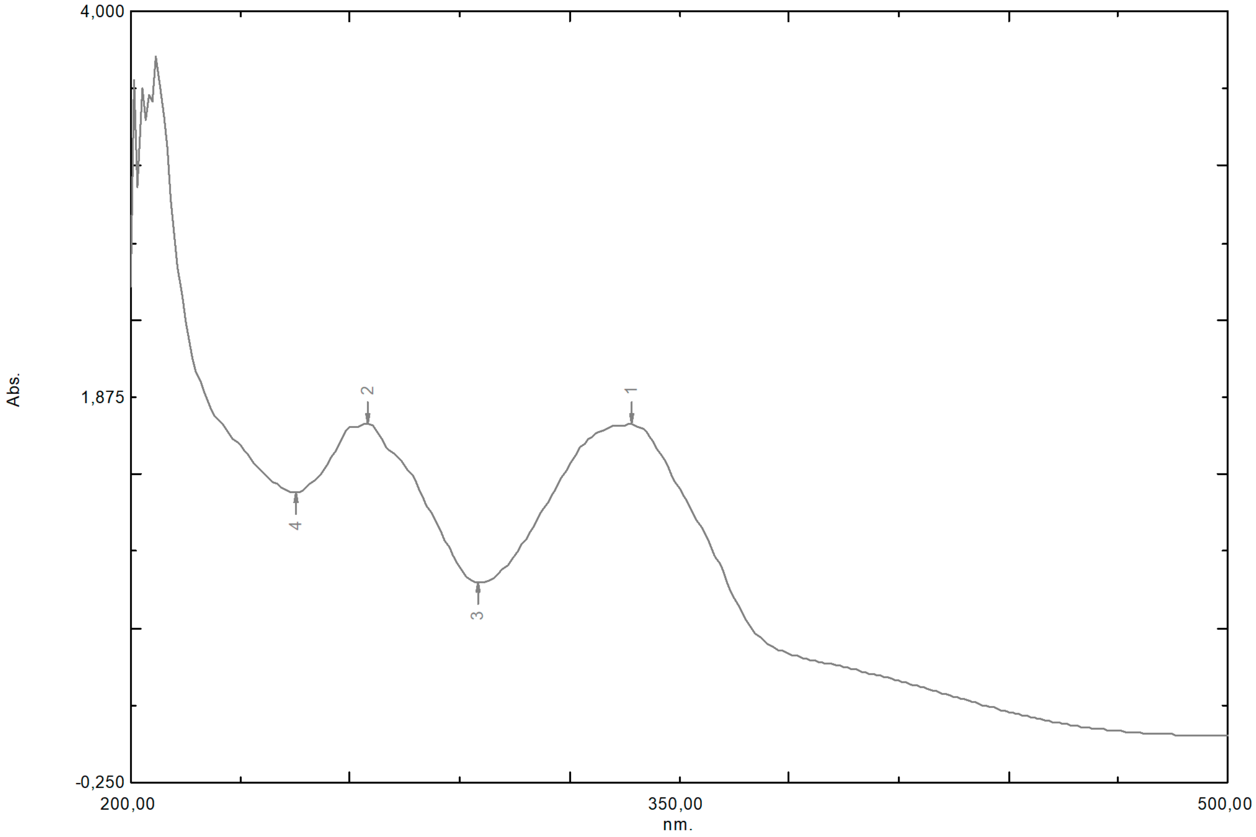

Compound *E*-**2b** ($C = 1.3 \times 10^{-4}$ M)

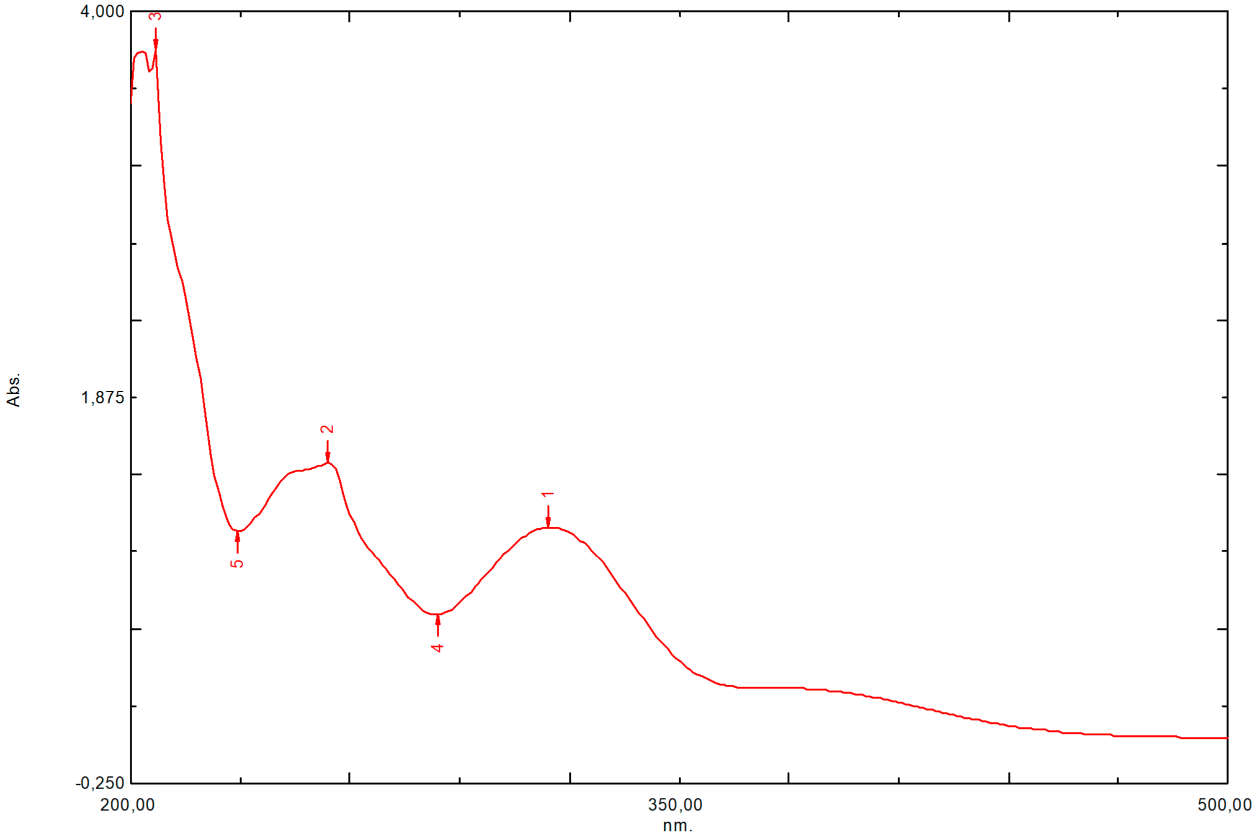

Compound *Z*-**2b** ($C = 1.3 \times 10^{-4}$ M)

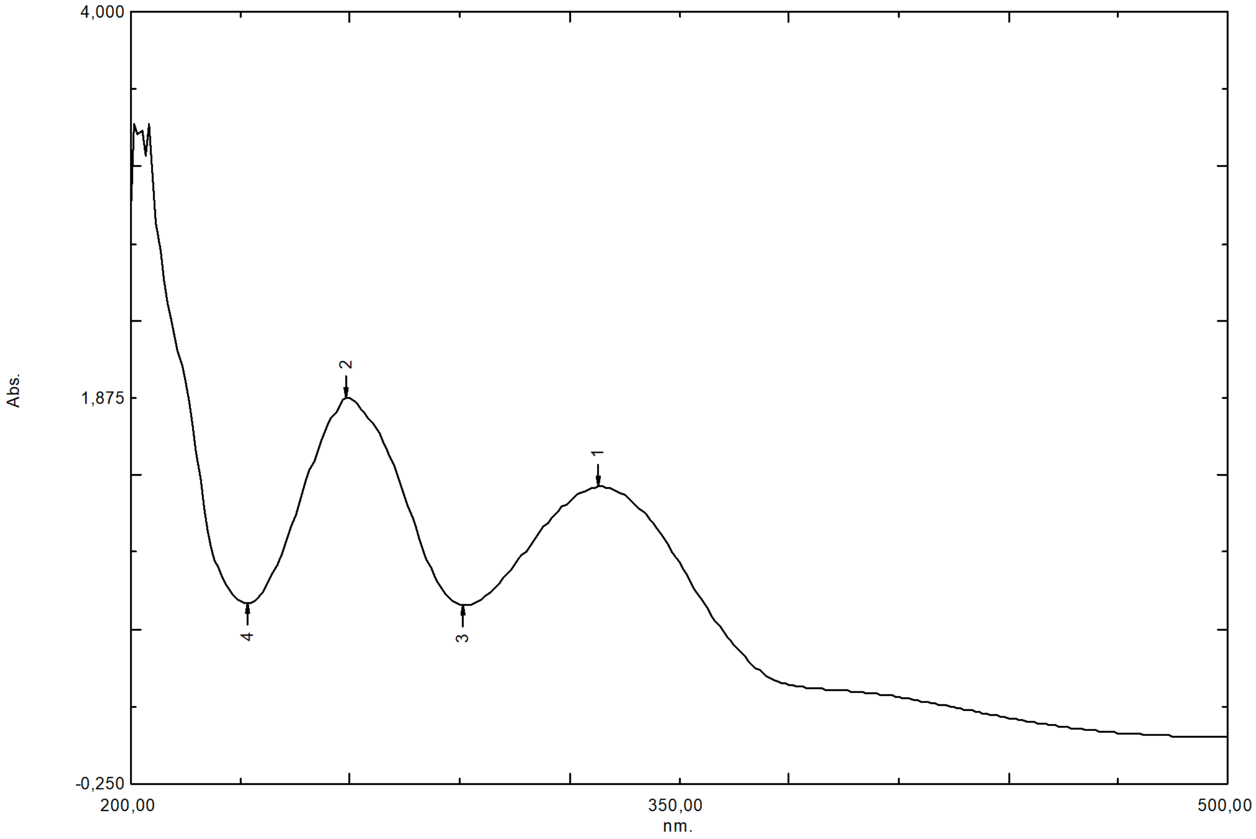

Compound *E*-**2c** ($C = 8.682 \times 10^{-5}$ M)

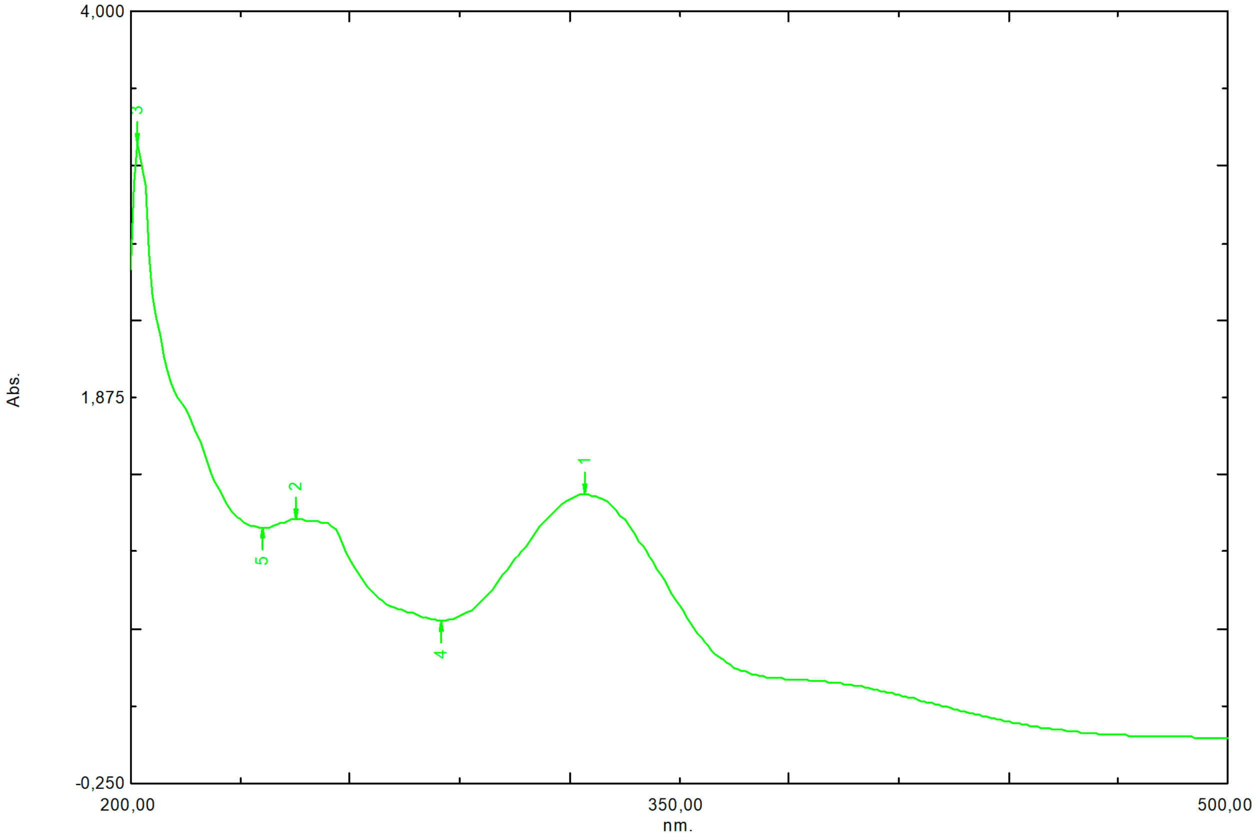

Compound *Z*-**2c** ($C = 8.682 \times 10^{-5}$ M)

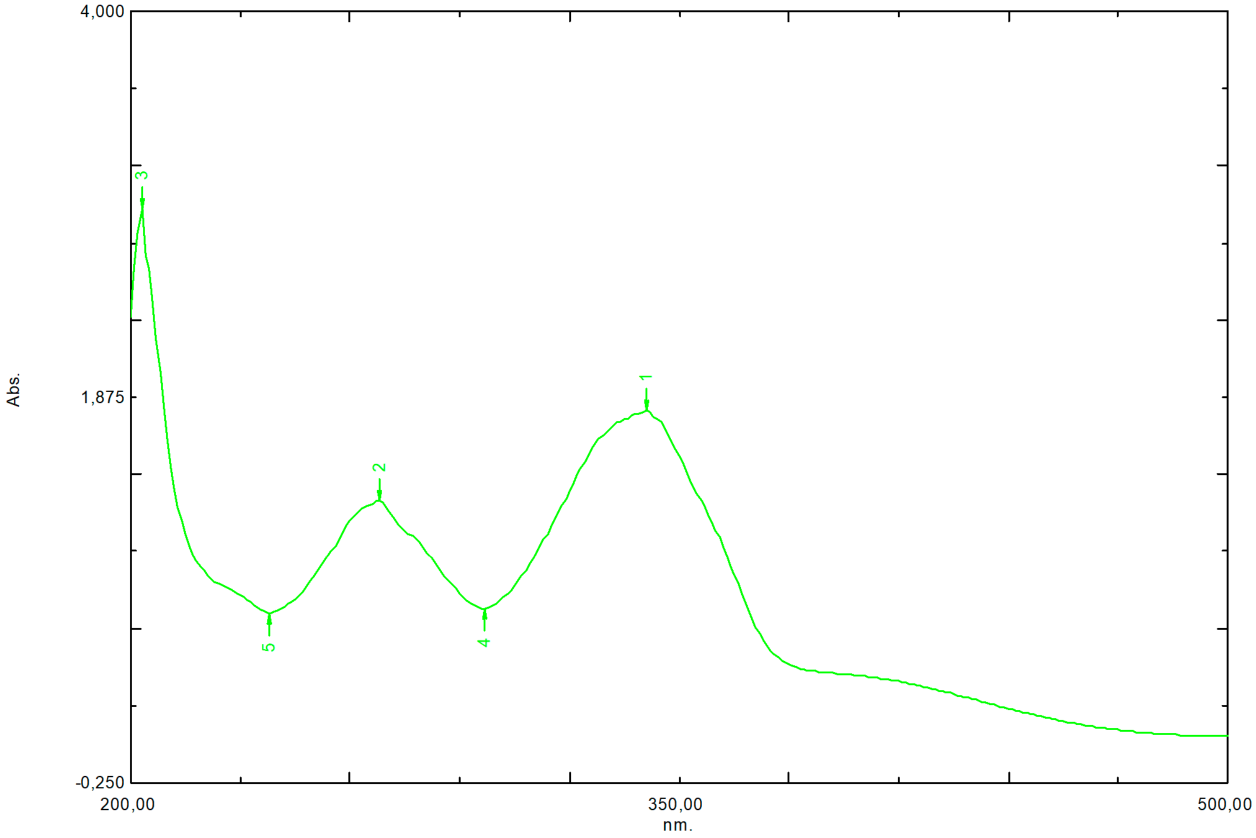

Compound *E*-**2d** ($C = 9.436 \times 10^{-5}$ M)

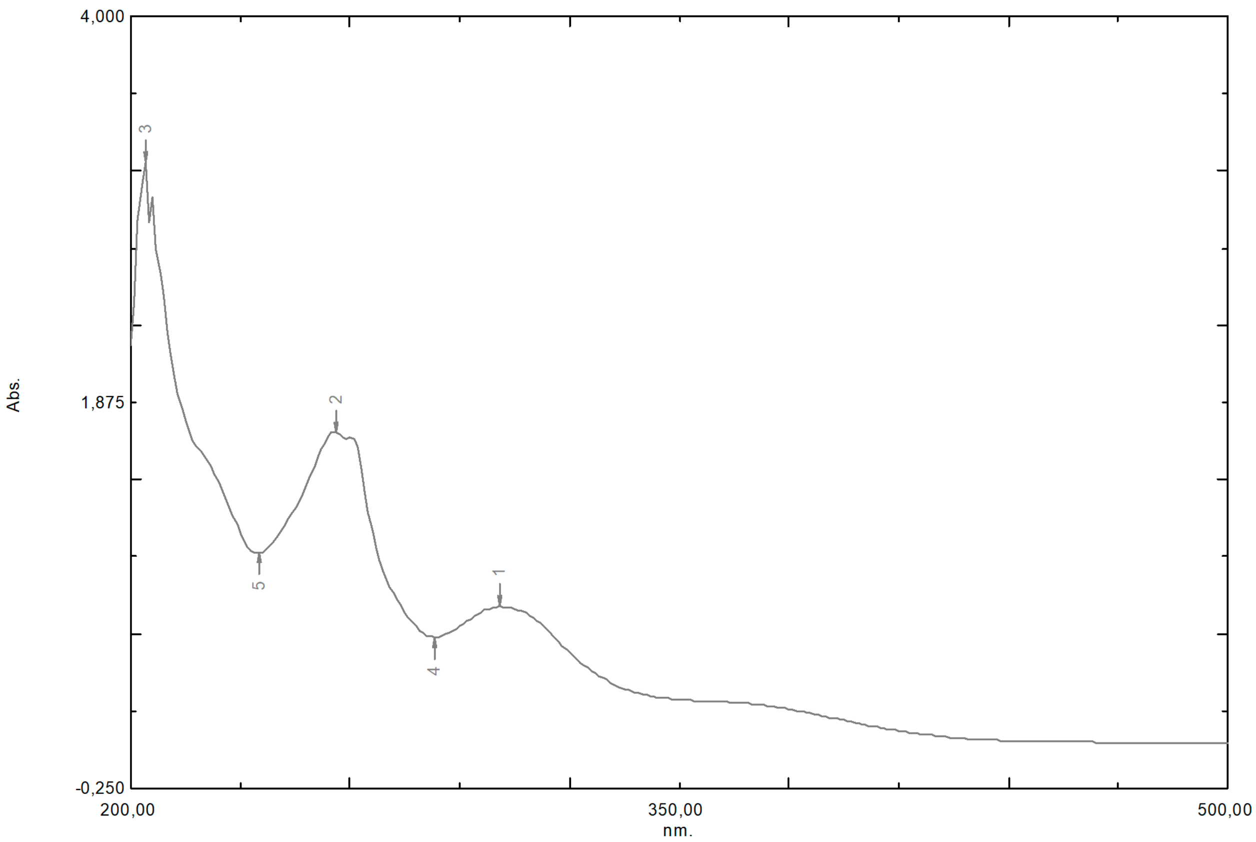

Compound *Z*-**2d** ($C = 9.436 \times 10^{-5}$ M)

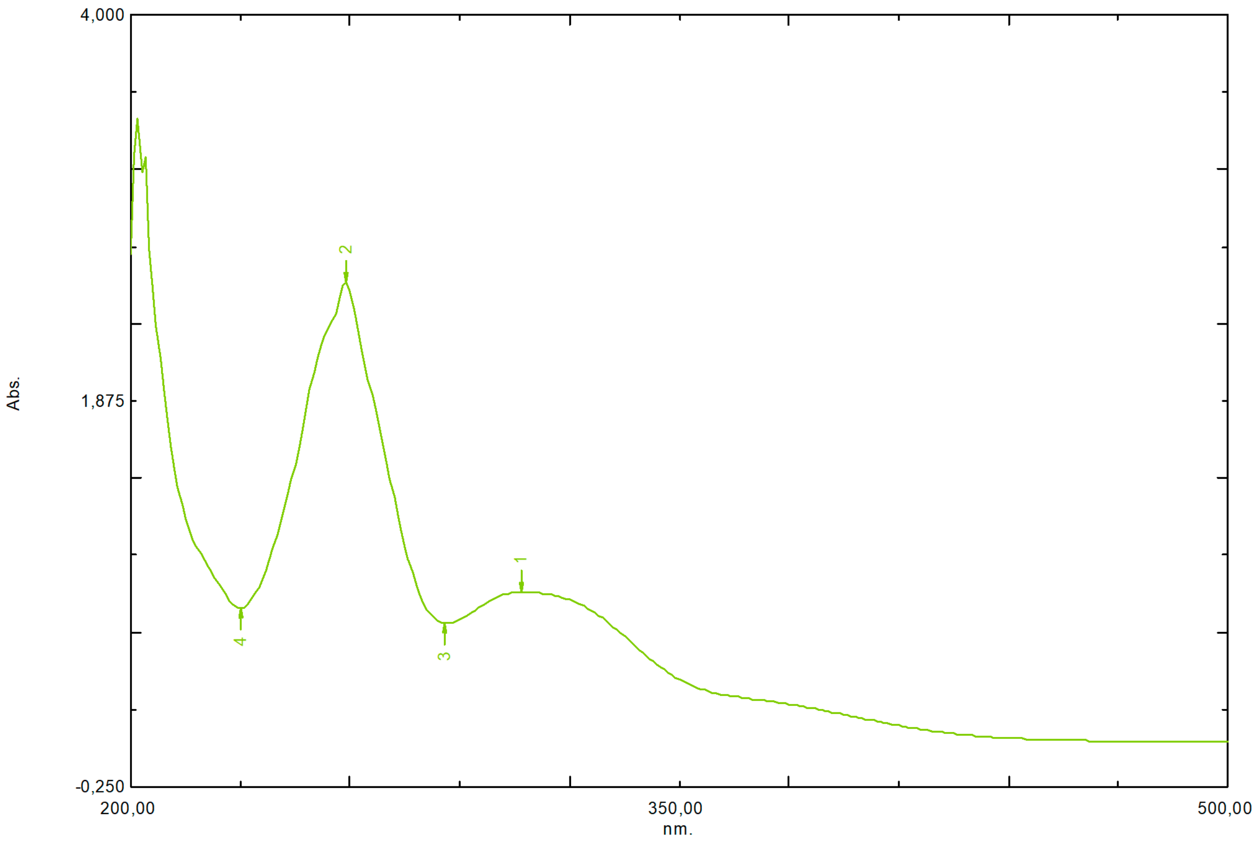

Compound *E*-**2e** (C = 1.235 $\times 10^{-4}$ M)

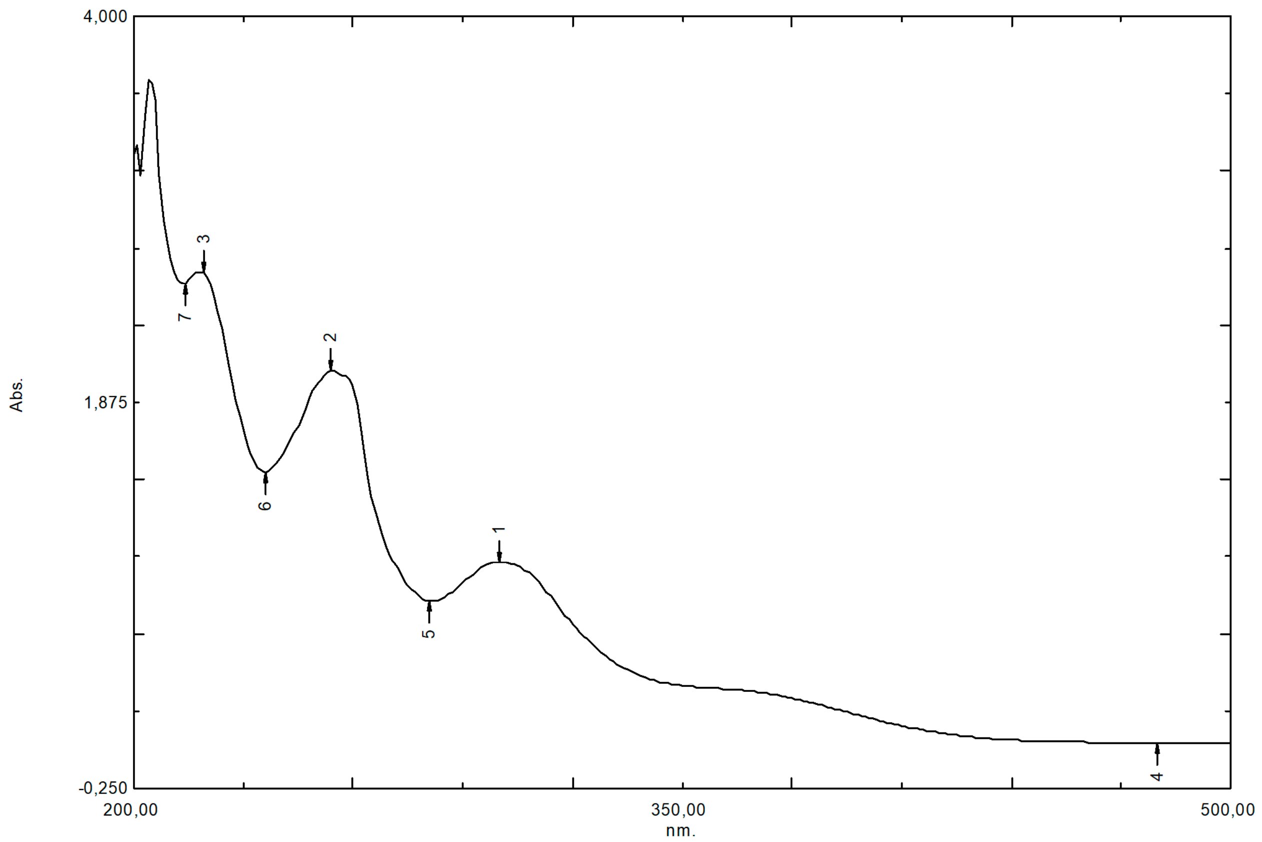

Compound *Z*-**2e** (C = 1.235 $\times 10^{-4}$ M)

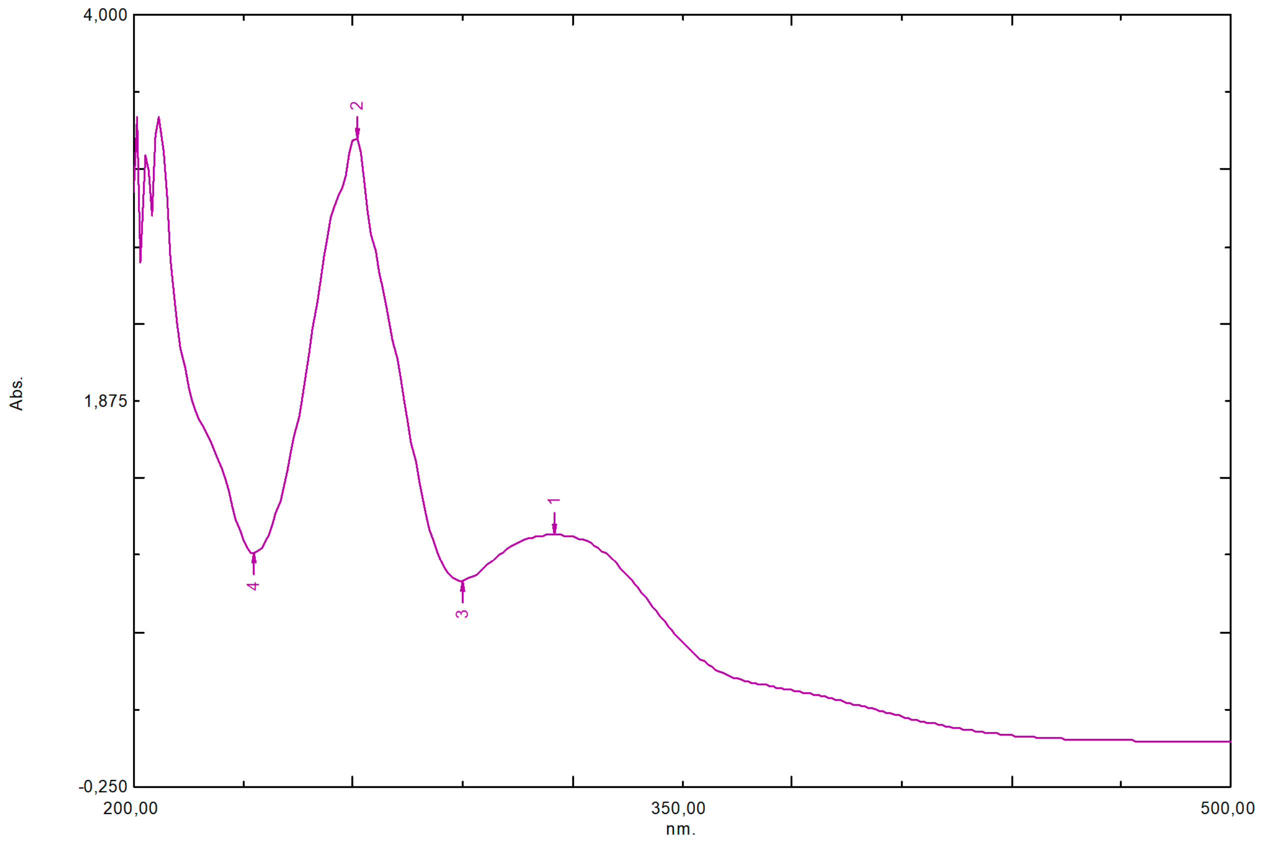

Compound *E*-**2f** (C = 1.336 $\times 10^{-4}$ M)

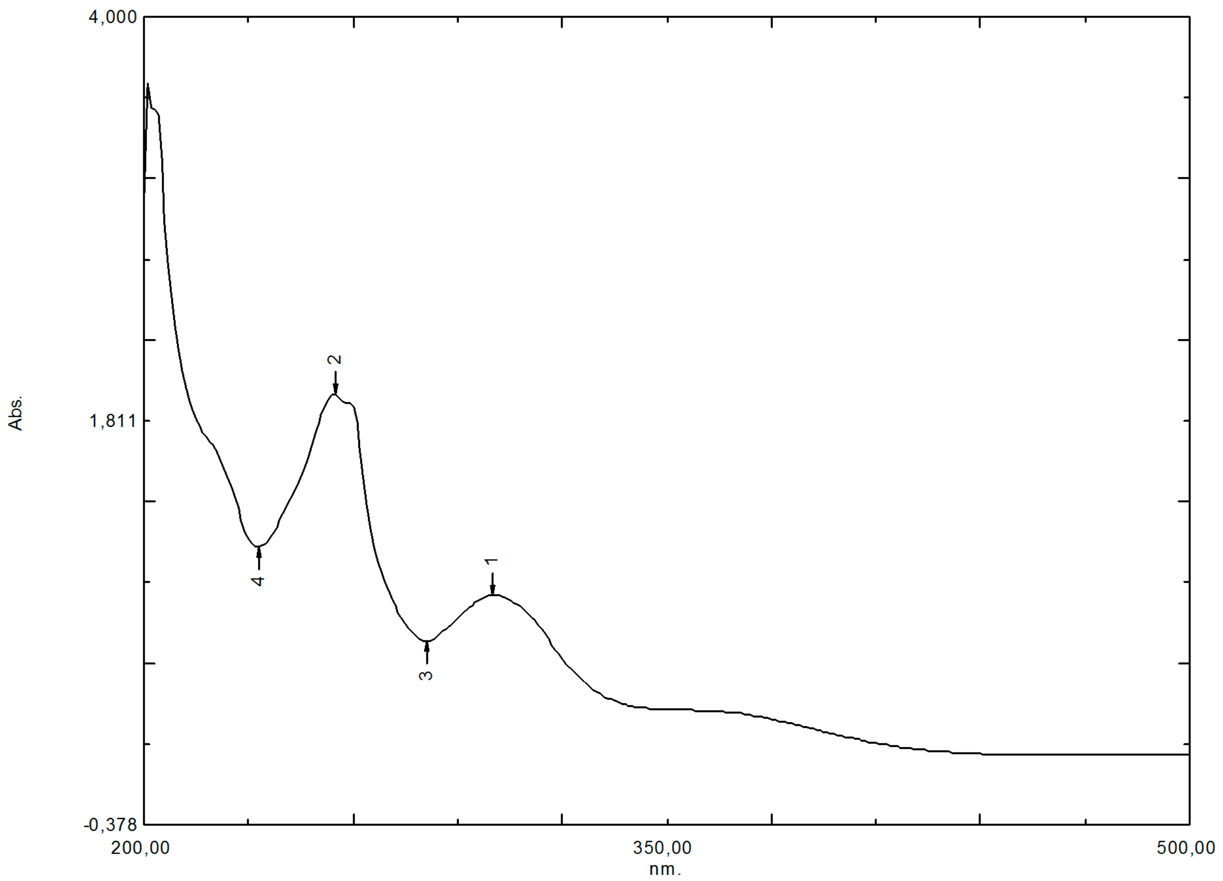

Compound *Z*-**2f** (C = 1.336 $\times 10^{-4}$ M)

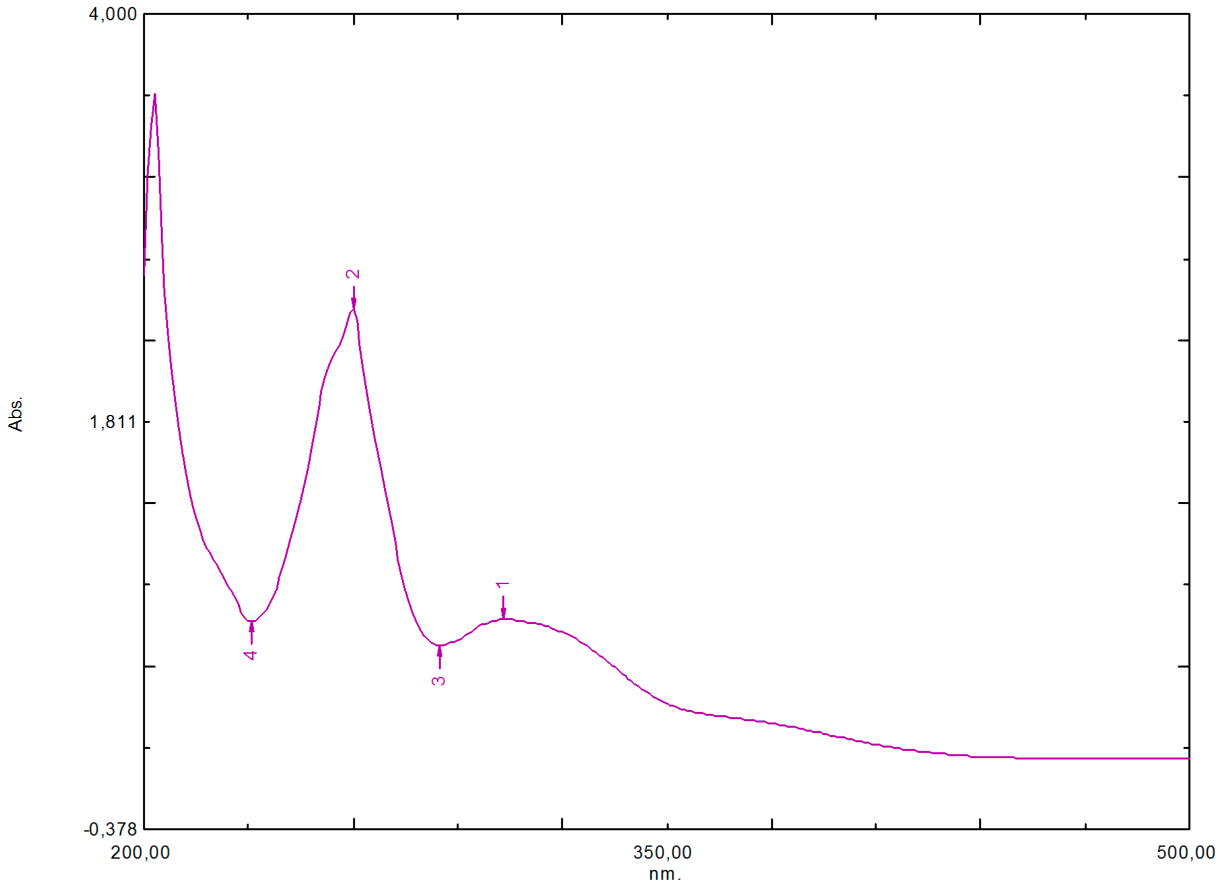

Compound *E*-**3c** (C = 1.02 ×10^{-4} M)

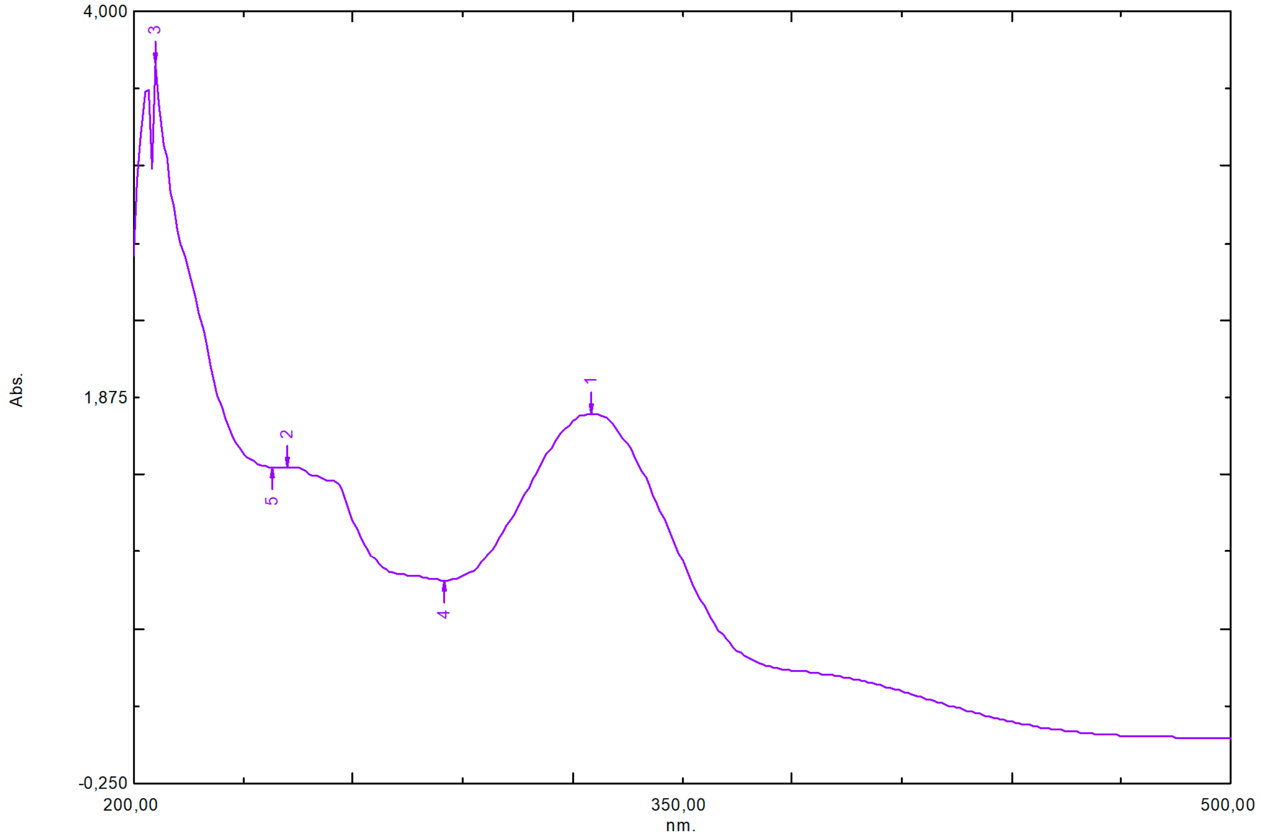

Compound *Z*-**3c** (C = 1.02 ×10^{-4} M)

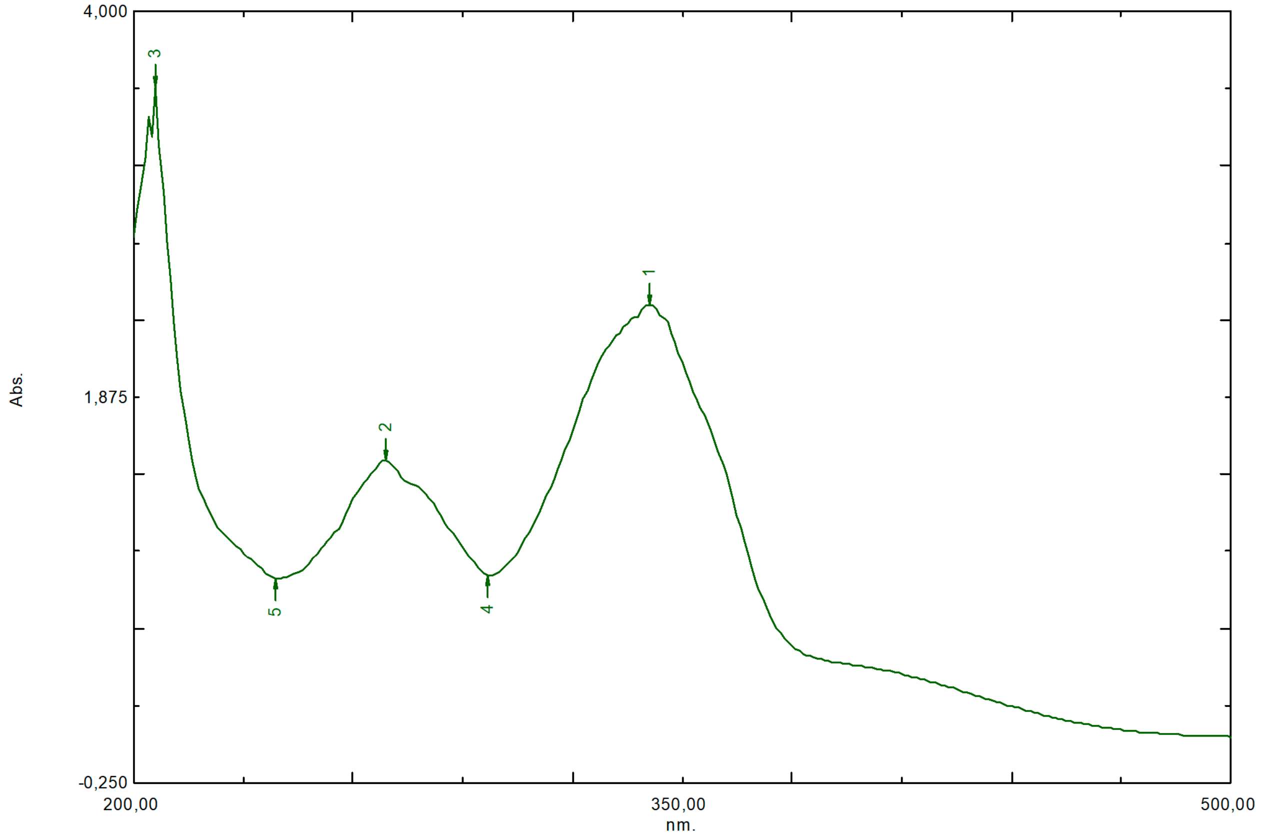

Compound *E*-**3d** (C = 1.083 $\times 10^{-4}$ M)

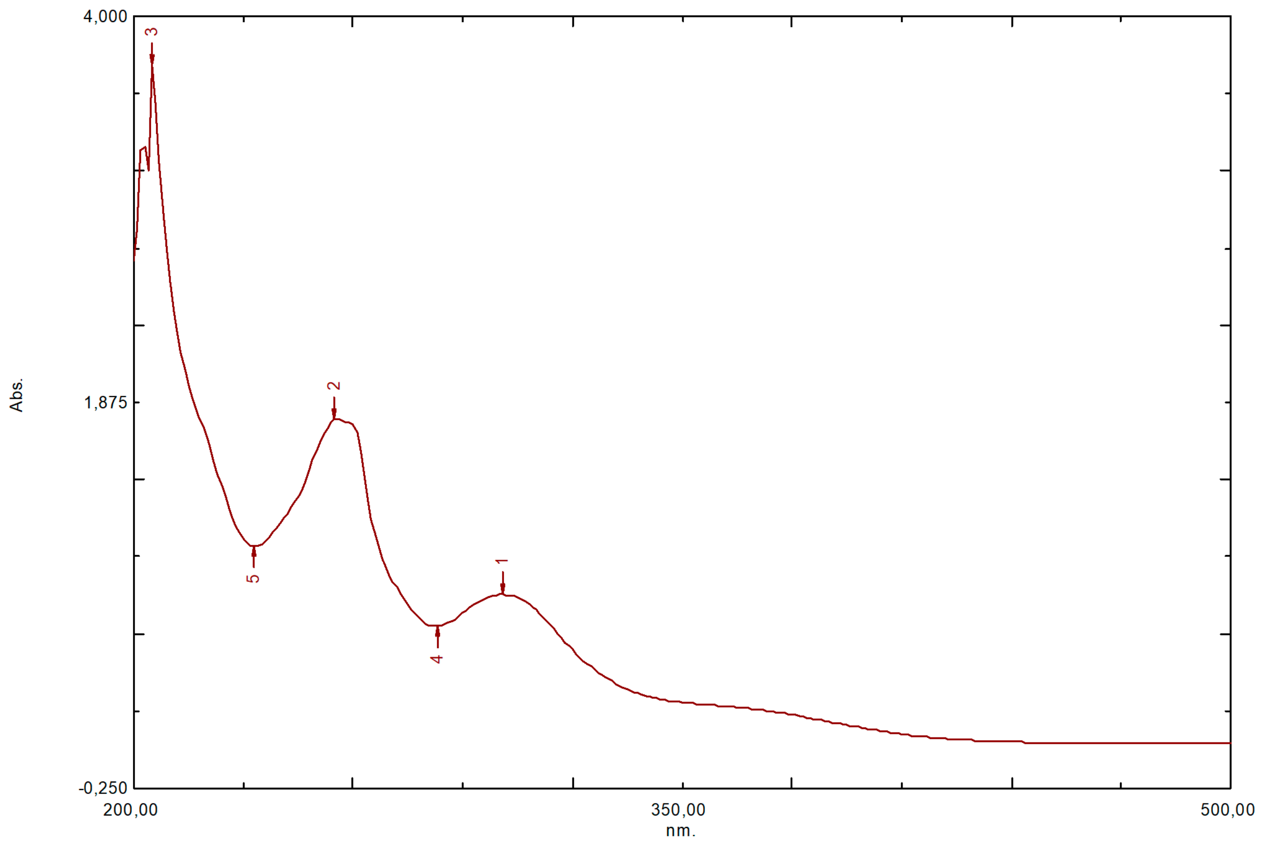

Compound *Z*-**3d** (C = 1.083 $\times 10^{-4}$ M)

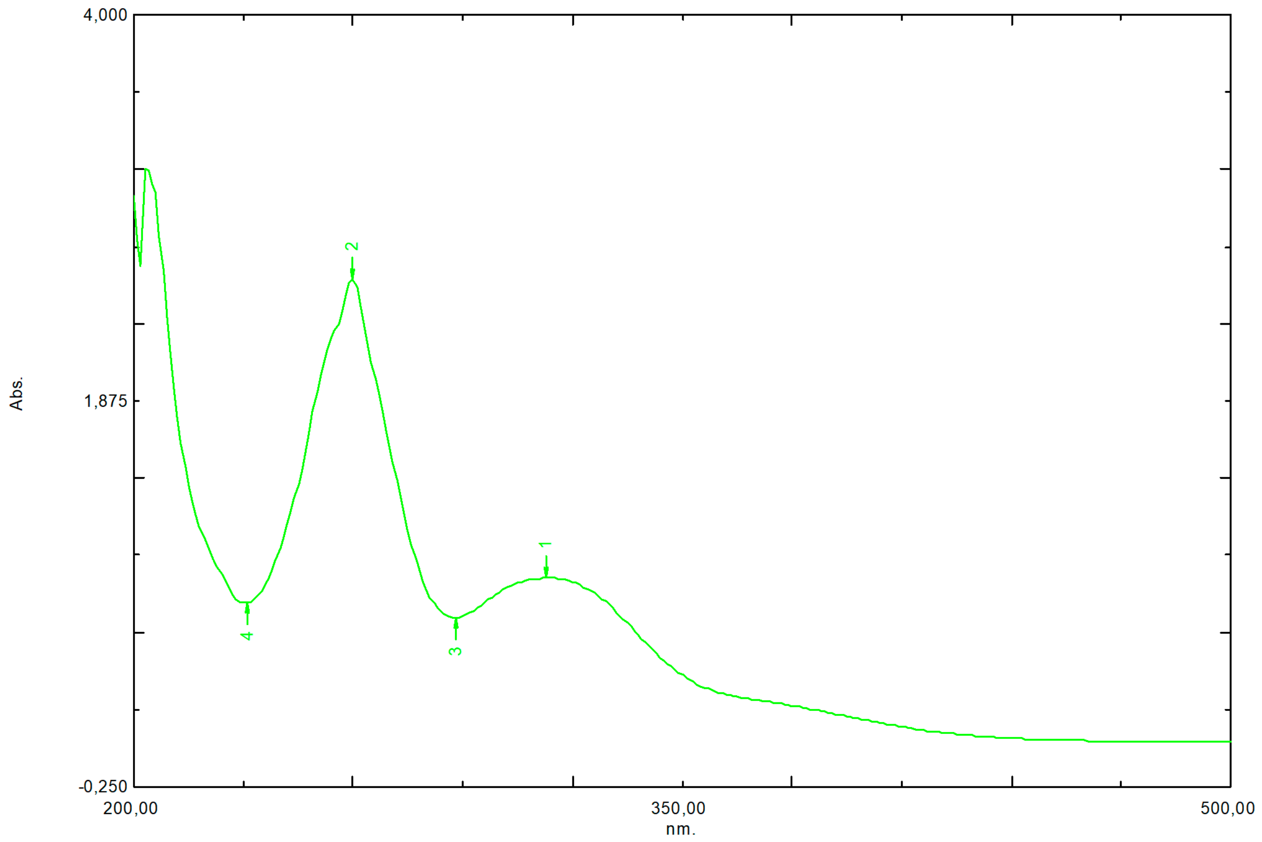

Compound *E*-**3e** (C = 9.743 ×10^{-5} M)

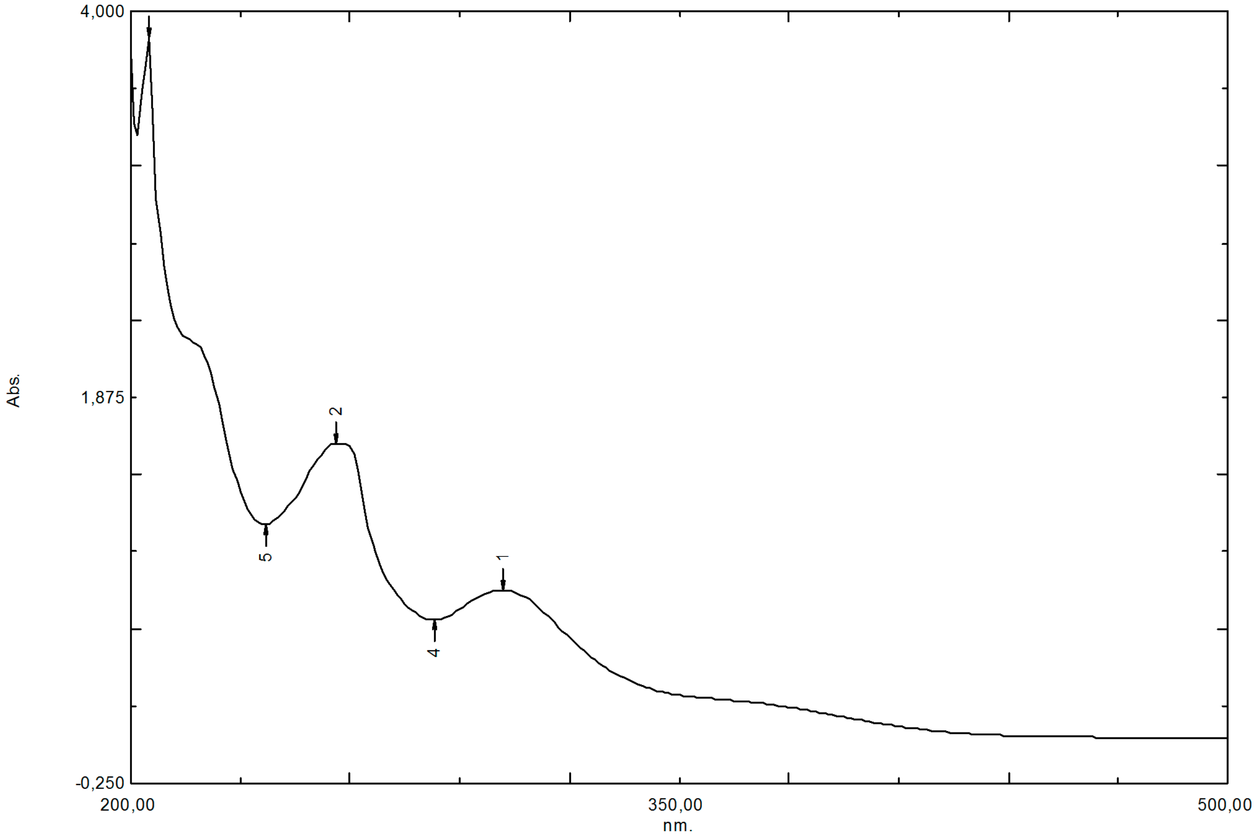

Compound *Z*-**3e** (C = 9.743 ×10^{-5} M)

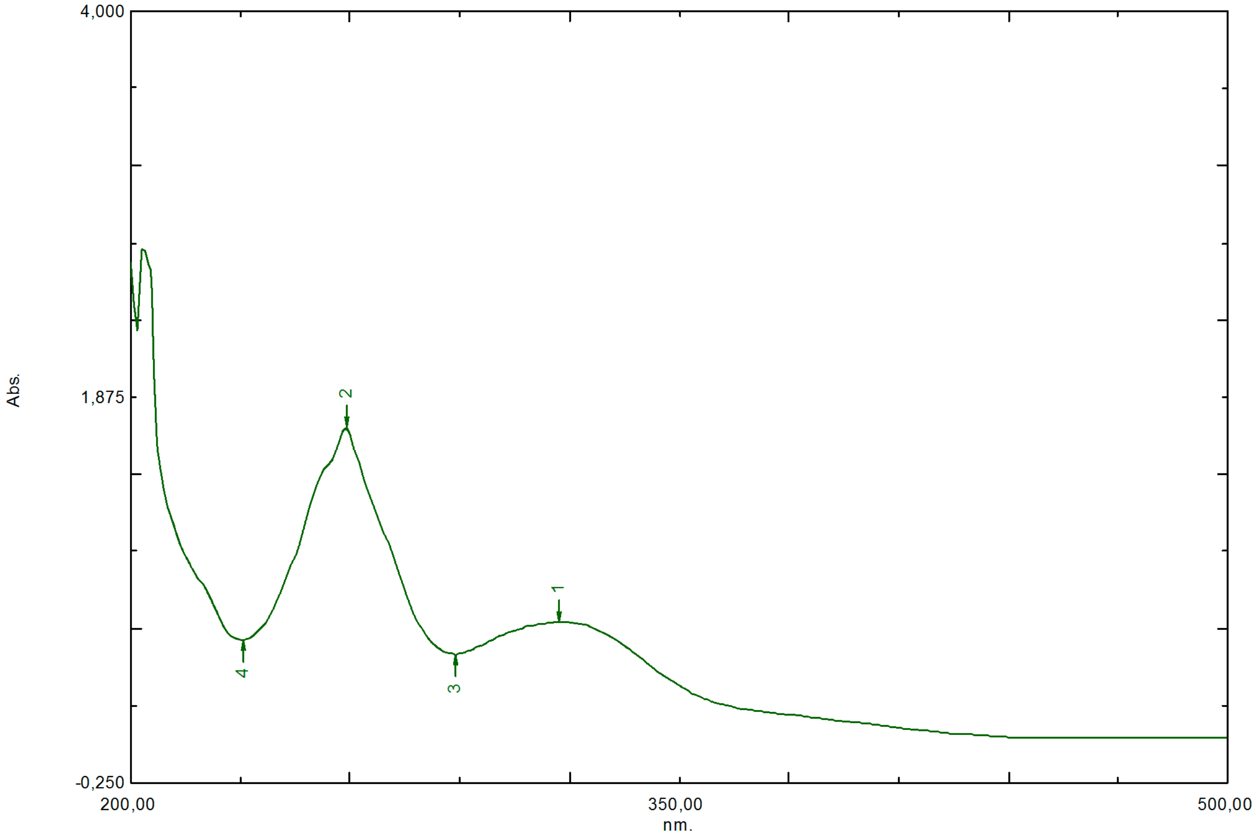

Oxindole (C = 1.252 ×10^{-4} M)

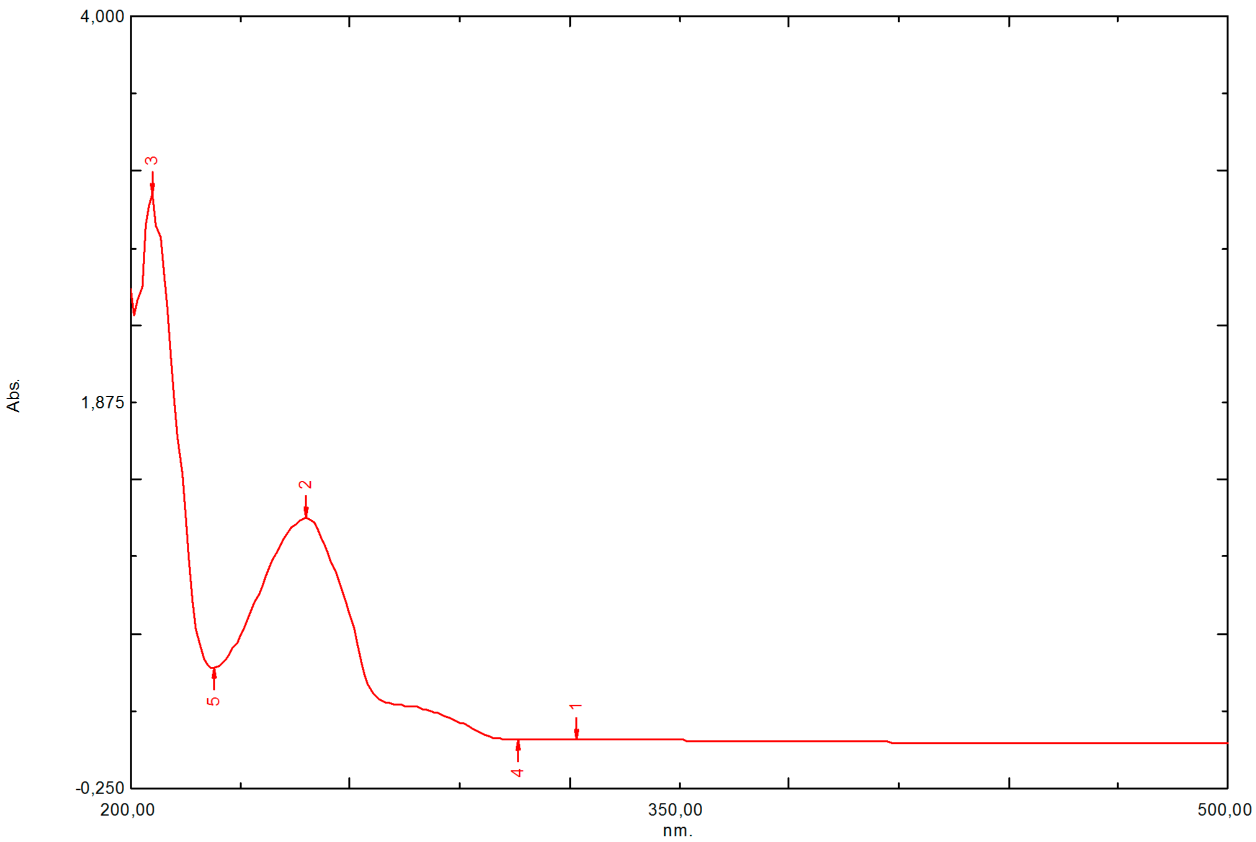

Isosbestic point for Z-**2d** → E-**2d** (C = 1.450 ×10^{-4} M)

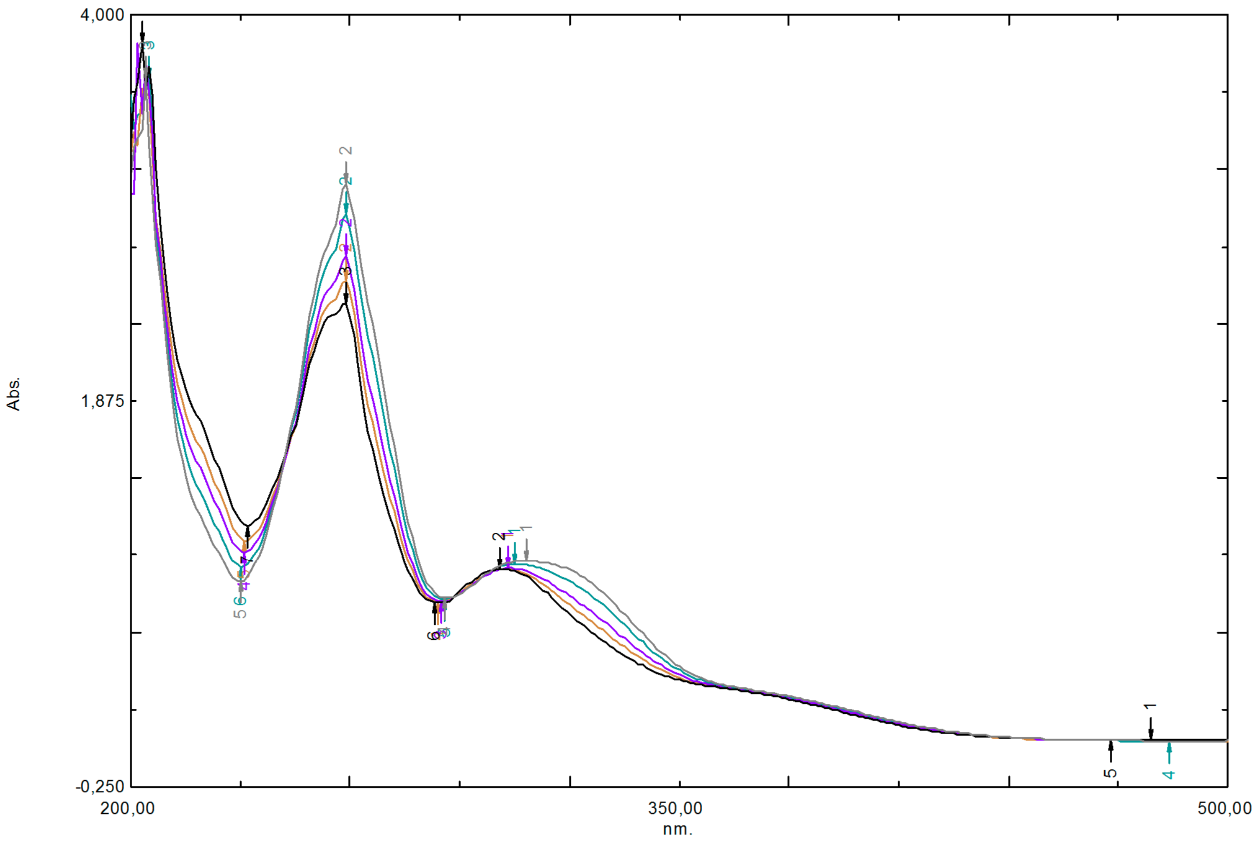

LC-MS spectra of isomeric pairs

Compound **2a**

Datafile Name:2a-2_55-45.lcd

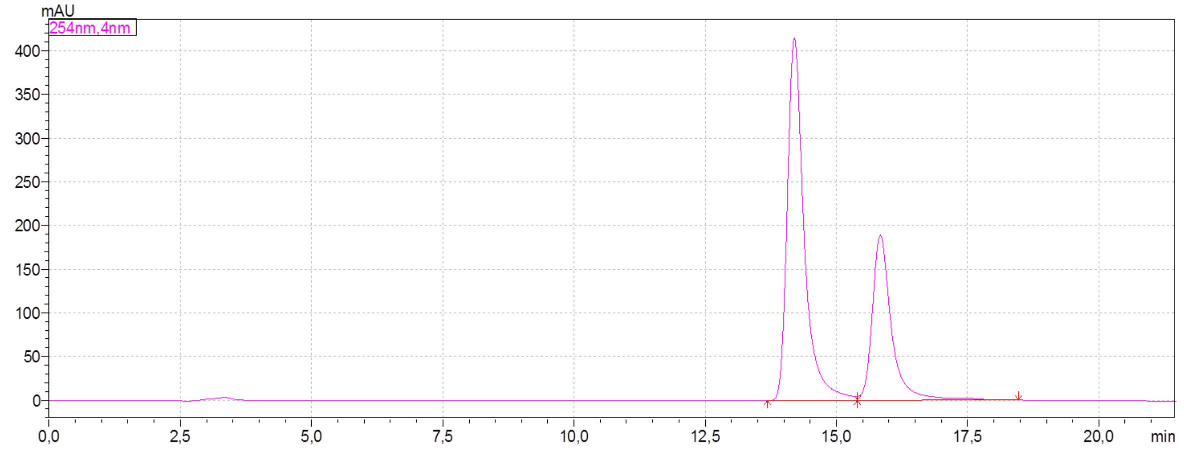

Peak#	Area%	Height%	Ret. Time	Area%	Height%
1	9295204	414782	14.197	65.823	68.702
2	4826280	188957	15.836	34.177	31.298
Total	14121483	603740		100.000	100.000

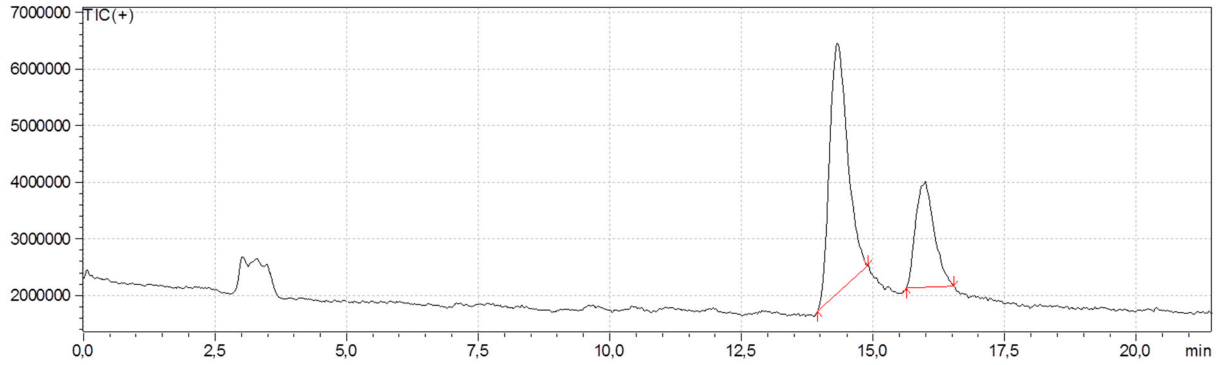

Mass spectra of the first peak (corresponds to *E*-isomer):

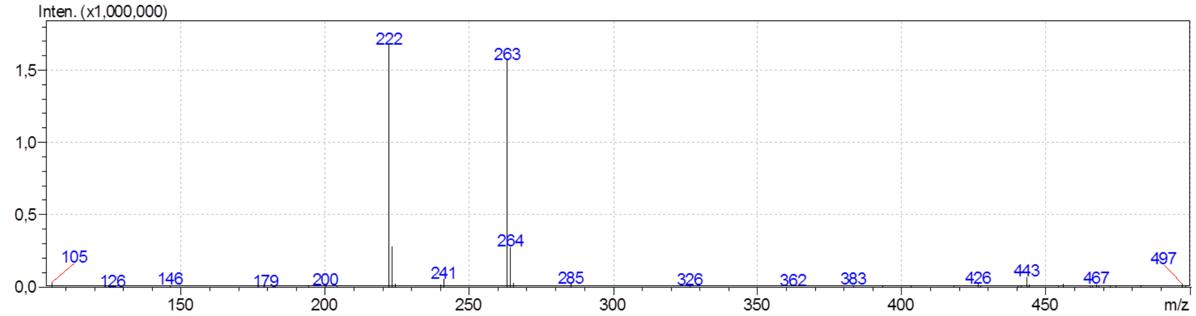

Raw Spectrum: [14.300 → 14.333]; Background: [13.950 → 14.883]; Base Peak: m/z 222.20

Peak#	m/z	Relative Intensity
1	222.20	100.00
2	263.20	93.69
3	223.20	16.83
4	264.25	16.61

Mass spectra of the second peak (corresponds to *Z* -isomer):

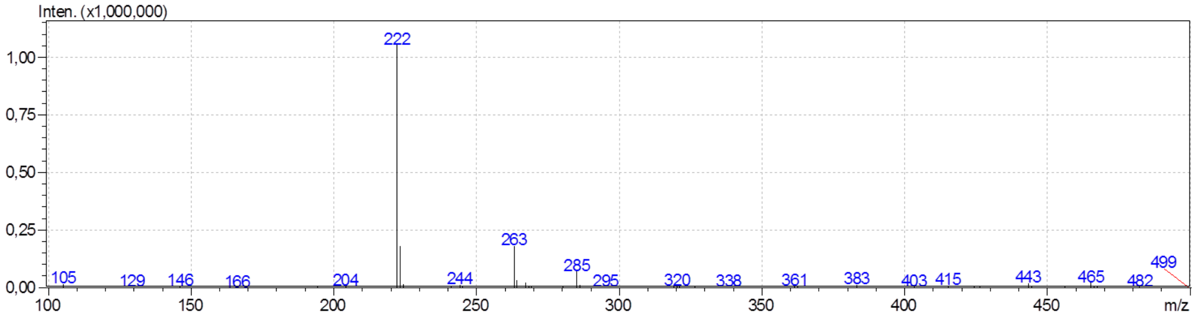

Raw Spectrum: [15.983 → 16.017]; Background: [15.650 → 16.533]; Base Peak: m/z 222.15

Peak#	m/z	Relative Intensity
1	222.15	100.00
2	263.20	17.30
3	223.15	17.01
4	285.20	6.65

Compound **2b**

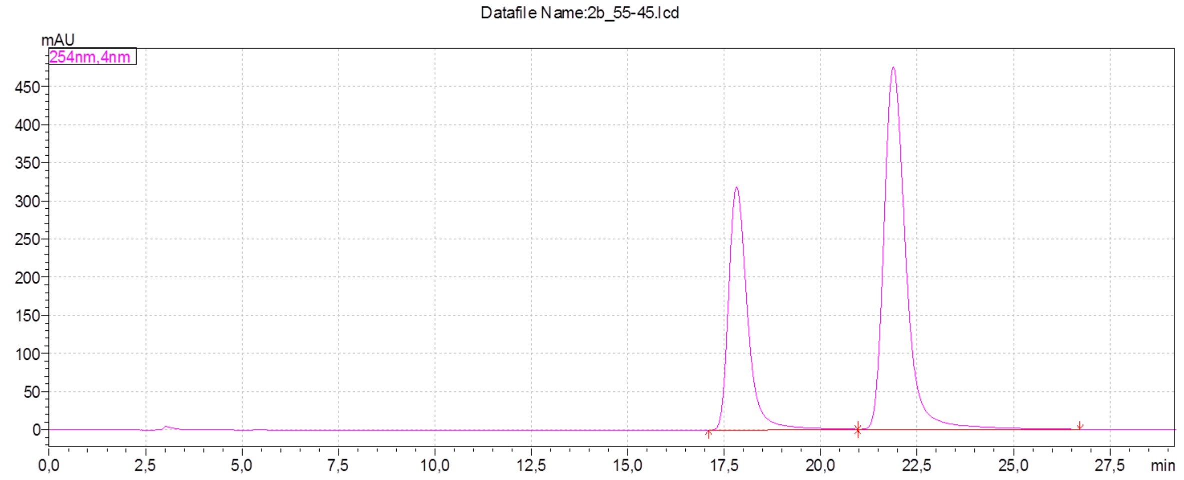

Peak#	Area%	Height%	Ret. Time	Area%	Height%
1	10872930	318528	17.820	36.751	40.130
2	18712286	475217	21.885	63.249	59.870
Total	29585216	793746		100.000	100.000

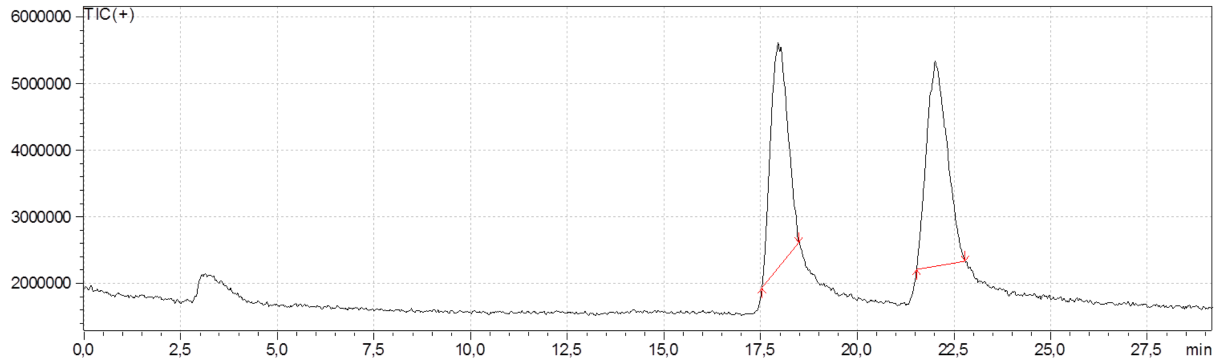

Mass spectra of the first peak (corresponds to *E*-isomer):

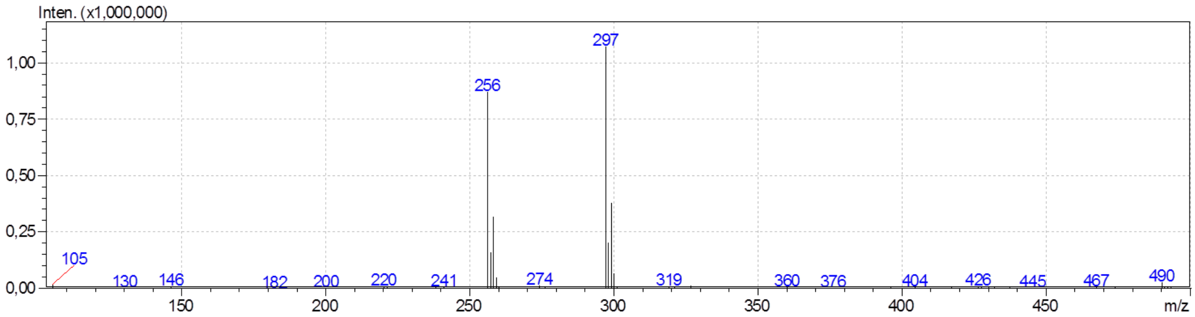

Raw Spectrum: [17.933 → 17.967]; Background: [17,533 → 18,483]; Base Peak: m/z 297.15

Peak#	m/z	Relative Intensity
1	297.15	100.00
**2**	**256.10**	**81.11**
3	299.10	35.43
4	258.10	29.54
5	298.15	18.81
6	257.15	14.66
7	300.15	5.92

Mass spectra of the second peak (corresponds to *Z*-isomer):

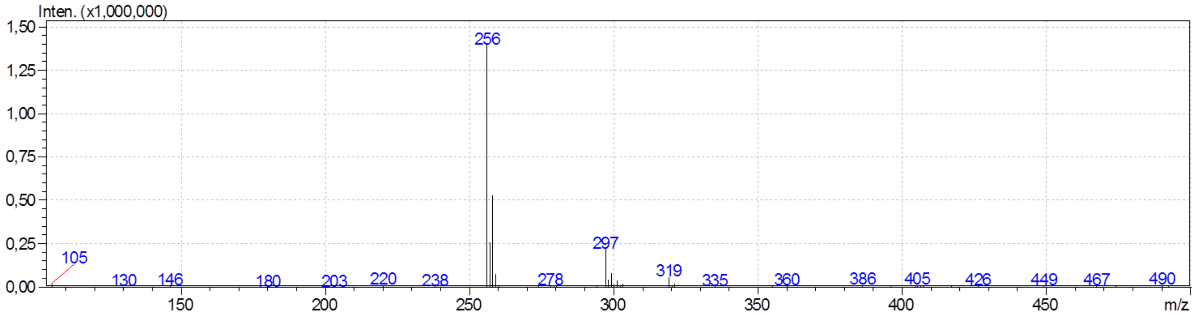

Raw Spectrum: [22.000 → 22.033]; Background: [21.533 → 22.783]; Base Peak: m/z 256.10

Peak#	m/z	Relative Intensity
1	256.10	100.00
2	258.10	37.78
3	257.15	18.39
4	297.15	15.64
5	299.10	5.85
6	259.15	5.62

Compound **2c**

Datafile Name:2c-2_55-45.lcd

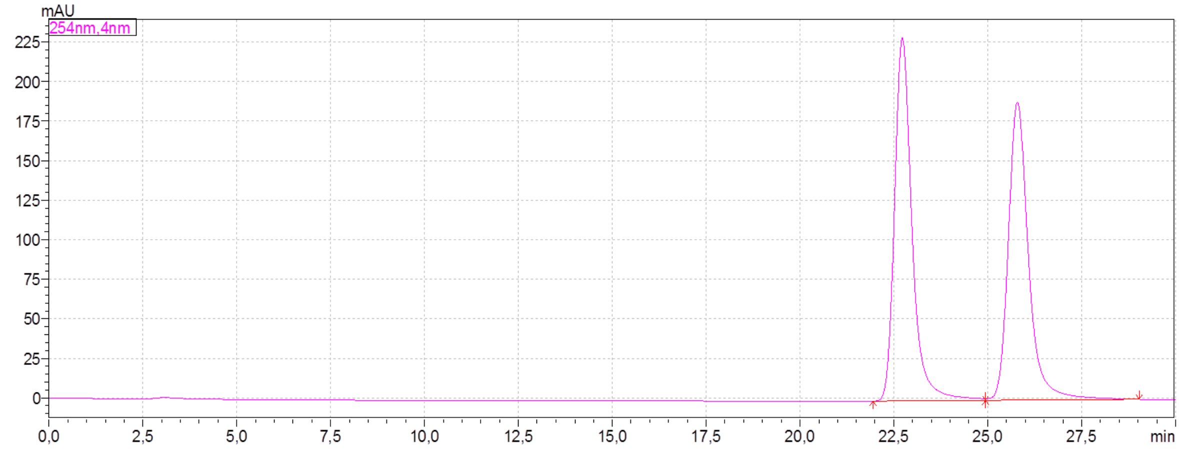

Peak#	Area%	Height%	Ret. Time	Area%	Height%
1	7435966	229488	22.724	51.311	54.926
2	7055932	188328	25.790	48.689	45.074
Total	14491898	417816		100.000	100.000

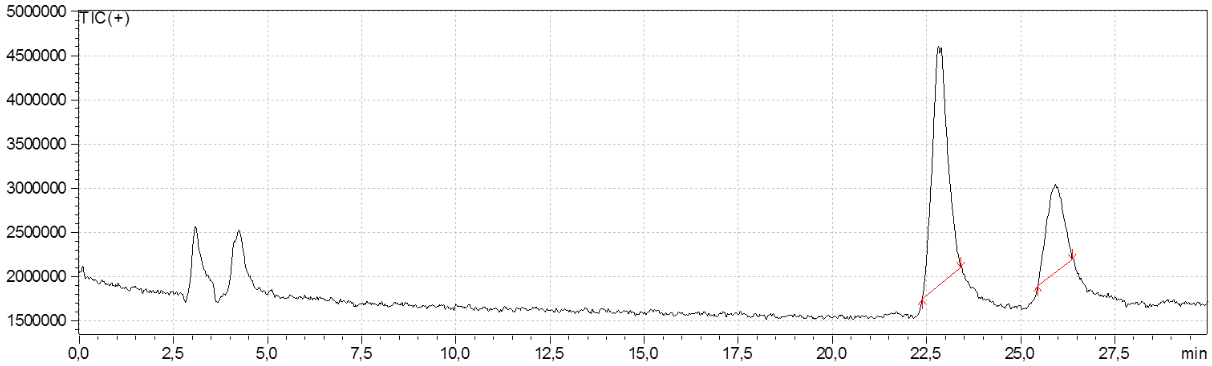

Mass spectra of the first peak (corresponds to *E*-isomer):

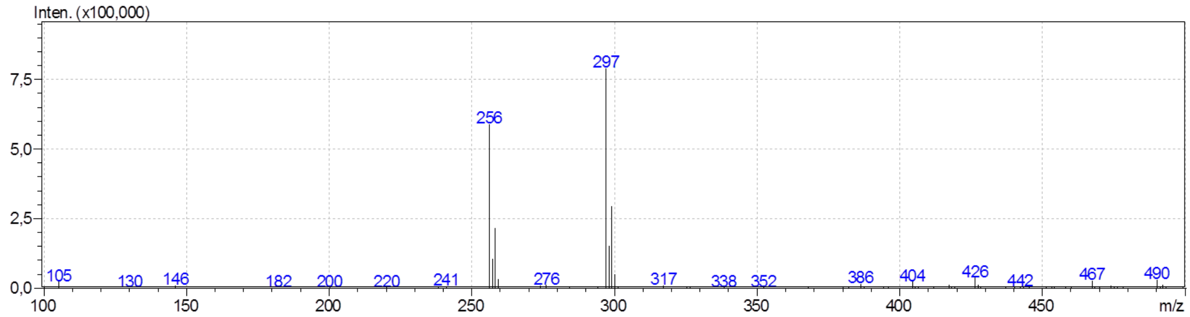

Raw Spectrum: [22.800 → 22.833]; Background: [22.383 → 23.400]; Base Peak: m/z 297.15

Peak#	m/z	Relative Intensity
1	297.15	100.00
2	**256.15**	**74.44**
3	299.10	37.26
4	258.10	27.30
5	298.15	19.26
6	257.15	13.38
7	300.15	6.27

Mass spectra of the second peak (corresponds to *Z*-isomer):

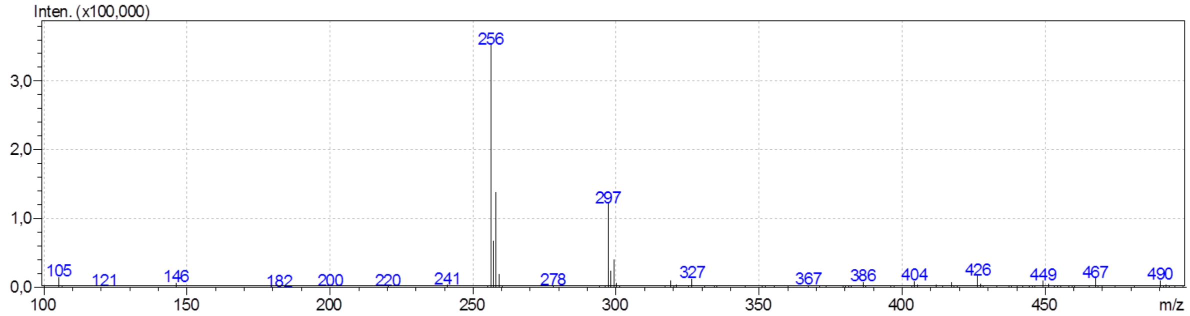

Raw Spectrum: [25.900 → 25.933]; Background: [25.450 → 26.367]; Base Peak: m/z 256.15

Peak#	m/z	Relative Intensity
1	256.15	100.00
2	258.10	39.29
3	297.15	34.45
4	257.15	19.19
5	299.15	11.58
6	298.20	6.79
7	259.15	5.54

Compound **2d**

Datafile Name:2d-3_55-45.lcd

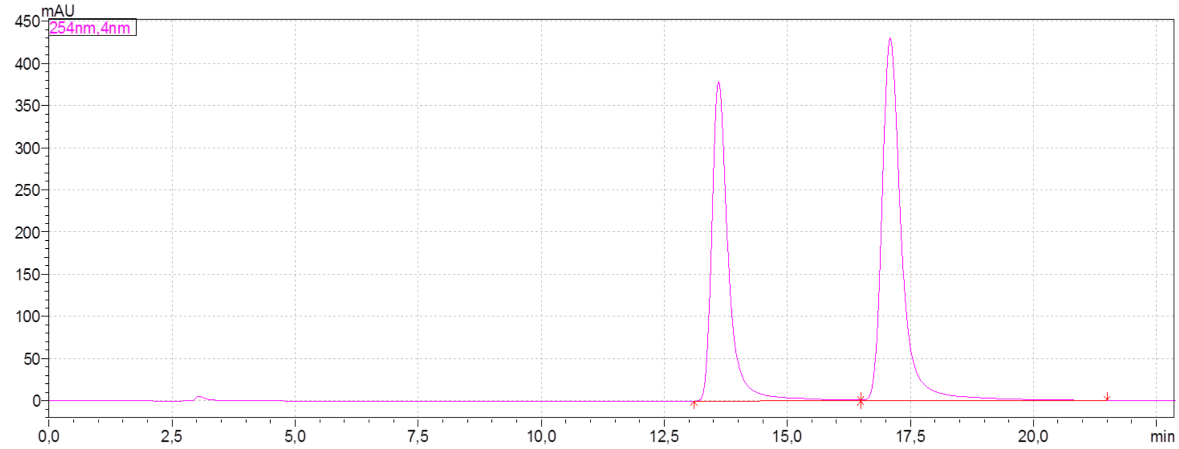

Peak#	Area%	Height%	Ret. Time	Area%	Height%
1	8875546	377970	13.604	42.786	46.777
2	11868636	430047	17.090	57.214	53.223
Total	20744182	808018		100.000	100.000

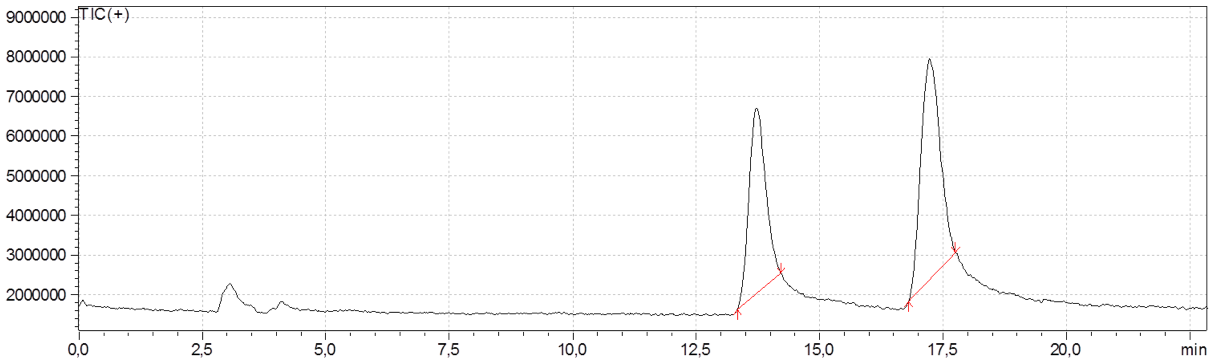

Mass spectra of the first peak (corresponds to *Z*-isomer):

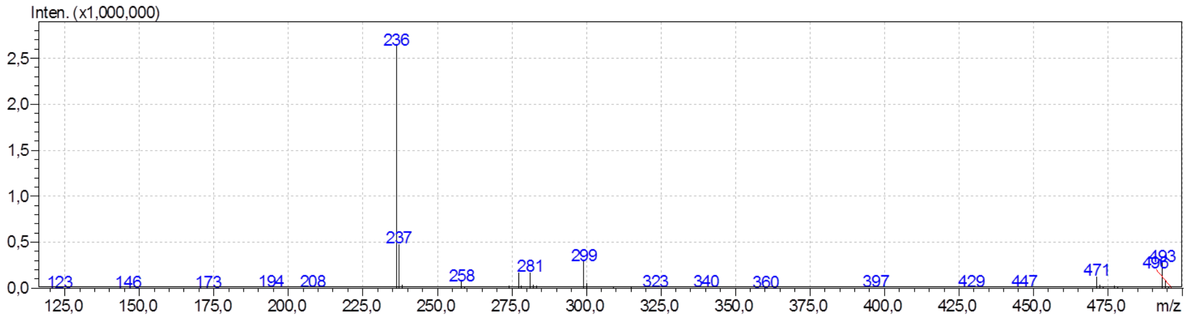

Raw Spectrum: [13.700 → 13.733]; Background: [13.333 → 14.217]; Base Peak: m/z 236.20

Peak#	m/z	Relative Intensity
1	236.20	100.00
2	237.15	18.26
3	299.20	10.94
4	493.30	10.49
5	281.20	6.54
6	277.20	6.42

Mass spectra of the second peak (corresponds to *E*-isomer):

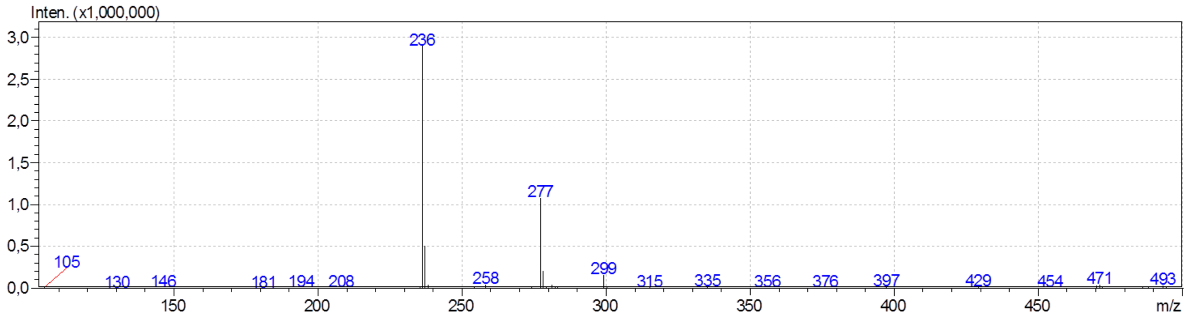

Raw Spectrum: [17.200 → 17.233]; Background: [16.800 → 17.750]; Base Peak: m/z 236.15

Peak#	m/z	Relative Intensity
1	236.15	100.00
2	277.20	37.37
3	237.15	17.64
4	278.20	6.93
5	299.20	5.68

Compound **2e**

Datafile Name:2e_55-45.lcd

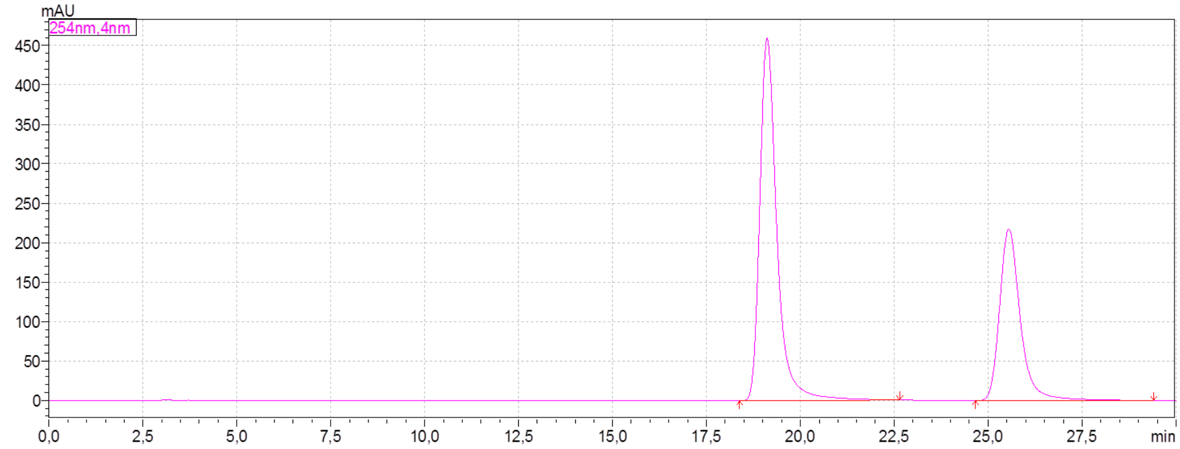

Peak#	Area%	Height%	Ret. Time	Area%	Height%
1	14572121	459971	19.116	63.540	67.879
2	8361540	217666	25.549	36.460	32.121
Total	22933662	677637		100.000	100.000

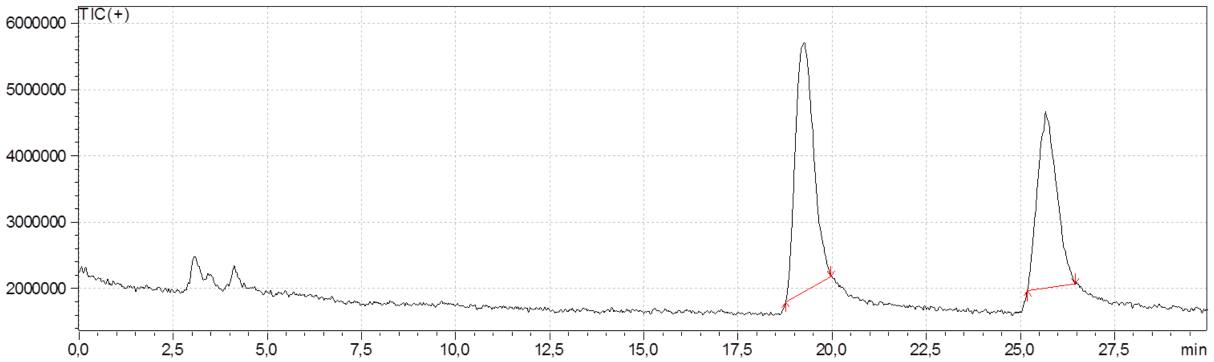

Mass spectra of the first peak (corresponds to *Z*-isomer):

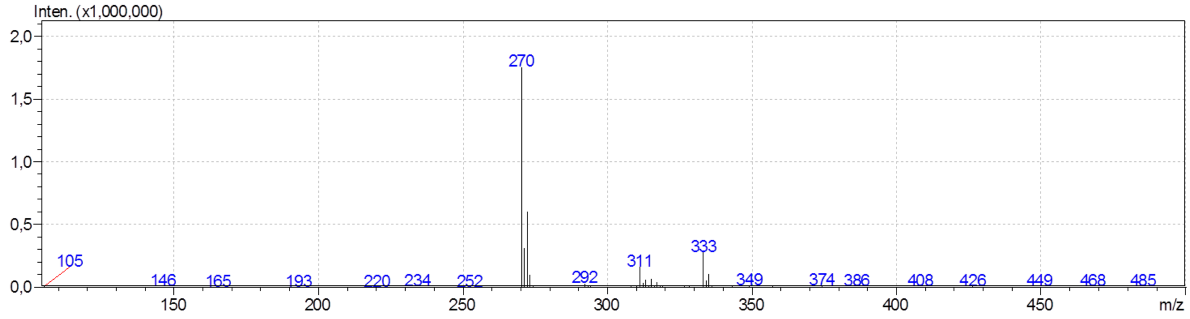

Raw Spectrum: [19.250 → 19.283]; Background: [18.767 → 19.967]; Base Peak: m/z 270.15

Peak#	m/z	Relative Intensity
1	270.15	100.00
2	272.10	34.36
3	271.15	17.69
4	333.15	15.82
5	311.15	9.24
6	335.10	5.73
7	273.15	5.64

Mass spectra of the second peak (corresponds to *E*-isomer):

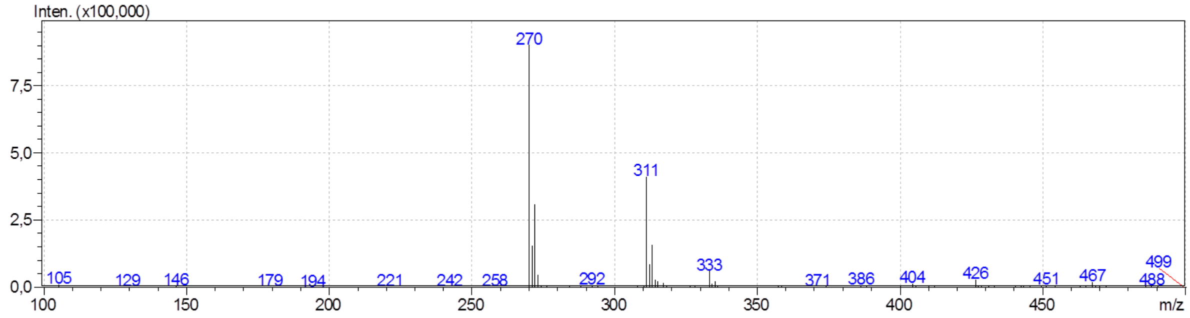

Raw Spectrum: [25.650 → 25.683]; Background: [25.183 → 26.450]; Base Peak: m/z 270.15

Peak#	m/z	Relative Intensity
1	270.15	100.00
2	311.15	45.81
3	272.10	34.39
4	313.10	17.55
5	271.15	17.37
6	312.15	9.50
7	333.15	6.68
8	273.15	5.26

Compound **2f**

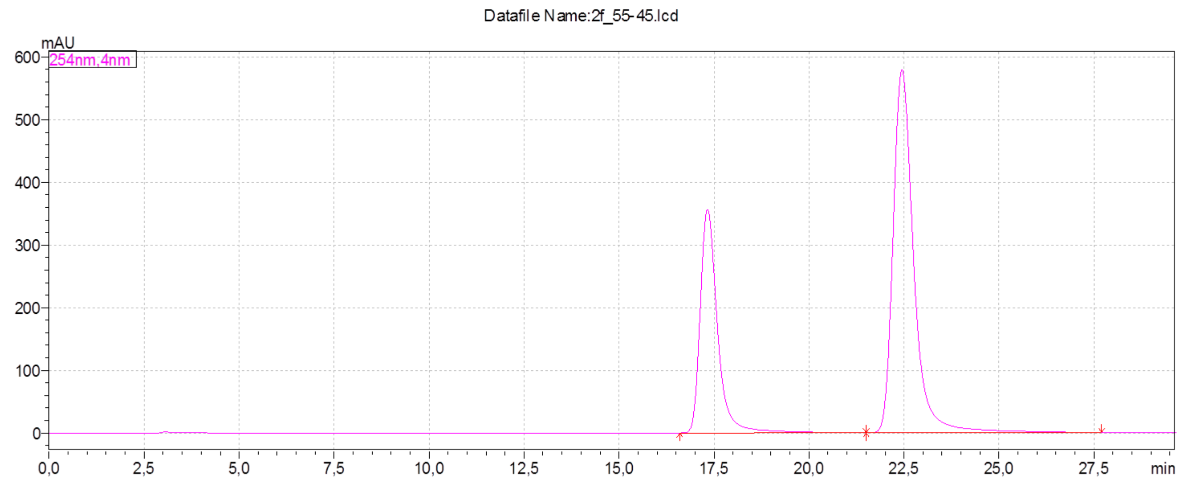

Peak#	Area%	Height%	Ret. Time	Area%	Height%
1	11325838	357026	17.329	34.147	38.149
2	21842438	578847	22.447	65.853	61.851
Total	33168276	935872		100.000	100.000

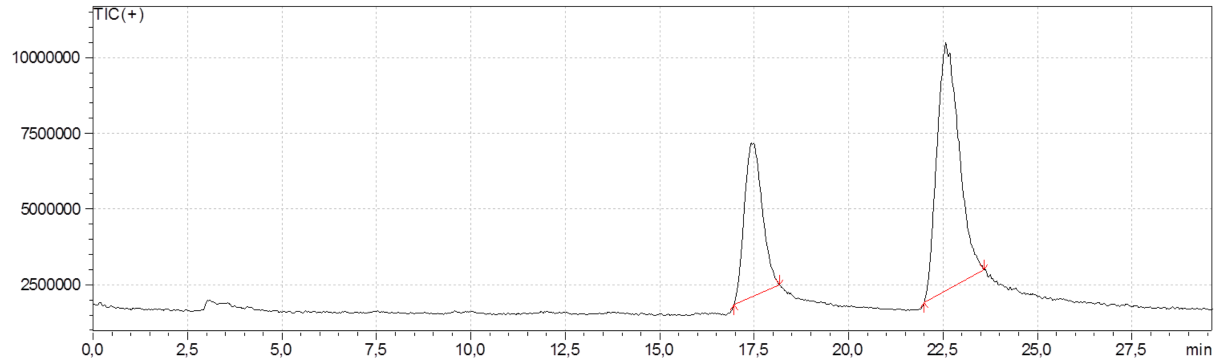

Mass spectra of the first peak (corresponds to *Z*-isomer):

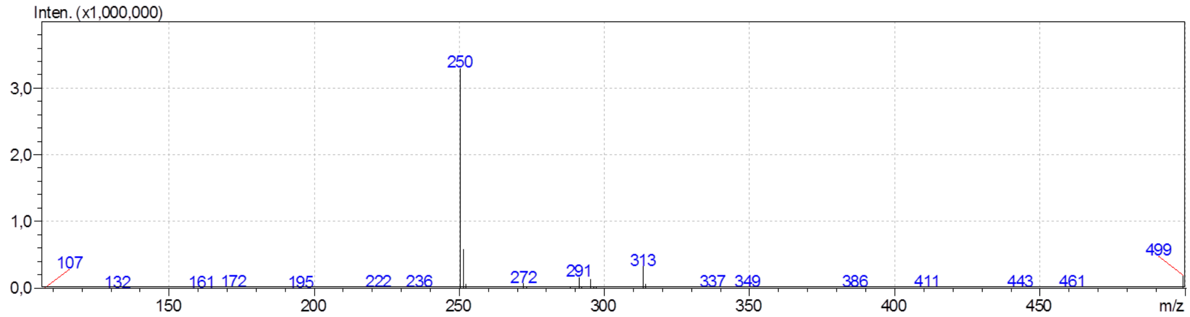

Raw Spectrum: [17.400 → 17.433]; Background: [16.967 → 18.167]; Base Peak: m/z 250.20

Peak#	m/z	Relative Intensity
1	250.20	100.00
2	251.25	17.81
3	313.20	9.90
4	499.35	5.84

Mass spectra of the second peak (corresponds to *E*-isomer):

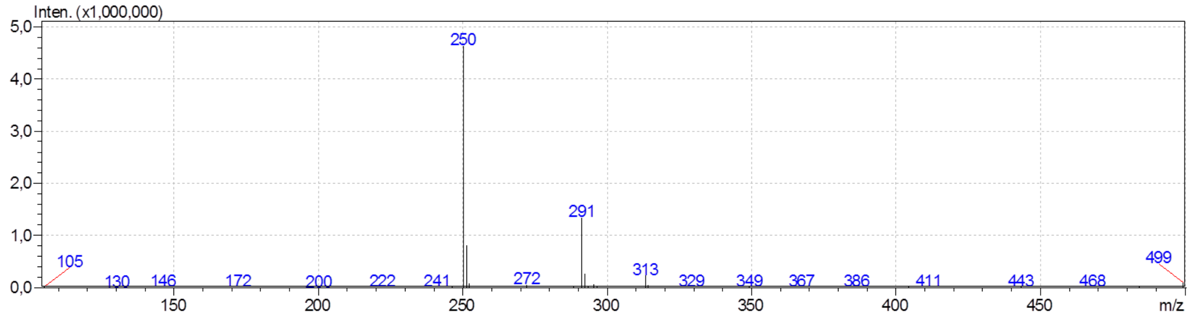

Raw Spectrum: [22.550 → 22.583]; Background: [22.000 → 23.583]; Base Peak: m/z 250.20

Peak#	m/z	Relative Intensity
1	250.20	100.00
2	291.20	28.97
3	251.25	17.47
4	292.25	5.90

Compound **3c**

Datafile Name:3c_55-45.lcd

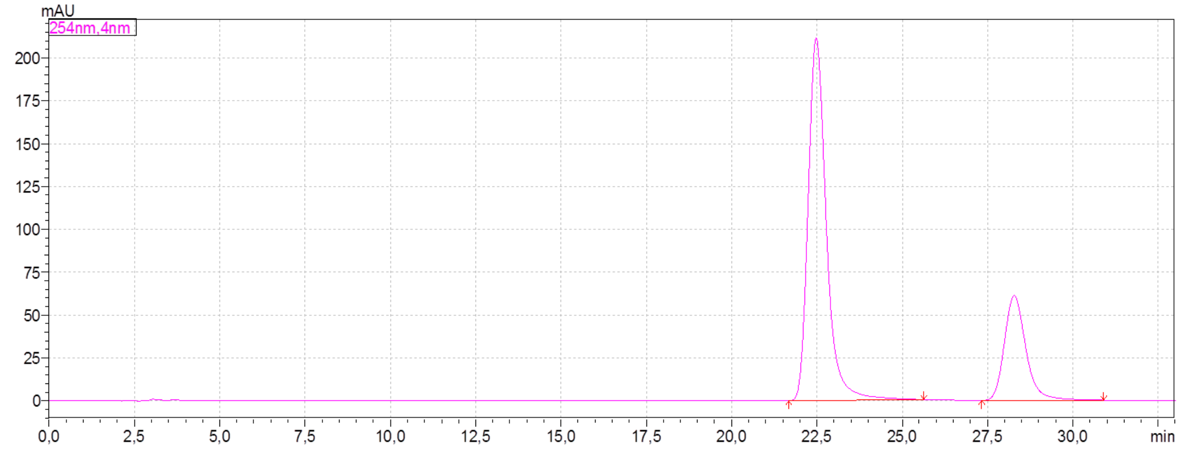

Peak#	Area%	Height%	Ret. Time	Area%	Height%
1	7823843	211404	22.481	74.605	77.577
2	2663130	61106	28.281	25.395	22.423
Total	10486973	272510		100.000	100.000

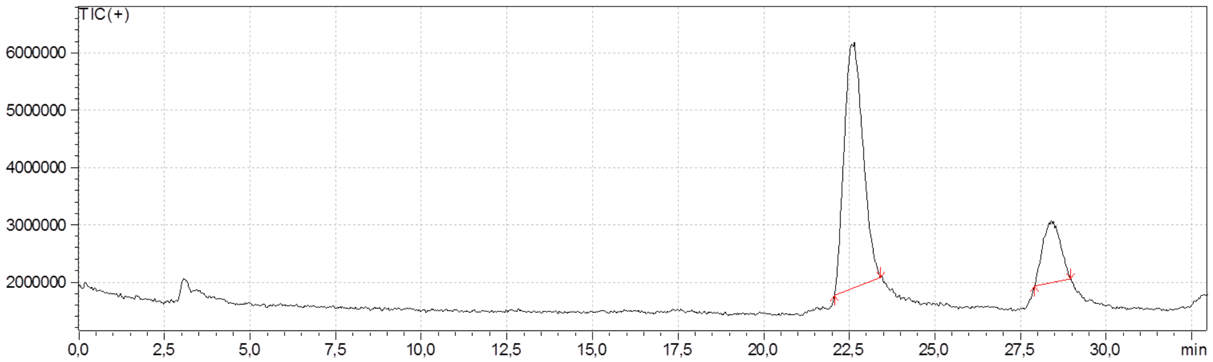

Mass spectra of the first peak (corresponds to *E*-isomer):

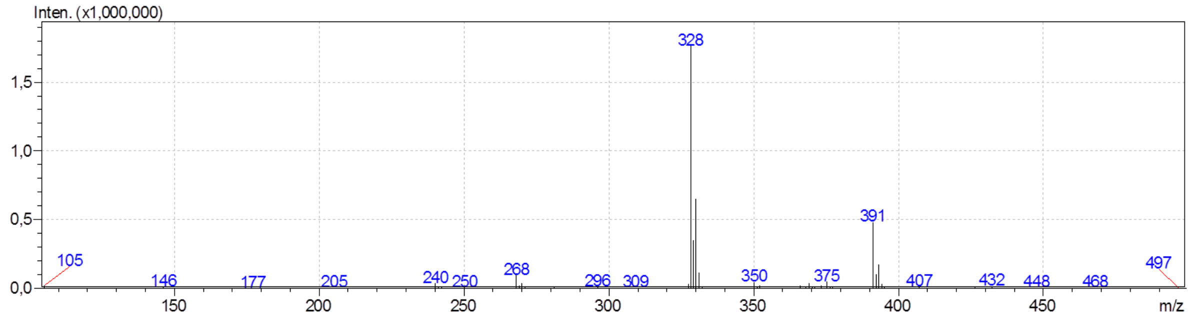

Raw Spectrum: [22.633 → 22.667]; Background: [22.067 → 23.417]; Base Peak: m/z 328.15

Peak#	m/z	Relative Intensity
1	328.15	100.00
2	330.10	37.06
3	391.15	27.25
4	329.15	19.77
5	393.15	9.76
6	331.15	6.48
7	392.25	5.94
8	268.10	5.26

Mass spectra of the second peak (corresponds to *Z*-isomer):

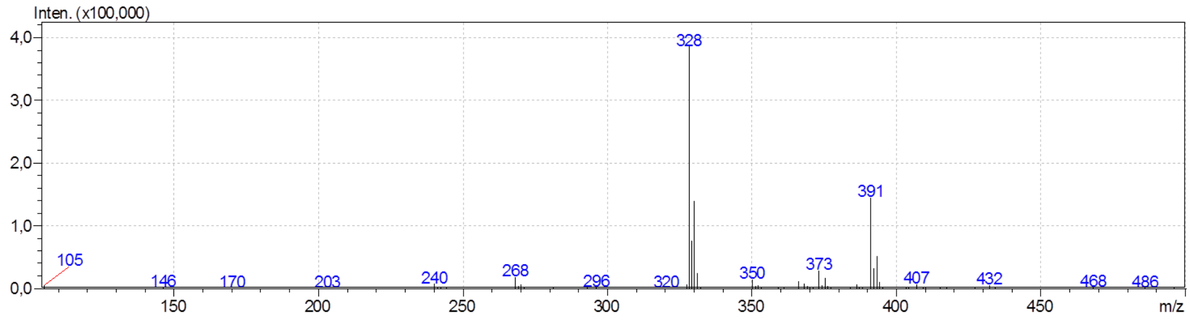

Raw Spectrum: [28.383 → 28.417]; Background: [27.917 → 28.967]; Base Peak: m/z 328.15

Peak#	m/z	Relative Intensity
1	328.15	100.00
2	391.15	37.57
3	330.10	36.28
4	329.15	19.97
5	393.15	13.42
6	392.25	8.29
7	373.20	7.41
8	331.15	6.51

Compound **3d**

Datafile Name:3d-3_55-45.lcd

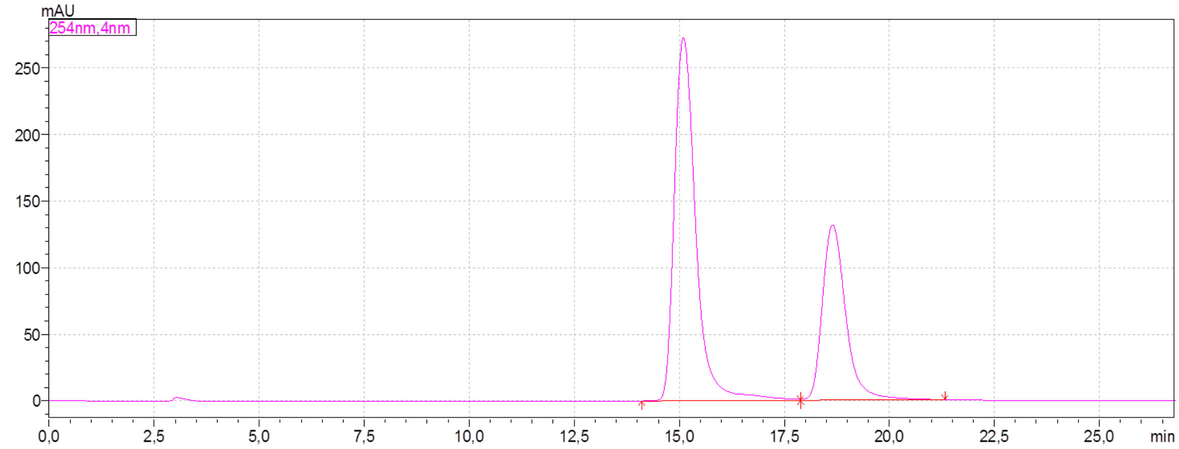

Peak#	Area%	Height%	Ret. Time	Area%	Height%
1	9659332	272516	15.098	65.571	67.443
2	5071744	131552	18.650	34.429	32.557
Total	14731076	404068		100.000	100.000

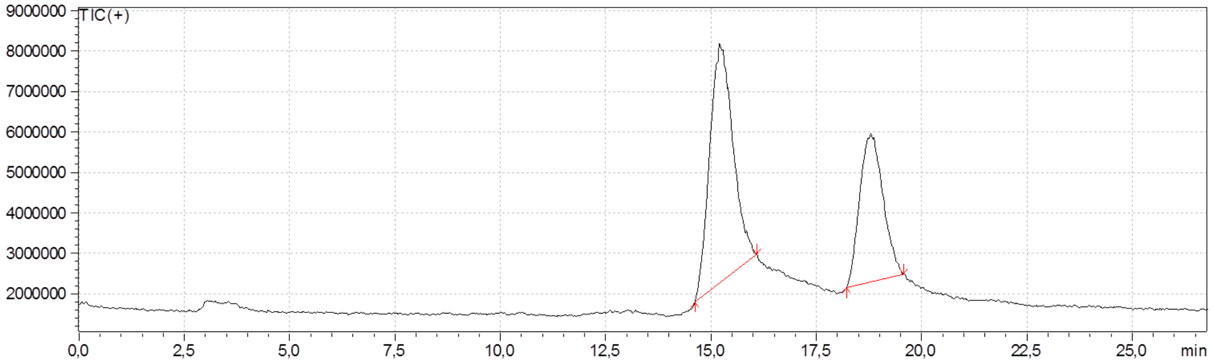

Mass spectra of the first peak (corresponds to *Z*-isomer):

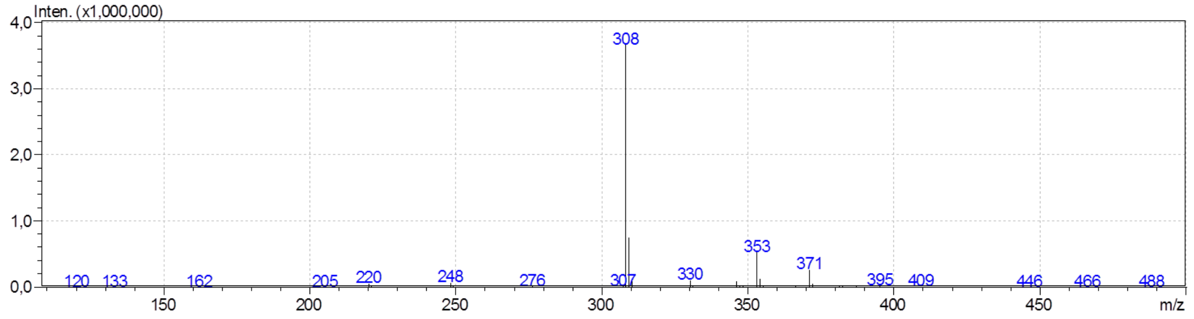

Raw Spectrum: [15.183 → 15.217]; Background: [14.617 → 16.083]; Base Peak: m/z 308.20

Peak#	m/z	Relative Intensity
1	308.20	100.00
2	309.15	20.66
3	353.20	14.30
4	371.20	7.17

Mass spectra of the second peak (corresponds to *E*-isomer):

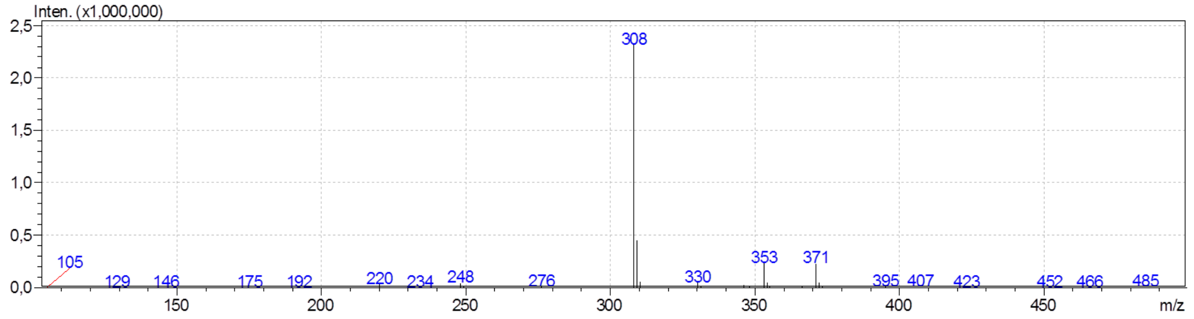

Raw Spectrum: [18.783 → 18.817]; Background: [18.217 → 19.567]; Base Peak: m/z 308.15

Peak#	m/z	Relative Intensity
1	308,15	100,00
2	309,15	19,73
3	353,25	9,95
4	371,20	9,92

Compound **3e**

Datafile Name:3e-2_55-45.lcd

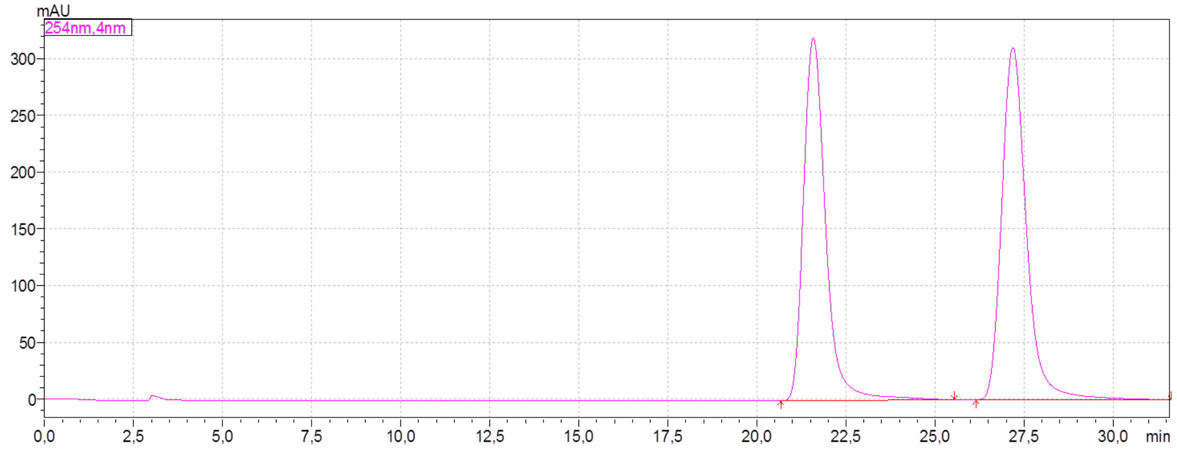

Peak#	Area%	Height%	Ret. Time	Area%	Height%
1	13788725	319811	21.579	48.020	50.718
2	14925964	310752	27.183	51.980	49.282
Total	28714689	630563		100.000	100.000

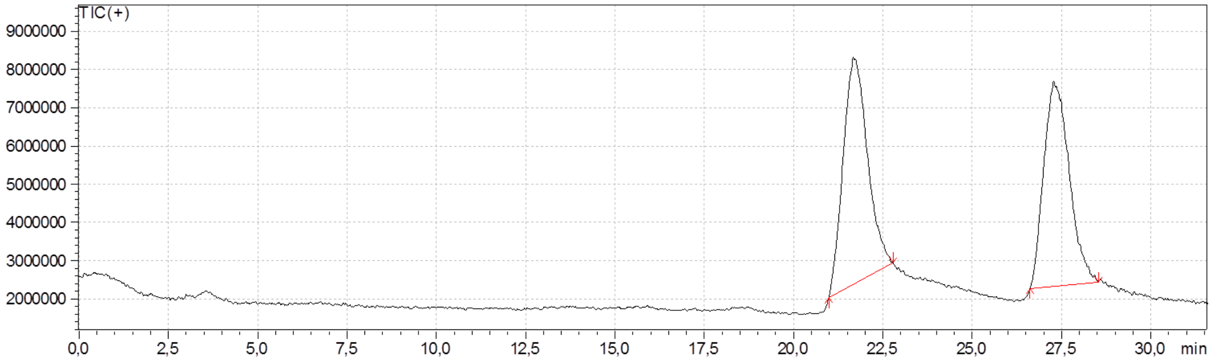

Mass spectra of the first peak (corresponds to *Z*-isomer):

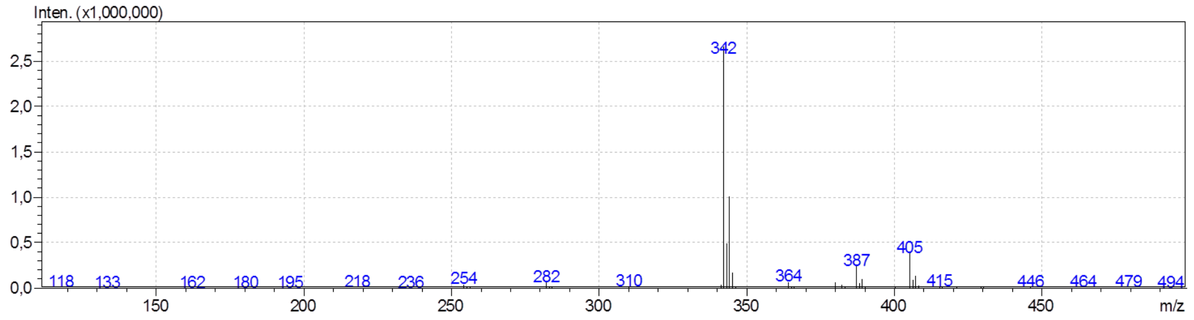

Raw Spectrum: [21.650 → 21.683]; Background: [20.983 → 22.783]; Base Peak: m/z 342.15

Peak#	m/z	Relative Intensity
1	342.15	100.00
2	344.10	37.85
3	343.20	18.49
4	405.15	14.39
5	387.25	8.90
6	345.20	6.29
7	407.15	5.12

Mass spectra of the second peak (corresponds to *E*-isomer):

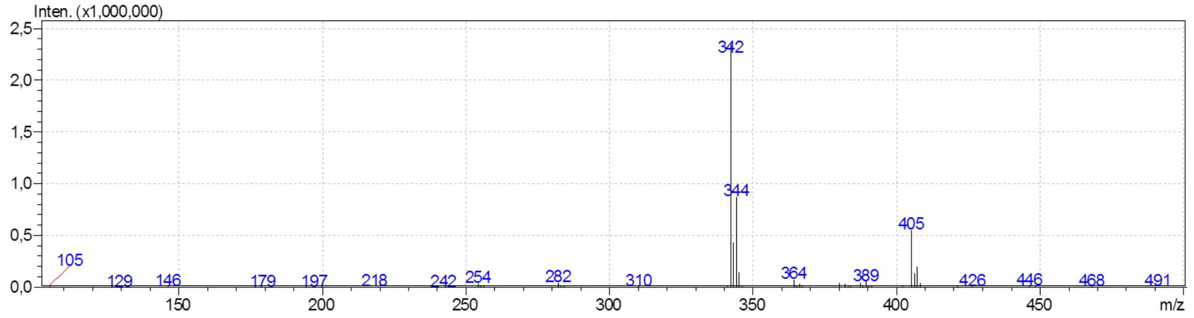

Raw Spectrum: [27.267 → 27.300]; Background: [26.517 → 28.533]; Base Peak: m/z 342.15

Peak#	m/z	Relative Intensity
1	342.15	100.00
2	344.15	37.34
3	405.15	23.69
4	343.20	18.40
5	407.10	8.71
6	345.20	6.08
7	406.20	5.95

Kinetics data

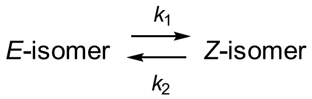

$$K = \frac{k_1}{k_2} = \frac{Z_{eq}}{E_{eq}}$$

E-**2a** → *Z*-**2a** isomerization

Equilibrium ratio of isomers – 32/68 (*E*/*Z*). *K* = 68/32 = 2.125

Corrected experimental data:

Time, s	*E*-isomer, %
0	100.00
400	81.77
800	74.60
1200	66.77
1600	57.52
2000	51.85
2400	49.42
2800	45.34
3200	41.97
3600	39.51
4000	37.71
4400	36.25
4800	34.32
5200	34.13
5600	33.59
6000	32.98
6400	32.17

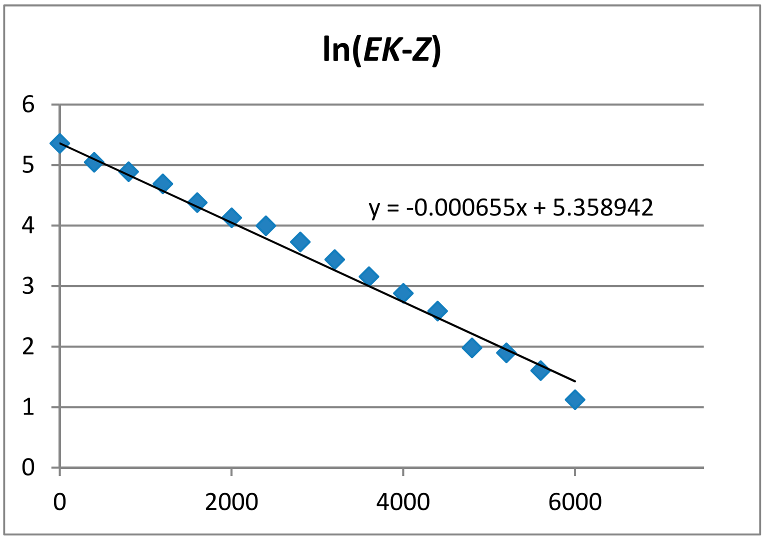

$$k_2 = \frac{1}{t(1+K)} \cdot \ln\frac{E_1K - Z_1}{E_2K - Z_2} = \frac{1}{t(1+K)} \cdot (\ln(E_1K - Z_1) - \ln(E_2K - Z_2))$$

$$y(500) = -0.000655 \cdot 500 + 5.358942 = 5.031$$

$$y(1000) = -0.000655 \cdot 1000 + 5.358942 = 4.704$$

$$\frac{1}{t(1+K)} = \frac{1}{500 \cdot (1 + 2.125)} = 0.00064\ s^{-1}$$

$$k_2 = 0.00064 \cdot (5.031 - 4.704) = 0.210 \cdot 10^{-3}\ s^{-1}$$

$$k_1 = K \cdot k_2 = 2.125 \cdot 0.210 \cdot 10^{-3} = 0.445 \cdot 10^{-3}\ s^{-1}$$

E-**2b** → Z-**2b** isomerization

Equilibrium ratio of isomers – 48/52 (E/Z). K = 52/48 = 1.083

Corrected data:

Time, s	E-isomer, %
0	100.00
600	98.91
1200	92.24
1800	88.43
2400	84.36
3000	80.23
3600	77.52
4200	73.86
4800	72.22
5400	68.55
6000	66.95
6600	70.86
7200	63.51

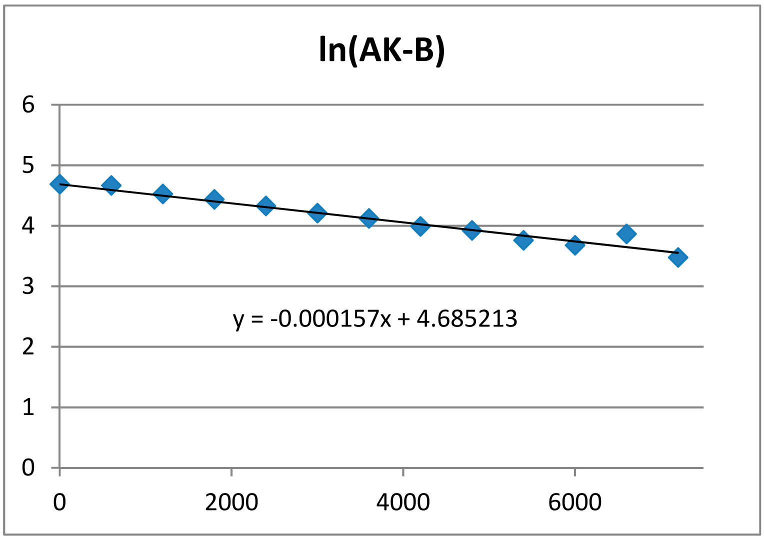

$$k_2 = \frac{1}{t(1+K)} \cdot \ln\frac{E_1K - Z_1}{E_2K - Z_2} = \frac{1}{t(1+K)} \cdot (\ln(E_1K - Z_1) - \ln(E_2K - Z_2))$$

$$y(500) = -0{,}000157 \cdot 500 \ + \ 4{,}685213 = 4.607$$

$$y(1000) = -0{,}000157 \cdot 1000 \ + \ 4{,}685213 = 4.528$$

$$\frac{1}{t(1+K)} = \frac{1}{500 \cdot (1 + 1.083)} = 0.00096\ s^{-1}$$

$$k_2 = \ 0.00096 \cdot (4.607 - 4.528) = 0.075 \cdot 10^{-3}\ s^{-1}$$

$$k_1 = \ K \cdot k_2 = 1.083 \cdot 0.075 \cdot 10^{-3} = 0.082 \cdot 10^{-3}\ s^{-1}$$

E-**2c** → *Z*-**2c** isomerization

Equilibrium ratio of isomers – 31/68 (*E*/*Z*). *K* = 69/31 = 2.226

Corrected experimental data:

Time, s	*E*-isomer, %
0	100.00
600	81.07
1200	74.10
1800	65.12
2400	58.41
3000	50.64
3600	46.61
4200	42.41
4800	39.08
5400	38.80
6000	35.13
6600	34.73

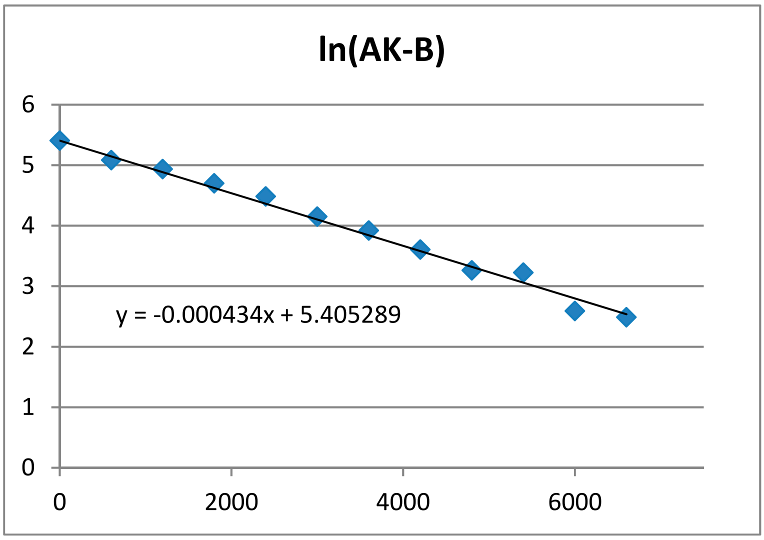

$$k_2 = \frac{1}{t(1+K)} \cdot \ln\frac{E_1K - Z_1}{E_2K - Z_2} = \frac{1}{t(1+K)} \cdot (\ln(E_1K - Z_1) - \ln(E_2K - Z_2))$$

$$y(500) = -0.000434 \cdot 500 + 5.405289 = 5.188$$

$$y(1000) = -0.000434 \cdot 1000 + 5.405289 = 4.971$$

$$\frac{1}{t(1+K)} = \frac{1}{500 \cdot (1 + 2.226)} = 0.00062\ s^{-1}$$

$$k_2 = 0.00062 \cdot (5.188 - 4.971) = 0.135 \cdot 10^{-3}\ s^{-1}$$

$$k_1 = K \cdot k_2 = 2.226 \cdot 0.135 \cdot 10^{-3} = 0.300 \cdot 10^{-3}\ s^{-1}$$

E-**2d** → Z-**2d** isomerization

Equilibrium ratio of isomers – 71/29 (E/Z). K = 29/71 = 0.408

Corrected experimental data:

Time, s	E-isomer, %
0	100.00
600	88.67
1200	84.39
1800	80.07
2400	76.72
3000	74.11
3600	73.12

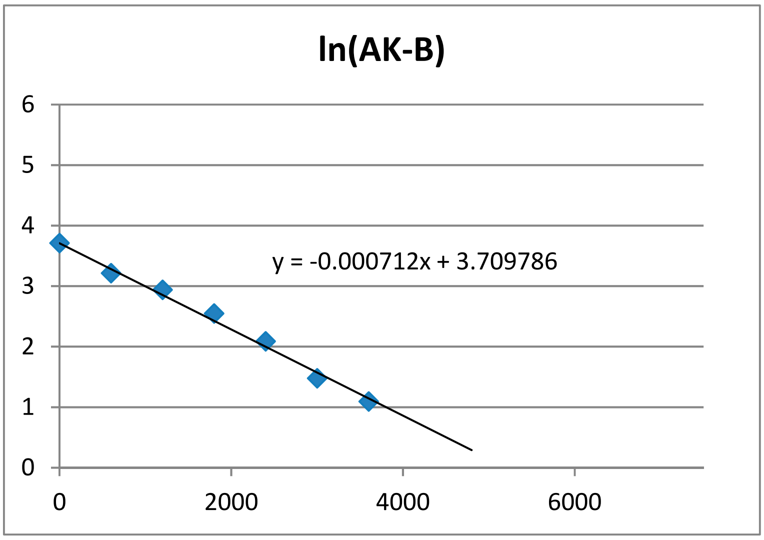

$$k_2 = \frac{1}{t(1+K)} \cdot \ln\frac{E_1K - Z_1}{E_2K - Z_2} = \frac{1}{t(1+K)} \cdot (\ln(E_1K - Z_1) - \ln(E_2K - Z_2))$$

$$y(500) = -0.000712 \cdot 500 + 3.709786 = 3.354$$

$$y(1000) = -0.000712 \cdot 1000 + 3.709786 = 2.998$$

$$\frac{1}{t(1+K)} = \frac{1}{500 \cdot (1+0.408)} = 0.00142\ s^{-1}$$

$$k_2 = 0.00142 \cdot (3.354 - 2.998) = 0.506 \cdot 10^{-3}\ s^{-1}$$

$$k_1 = K \cdot k_2 = 0.408 \cdot 0.506 \cdot 10^{-3} = 0.206 \cdot 10^{-3}\ s^{-1}$$

E-**2e** → Z-**2e** isomerization

Equilibrium ratio of isomers – 76/24 (E/Z). $K = 24/76 = 0.316$

Corrected experimental data:

Time, s	E-isomer, %
0	100.00
440	94.60
880	92.88
1320	89.31
1760	87.36
2200	85.73
2640	85.62
3080	83.41
3520	83.15
3960	82.06
4400	80.24
4840	79.97
5280	82.23
5720	79.24
6160	79.40
6600	75.01

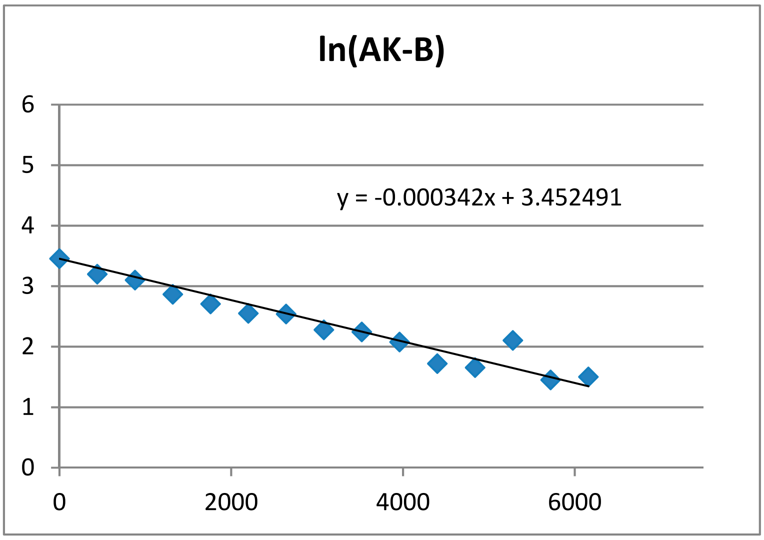

$$k_2 = \frac{1}{t(1+K)} \cdot \ln\frac{E_1K - Z_1}{E_2K - Z_2} = \frac{1}{t(1+K)} \cdot (\ln(E_1K - Z_1) - \ln(E_2K - Z_2))$$

$$y(500) = -0.000342 \cdot 500 + 3.452491 = 3.281$$

$$y(1000) = -0.000342 \cdot 1000 + 3.452491 = 3.110$$

$$\frac{1}{t(1+K)} = \frac{1}{500 \cdot (1+0.316)} = 0.00152\ s^{-1}$$

$$k_2 = 0.00152 \cdot (3.281 - 3.110) = 0.260 \cdot 10^{-3}\ s^{-1}$$

$$k_1 = K \cdot k_2 = 0.316 \cdot 0.260 \cdot 10^{-3} = 0.082 \cdot 10^{-3}\ s^{-1}$$

E-**2f** → *Z*-**2f** isomerization

Equilibrium ratio of isomers – 69/31 (*E*/*Z*). $K = 24/76 = 0.449$

Corrected experimental data:

Time, s	*E*-isomer, %
0	100.00
440	95.04
880	91.08
1320	88.03
1760	84.26
2200	82.52
2640	81.13
3080	78.50
3520	78.64
3960	75.22
4400	73.47
4840	73.65
5280	74.56
5720	71.30
6160	70.88

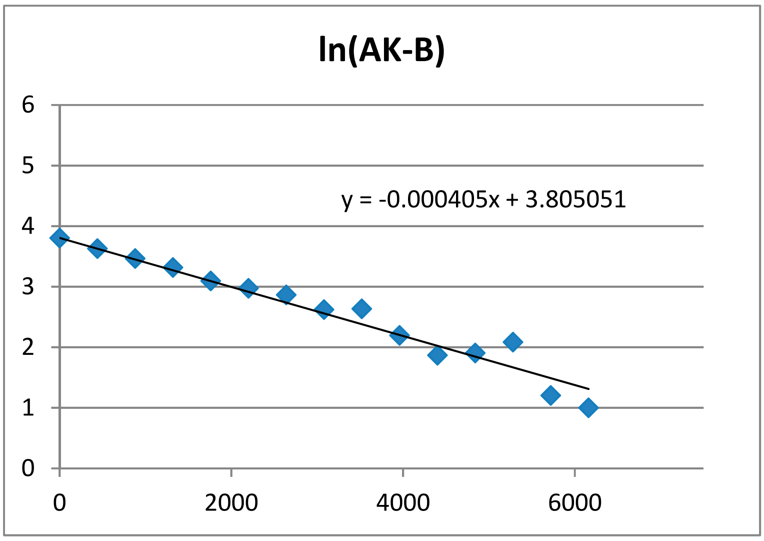

$$k_2 = \frac{1}{t(1+K)} \cdot \ln\frac{E_1K - Z_1}{E_2K - Z_2} = \frac{1}{t(1+K)} \cdot (\ln(E_1K - Z_1) - \ln(E_2K - Z_2))$$

$$y(500) = -0.000405 \cdot 500 + 3.805051 = 3.602$$

$$y(1000) = -0.000405 \cdot 1000 + 3.805051 = 3.400$$

$$\frac{1}{t(1+K)} = \frac{1}{500 \cdot (1+0.449)} = 0.00138\ s^{-1}$$

$$k_2 = \ 0.00138 \cdot (3.602 - 3.400) = 0.280 \cdot 10^{-3}\ s^{-1}$$

$$k_1 = \ K \cdot k_2 = 0.449 \cdot 0.279 \cdot 10^{-3} = 0.126 \cdot 10^{-3}\ s^{-1}$$

E-**3c** → Z-**3c** isomerization

Equilibrium ratio of isomers – 30/70 (E/Z). K = 70/30 = 2.333

Corrected experimental data:

Time, s	E-isomer, %
0	100.00
360	88.27
720	79.39
1080	70.27
1440	64.76
1800	60.05
2160	53.17
2520	49.38
2880	45.08
3240	43.01
3600	37.53
3960	37.70
4320	34.76
4680	33.17

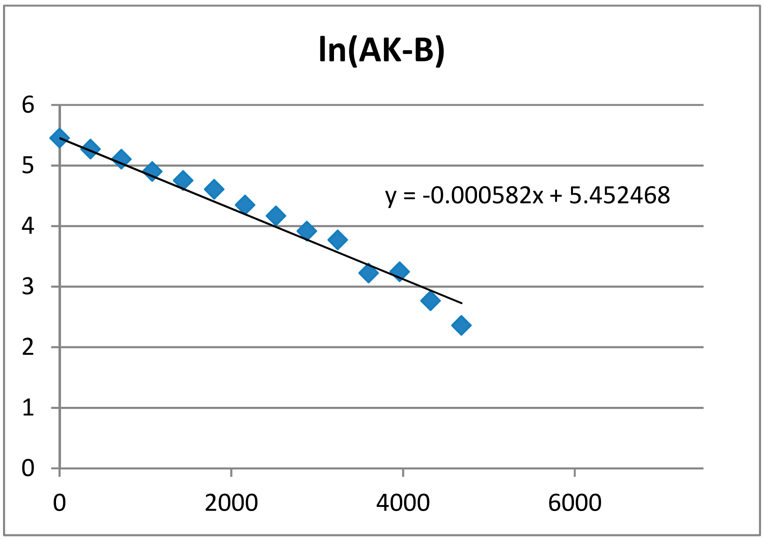

$$k_2 = \frac{1}{t(1+K)} \cdot \ln\frac{E_1K - Z_1}{E_2K - Z_2} = \frac{1}{t(1+K)} \cdot (\ln(E_1K - Z_1) - \ln(E_2K - Z_2))$$

$$y(500) = -0.000582 \cdot 500 + 5.452468 = 5.161$$

$$y(1000) = -0.000582 \cdot 1000 + 5.452468 = 4.870$$

$$\frac{1}{t(1+K)} = \frac{1}{500 \cdot (1 + 2.333)} = 0.0006\ s^{-1}$$

$$k_2 = 0.0006 \cdot (5.161 - 4.870) = 0.175 \cdot 10^{-3}\ s^{-1}$$

$$k_1 = K \cdot k_2 = 2.333 \cdot 0.175 \cdot 10^{-3} = 0.407 \cdot 10^{-3}\ s^{-1}$$

E-**3d** → *Z*-**3d** isomerization

Equilibrium ratio of isomers – 75/25 (*E*/*Z*). $K = 25/75 = 0.333$

Corrected experimental data:

Time, s	*E*-isomer, %
0	100.00
300	96.12
600	89.94
900	84.96
1200	84.25
1500	82.58
1800	80.97
2100	78.93
2400	76.95
2700	78.73

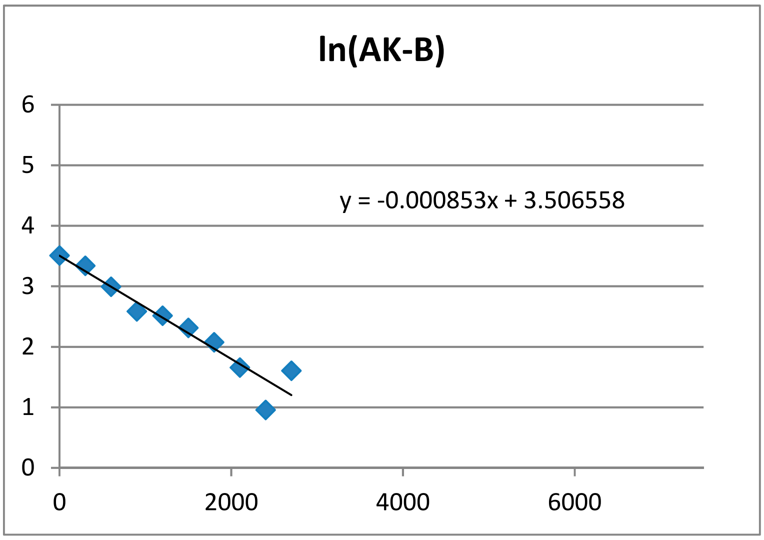

$$k_2 = \frac{1}{t(1+K)} \cdot \ln\frac{E_1K - Z_1}{E_2K - Z_2} = \frac{1}{t(1+K)} \cdot (\ln(E_1K - Z_1) - \ln(E_2K - Z_2))$$

$$y(500) = -0.000853 \cdot 500 + 3.506558 = 3.080$$

$$y(1000) = -0.000853 \cdot 1000 + 3.506558 = 2.654$$

$$\frac{1}{t(1+K)} = \frac{1}{500 \cdot (1 + 0.333)} = 0.0015\ s^{-1}$$

$$k_2 = \ 0.0015 \cdot (3.080 - 2.654) = 0.640 \cdot 10^{-3}\ s^{-1}$$

$$k_1 = \ K \cdot k_2 = 0.333 \cdot 0.640 \cdot 10^{-3} = 0.213 \cdot 10^{-3}\ s^{-1}$$

E-**3e** → Z-**3e** isomerization

Equilibrium ratio of isomers – 77/23 (E/Z). K = 23/77 = 0.299

Corrected experimental data:

Time, s	E-isomer, %
0	100.00
300	96.59
600	89.78
900	89.43
1200	87.82
1500	87.80
1800	84.78
2100	84.12
2400	-
2700	84.03
3000	79.89
3300	80.88
3600	79.36
3900	80.65

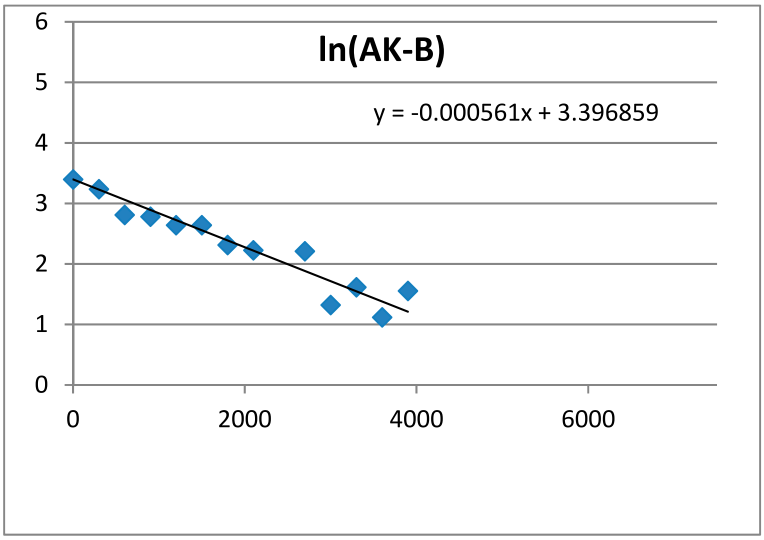

$$k_2 = \frac{1}{t(1+K)} \cdot \ln\frac{E_1K - Z_1}{E_2K - Z_2} = \frac{1}{t(1+K)} \cdot (\ln(E_1K - Z_1) - \ln(E_2K - Z_2))$$

$$y(500) = -0.000561 \cdot 500 + 3.396859 = 3.116$$

$$y(1000) = -0.000561 \cdot 1000 + 3.396859 = 2.836$$

$$\frac{1}{t(1+K)} = \frac{1}{500 \cdot (1+0.449)} = 0.00154\ s^{-1}$$

$$k_2 = \ 0.00154 \cdot (3.116 - 2.836) = 0.432 \cdot 10^{-3}\ s^{-1}$$

$$k_1 = \ K \cdot k_2 = 0.299 \cdot 0.432 \cdot 10^{-3} = 0.129 \cdot 10^{-3}\ s^{-1}$$

X-ray data

The crystal structures of *E*-**2d**, *Z*-**2d**, *E*-**2e** and *Z*-**2e** were determined by single crystal X-ray diffraction analysis. Crystals were fixed on micro mounts and placed on a Rigaku Oxford Diffraction SuperNova Atlas diffractometer. Diffraction data were collected at a temperature of 100K using monochromated microfocused Cu$K\alpha$ radiation.

The data were integrated and corrected for background, Lorentz, and polarization effects. An empirical absorption correction based on spherical harmonics implemented in the SCALE3 ABSPACK algorithm was applied in the *CrysAlisPro* program [4]. The unit-cell parameters were refined by the least-squares techniques. The structures were solved by the dual-space algorithm and refined using the *SHELX* programs [5, 6] incorporated in the *OLEX2* program package [7].

The final models included coordinates and anisotropic displacement parameters for all non-H atoms. The carbon- and nitrogen-bound H atoms were placed in the calculated positions and were included in the refinement in the riding model approximation with U_{iso}(H) set to $1.5U_{eq}$(C) and C–H 0.96 Å for the CH_3 groups, U_{iso}(H) set to $1.2U_{eq}$(C) and C–H 0.93 Å for the CH groups, and U_{iso}(H) set to $1.2U_{eq}$(N) and N–H 0.86 Å for the NH groups.

Supplementary crystallographic data for this paper were deposited at Cambridge Crystallographic Data Centre (CCDC 2171213–2171216) and can be obtained free of charge via www.ccdc.cam.ac.uk/structures/ .

Compound *E*-**2d**

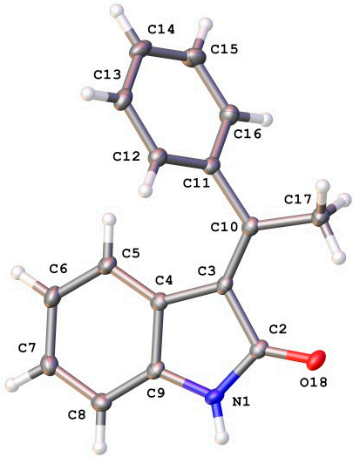

Formula	$C_{16}H_{13}NO$
Molecular weight	235.27
Crystal System	Monoclinic
a (Å)	26.0750(17)
b (Å)	8.42919(17)
c (Å)	14.9426(9)
α (°)	90
β (°)	134.115(11)
γ (°)	90
V (Å^3)	2357.9(4)
Space group	*C*2/*c*
μ (mm^{-1})	0.653
Z	8
D_{calc} (g/cm^3)	1.326

Crystal size (mm^3)	0.28×0.22×0.15
Total reflections	6196
Unique reflections	2220
Angle range 2θ (°)	9.45–140.00
Reflections with $\|F_o\| \geq 4\sigma_F$	1991
R_{int}	0.0207
R_σ	0.0203
R_1 ($\|F_o\| \geq 4\sigma_F$)	0.0346
wR_2 ($\|F_o\| \geq 4\sigma_F$)	0.0899
R_1 (all data)	0.0393
wR_2 (all data)	0.0942
S	1.064
ρ_{min}, ρ_{max}, e/Å^3	–0.320, 0.349
CCDC	2171213

$R_1 = \Sigma||F_o| - |F_c||/\Sigma|F_o|$;

$wR_2 = \{\Sigma[w(F_o^2 - F_c^2)^2]/\Sigma[w(F_o^2)^2]\}^{1/2}$;

$w = 1/[\sigma^2(F_o^2)+(aP)^2 + bP]$, where $P = (F_o^2 + 2F_c^2)/3$;

$s = \{\Sigma[w(F_o^2 - F_c^2)]/(n - p)\}^{1/2}$ where n is the number of reflections and p is the number of refinement parameters.

Compound *Z*-**2d**

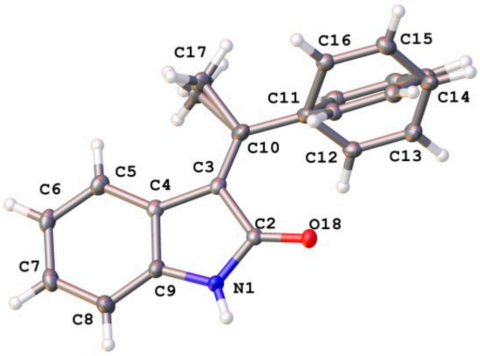

Formula	$C_{16}H_{13}NO$
Molecular weight	235.27
Crystal System	Monoclinic
a (Å)	24.3428(10)
b (Å)	5.0167(2)
c (Å)	21.3304(11)
α (°)	90
β (°)	114.337(4)
γ (°)	90
V (Å^3)	2373.4(2)
Space group	$C2/c$
μ (mm^{-1})	0.648
Z	8
D_{calc} (g/cm^3)	1.317

Crystal size (mm^3)	0.40×0.25×0.19
Total reflections	11272
Unique reflections	2240
Angle range 2θ (°)	7.97–140.00
Reflections with $\|F_o\|\geq4\sigma_F$	2068
R_{int}	0.0495
R_σ	0.0298
R_1 ($\|F_o\| \geq 4\sigma_F$)	0.0390
wR_2 ($\|F_o\| \geq 4\sigma_F$)	0.1005
R_1 (all data)	0.0425
wR_2 (all data)	0.1033
S	1.047
ρ_{min}, ρ_{max}, e/Å^3	–0.244, 0.218
CCDC	2171214

$R_1 = \Sigma||F_o| - |F_c||/\Sigma|F_o|$;

$wR_2 = \{\Sigma[w(F_o^2 - F_c^2)^2]/\Sigma[w(F_o^2)^2]\}^{1/2}$;

$w = 1/[\sigma^2(F_o^2)+(aP)^2 + bP]$, where $P = (F_o^2 + 2F_c^2)/3$;

$s = \{\Sigma[w(F_o^2 - F_c^2)]/(n - p)\}^{1/2}$ where n is the number of reflections and p is the number of refinement parameters.

Compound *E*-**2e**

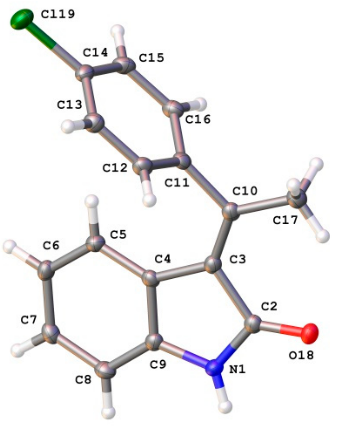

Formula	$C_{16}H_{12}ClNO$
Molecular weight	269.72
Crystal System	Monoclinic
a (Å)	12.6163(6)
b (Å)	8.3537(2)
c (Å)	13.0179(6)
α (°)	90
β (°)	113.483(5)
γ (°)	90
V (Å^3)	1258.37(10)
Space group	$P2_1/n$
μ (mm^{-1})	2.595
Z	4
D_{calc} (g/cm^3)	1.424

Crystal size (mm^3)	0.23×0.21×0.19
Total reflections	5564
Unique reflections	2322
Angle range 2θ (°)	8.25–140.00
Reflections with $\|F_o\|\geq 4\sigma_F$	2030
R_{int}	0.0234
R_σ	0.0257
R_1 ($\|F_o\| \geq 4\sigma_F$)	0.0337
wR_2 ($\|F_o\| \geq 4\sigma_F$)	0.0825
R_1 (all data)	0.0411
wR_2 (all data)	0.0880
S	1.029
ρ_{min}, ρ_{max}, e/Å^3	–0.400, 0.277
CCDC	2171215

$R_1 = \Sigma||F_o| - |F_c||/\Sigma|F_o|$;

$wR_2 = \{\Sigma[w(F_o^2 - F_c^2)^2]/\Sigma[w(F_o^2)^2]\}^{1/2}$;

$w = 1/[\sigma^2(F_o^2)+(aP)^2 + bP]$, where $P = (F_o^2 + 2F_c^2)/3$;

$s = \{\Sigma[w(F_o^2 - F_c^2)]/(n - p)\}^{1/2}$ where n is the number of reflections and p is the number of refinement parameters.

Compound *Z*-**2e**

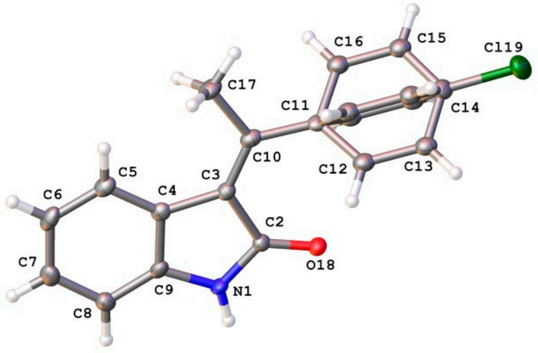

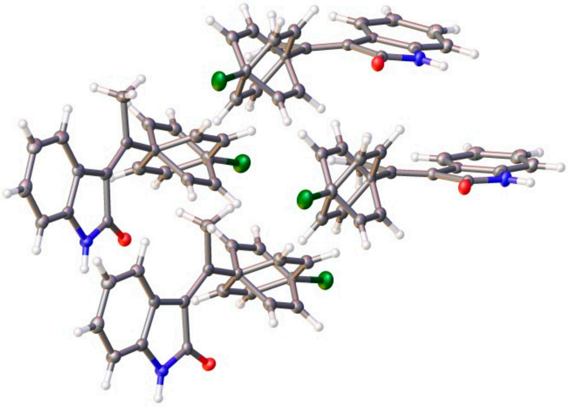

Formula	$C_{16}H_{12}ClNO$
Molecular weight	269.72
Crystal System	Triclinic
a (Å)	9.9445(3)
b (Å)	13.8371(4)
c (Å)	19.4290(7)
α (°)	79.807(3)
β (°)	82.804(3)
γ (°)	79.699(3)
V (Å^3)	2576.76(15)
Space group	P-1
μ (mm^{-1})	2.534
Z	8
D_{calc} (g/cm^3)	1.391

Crystal size (mm^3)	0.59×0.13×0.08
Total reflections	25399
Unique reflections	9653
Angle range 2θ (°)	6.58–140.00
Reflections with $\|F_o\|\geq 4\sigma_F$	7208
R_{int}	0.0668
R_σ	0.0643
R_1 ($\|F_o\| \geq 4\sigma_F$)	0.0669
wR_2 ($\|F_o\| \geq 4\sigma_F$)	0.1695
R_1 (all data)	0.0889
wR_2 (all data)	0.1895
S	1.042
ρ_{min}, ρ_{max}, e/Å^3	–0.658, 0.989
CCDC	2171216

$R_1 = \Sigma||F_o| - |F_c||/\Sigma|F_o|$;

$wR_2 = \{\Sigma[w(F_o^2 - F_c^2)^2]/\Sigma[w(F_o^2)^2]\}^{1/2}$;

$w = 1/[\sigma^2(F_o^2)+(aP)^2 + bP]$, where $P = (F_o^2 + 2F_c^2)/3$;

$s = \{\Sigma[w(F_o^2 - F_c^2)]/(n - p)\}^{1/2}$ where n is the number of reflections and p is the number of refinement parameters.

References

1. Crestini C, Saladino R. A new efficient and mild synthesis of 2-oxindoles by one-pot Wolff-Kishner like reduction of isatin derivatives. *Synth Commun,* 1994, 24: 2835–2841.

2. Coda AC, Invernizzi AG, Righetti PP, Tacconi G, Gatti G. (*Z*)- and (*E*)-Arylidene-1,3-dihydroindol-2-ones: configuration, conformation, and infrared carbonyl stretching frequencies. *J Chem Soc, Perkin Trans 2*, 1984, 615–619.

3. Olgen S, Akaho E, Nebioglu D. Synthesis and anti-tyrosine kinase activity of 3-(substituted-benzylidene)-1,3-dihydro-indolin derivatives: investigation of their role against p60c-Src receptor tyrosine kinase with the application of receptor docking studies. *Farmaco*, 2005, 60: 497–506.

4. CrysAlisPro, Rigaku Oxford Diffraction, Version 1.171.41.104a, 2021.

5. Sheldrick GM. SHELXT - integrated space-group and crystal-structure determination. *Acta Crystallogr A Found Adv*, 2015, 71(Pt 1): 3–8.

6. Sheldrick GM. Crystal structure refinement with SHELXL. *Acta Crystallogr C Struct Chem*, 2015, 71(Pt 1): 3–8.

7. Dolomanov OV, Bourhis LJ, Gildea RJ, Howard JAK, Puschmann H. *OLEX2:* A complete structure solution, refinement and analysis program. *J Appl Cryst*, 2009, 42: 339–341.
